# Towards 6G wireless communication networks: vision, enabling technologies, and new paradigm shifts

**DOI:** 10.1007/s11432-020-2955-6

**Published:** 2020-11-24

**Authors:** Xiaohu You, Cheng-Xiang Wang, Jie Huang, Xiqi Gao, Zaichen Zhang, Mao Wang, Yongming Huang, Chuan Zhang, Yanxiang Jiang, Jiaheng Wang, Min Zhu, Bin Sheng, Dongming Wang, Zhiwen Pan, Pengcheng Zhu, Yang Yang, Zening Liu, Ping Zhang, Xiaofeng Tao, Shaoqian Li, Zhi Chen, Xinying Ma, Chih-Lin I, Shuangfeng Han, Ke Li, Chengkang Pan, Zhimin Zheng, Lajos Hanzo, Xuemin (Sherman) Shen, Yingjie Jay Guo, Zhiguo Ding, Harald Haas, Wen Tong, Peiying Zhu, Ganghua Yang, Jun Wang, Erik G. Larsson, Hien Quoc Ngo, Wei Hong, Haiming Wang, Debin Hou, Jixin Chen, Zhe Chen, Zhangcheng Hao, Geoffrey Ye Li, Rahim Tafazolli, Yue Gao, H. Vincent Poor, Gerhard P. Fettweis, Ying-Chang Liang

**Affiliations:** 1 National Mobile Communications Research Laboratory, School of Information Science and Engineering, Southeast University, Nanjing, 210096 China; 2 Purple Mountain Laboratories, Nanjing, 211111 China; 3 Shanghai Institute of Fog Computing Technology (SHIFT), ShanghaiTech University, Shanghai, 201210 China; 4 Research Center for Network Communication, Peng Cheng Laboratory, Shenzhen, 518000 China; 5 State Key Laboratory of Networking and Switching Technology, Beijing University of Posts and Telecommunications, Beijing, 100876 China; 6 National Engineering Laboratory for Mobile Network Technologies, Beijing University of Posts and Telecommunications, Beijing, 100876 China; 7 National Key Laboratory of Science and Technology on Communications, University of Electronic Science and Technology of China (UESTC), Chengdu, 611731 China; 8 China Mobile Research Institute, Beijing, 100053 China; 9 School of Electronics and Computer Science, University of Southampton, Southampton, SO17 1BJ UK; 10 Department of Electrical and Computer Engineering, University of Waterloo, Waterloo, N2L 3G1 Canada; 11 Global Big Data Technologies Centre (GBDTC), University of Technology Sydney, Sydney, NSW 2007 Australia; 12 School of Electrical and Electronic Engineering, The University of Manchester, Manchester, M13 9PL UK; 13 LiFi Research and Development Centre, Institute for Digital Communications, School of Engineering, The University of Edinburgh, Edinburgh, EH9 3JL UK; 14 Huawei Technologies Canada Co., Ltd., Ottawa, K2K 3J1 Canada; 15 Huawei Technologies, Shanghai, 201206 China; 16 Huawei Technologies, Hangzhou, 310007 China; 17 Department of Electrical Engineering (ISY), Linköping University, Linköping, 581 83 Sweden; 18 Institute of Electronics, Communications & Information Technology (ECIT), Queen’s University Belfast, Belfast, BT3 9DT UK; 19 State Key Laboratory of Millimeter Waves, School of Information Science and Engineering, Southeast University, Nanjing, 210096 China; 20 School of Electrical and Computer Engineering, Georgia Institute of Technology, Atlanta, GA 30332 USA; 21 5G Innovation Centre, University of Surrey, Guildford, GU2 7XH UK; 22 Princeton University, Princeton, NJ 08544 USA; 23 Vodafone Chair Mobile Communications Systems, Technische Universität Dresden, Dresden, 01069 Germany; 24 Center for Intelligent Networking and Communications (CINC), University of Electronic Science and Technology of China (UESTC), Chengdu, 611731 China

**Keywords:** 6G, vision, network architecture, air interface and transmission technologies, space-air-ground-sea integrated network, all spectra, artificial intelligence, network security

## Abstract

The fifth generation (5G) wireless communication networks are being deployed worldwide from 2020 and more capabilities are in the process of being standardized, such as mass connectivity, ultra-reliability, and guaranteed low latency. However, 5G will not meet all requirements of the future in 2030 and beyond, and sixth generation (6G) wireless communication networks are expected to provide global coverage, enhanced spectral/energy/cost efficiency, better intelligence level and security, etc. To meet these requirements, 6G networks will rely on new enabling technologies, i.e., air interface and transmission technologies and novel network architecture, such as waveform design, multiple access, channel coding schemes, multi-antenna technologies, network slicing, cell-free architecture, and cloud/fog/edge computing. Our vision on 6G is that it will have four new paradigm shifts. First, to satisfy the requirement of global coverage, 6G will not be limited to terrestrial communication networks, which will need to be complemented with non-terrestrial networks such as satellite and unmanned aerial vehicle (UAV) communication networks, thus achieving a space-air-ground-sea integrated communication network. Second, all spectra will be fully explored to further increase data rates and connection density, including the sub-6 GHz, millimeter wave (mmWave), terahertz (THz), and optical frequency bands. Third, facing the big datasets generated by the use of extremely heterogeneous networks, diverse communication scenarios, large numbers of antennas, wide bandwidths, and new service requirements, 6G networks will enable a new range of smart applications with the aid of artificial intelligence (AI) and big data technologies. Fourth, network security will have to be strengthened when developing 6G networks. This article provides a comprehensive survey of recent advances and future trends in these four aspects. Clearly, 6G with additional technical requirements beyond those of 5G will enable faster and further communications to the extent that the boundary between physical and cyber worlds disappears.

**Citation** You X H, Wang C-X, Huang J, et al. Towards 6G wireless communication networks: vision, enabling technologies, and new paradigm shifts. Sci China Inf Sci, 2021, 64(1): 110301, https://doi.org/10.1007/s11432-020-2955-6

### 1 Introduction

The fifth generation (5G) wireless communication networks are being standardized and deployed worldwide from 2020. Three major communication scenarios of 5G are enhanced mobile broadband (eMBB), massive machine type communications (mMTC), and ultra-reliable and low latency communications (uRLLC). The key capabilities include 20 Gbps peak data rate, 0.1 Gbps user experienced data rate, 1 ms end-to-end latency, supporting 500 km/h mobility, 1 million devices/km$^2$ connection density, 10 Mbps/m$^2$ area traffic capacity, 3 times spectrum efficiency, and 100 times energy efficiency compared to the fourth generation (4G) wireless communication systems. Various key technologies such as the millimeter wave (mmWave), massive multiple-input multiple-output (MIMO), and ultra-dense network (UDN) have been proposed to achieve the goal of 5G [1].

However, 5G will not meet all requirements of the future in 2030+. Researchers now start to focus on the sixth generation (6G) wireless communication networks. One of the main distinguishing features of 5G is low latency or more specifically guaranteed (deterministic) latency, which needs deterministic networking (DetNet) to guarantee end-to-end latency with punctuality and accuracy that future use cases demand. The 6G will have additional requirements of high time and phase synchronization accuracy beyond what 5G can deliver. Additionally, 6G will have to provide near 100% geographical coverage, sub-centimeter geo-location accuracy and millisecond geo-location update rate to meet use cases. As 5G networks are still limited to some typical scenarios, remote areas such as villages and motorways are not well covered, which limits some applications such as driverless vehicles. Non-terrestrial and specifically satellite communication networks are needed to complement the terrestrial networks for cost-effective, seamless, and ubiquitous service availability. Unmanned aerial vehicle (UAV) communication network is important for fast response in harsh and difficult environments. Maritime communication network is needed to provide ships with high quality communication services. While mmWave can provide Gbps level transmission data rate in 5G, Tbps level transmission data rate will be needed for applications such as high quality three-dimensional (3D) video, virtual reality (VR), and mix of VR and augmented reality (AR), where terahertz (THz) and optical frequency bands can be candidate bands. Faced with the big datasets generated by using extremely heterogeneous networks, diverse communication scenarios, large numbers of antennas, wide bandwidths, and new service requirements, 6G networks will enable a new range of smart applications with the aid of artificial intelligence (AI) and machine learning (ML) technologies. One automation level is for improving the network performance itself in many aspects, for example, quality of service (QoS), quality of experience (QoE), security, fault management, and energy efficiency.

Up to 5G, traffic on the network is dominated by video or streaming applications. Besides all applications and requirements mentioned above, we can learn from 5G tactile Internet applications [2] that wireless networked control of robotic objects (e.g., automated driving or factory logistics) is a new exciting application for cellular technology, but this also generates new challenges. When analyzing the network traffic generated by these applications, many mobile objects must share sensor as well as control information, which overburdens a centralized control system. Instead, distributed control systems using AI are becoming a focus in research and development. In particular federated learning shows to be a promising approach, where dataset correlation algorithms are distributed over mobile robotic objects and aggregated learning happens over the cloud. Interestingly, this generates a completely new class of network traffic, with large bandwidth and widely varying latency demands. It is highly likely to assume that these and equivalent AI applications will not only overtake but dominate the network traffic demands of 6G. This is untouched soil, which makes it exciting and very challenging at the same time!

In comparison with the 5G network, 6G wireless communication networks are expected to provide much higher spectral/energy/cost efficiency, higher data rate (Tbps), 10 times lower latency, 100 times higher connection density, more intelligence for full automation, sub-centimeter geo-location accuracy, near 100% coverage, and sub-millisecond time synchronization. New air interface and transmission technologies are essential to achieve high spectrum efficiency and energy efficiency, including new waveforms, multiple access approaches, channel coding methods, multi-antenna technologies, and proper combination of all these diversity techniques. In the meanwhile, novel network architectures are needed, for example, software defined network/network functions virtualization (SDN/NFV), dynamic network slicing, service-based architecture (SBA), cognitive service architecture (CSA), and cell-free (CF) architectures.

However, softwarization comes at a cost, as we can learn from 5G deployment. The use of commercial off-the-shelf (COTS) servers versus domain specific chips in a virtualized radio access network (RAN) implies a large increase in energy consumption, countering measures for improving energy efficiency. This results in the current fact that 5G networks consume more power than 4G networks, but of course at a delivery of a higher bandwidth. In contrast, we should deliver networks that at the time of their introduction do not exceed the previous generation's power needs. For 6G we therefore will require a new computing paradigm to support all benefits of softwarization without bearing the costs of energy consumption.

Computing technologies such as the cloud computing, fog computing, and edge computing are important for network resilience, distributed computing and processing, and lower latency and time synchronization. In order to solve the limitations of 5G including the drawback of short-packet, provide the delivery of high-reliability, low-latency services with high data rates, system coverage and Internet of everything (IoE) [3], and to meet the demands of mobile communications of the year 2030 and beyond [4], 6G network should make the human-centric, instead of machine-centric, application-centric, or data-centric, as the vision [5]. To meet these requirements, 6G wireless communication networks will have new paradigm shifts. Our vision of 6G network is illustrated in Figure 1. First of all, 6G wireless communication networks will be space-air-ground-sea integrated networks to provide a complete global coverage. The satellite communication, UAV communication, and maritime communication will largely extend the coverage range of wireless communication networks. To provide a higher data rate, all spectra will be fully explored, including sub-6 GHz, mmWave, THz, and optical frequency bands. To enable full applications, AI and ML technologies will be efficiently combined with 6G wireless communication networks to have a better network management and automation. Furthermore, AI technology can enable the dynamic orchestration of networking, caching, and computing resources to improve the performance of next-generation networks. The last but not the least trend is the strong or endogenous network security for both physical layer and network layer when developing it. Industry verticals, such as cloud VR, Internet of things (IoT) industry automation, cellular vehicle to everything (C-V2X), digital twin body area network, and energy efficient wireless network control and federated learning systems will largely boost the developments of 6G wireless communication networks. An overview of 6G wireless networks is shown in Figure 2, where the performance metrics, application scenarios, enabling technologies, new paradigm shifts, and industry verticals are given.

The organization of this paper is shown in Figure 3. The limitations of 5G, the requirements of 6G, and the 6G vision are introduced in Section 1. In Section 2, the performance metrics and application scenarios of 6G wireless networks, as well as example industry verticals are presented. The enabling

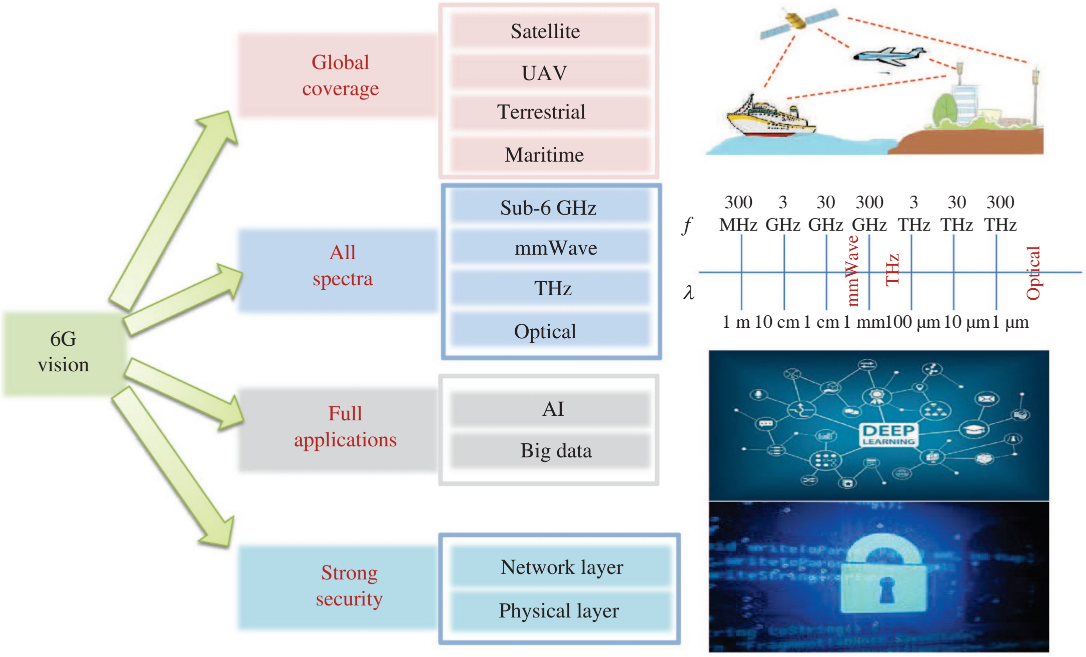

**Figure 1** (Color online) A vision of 6G wireless communication networks.

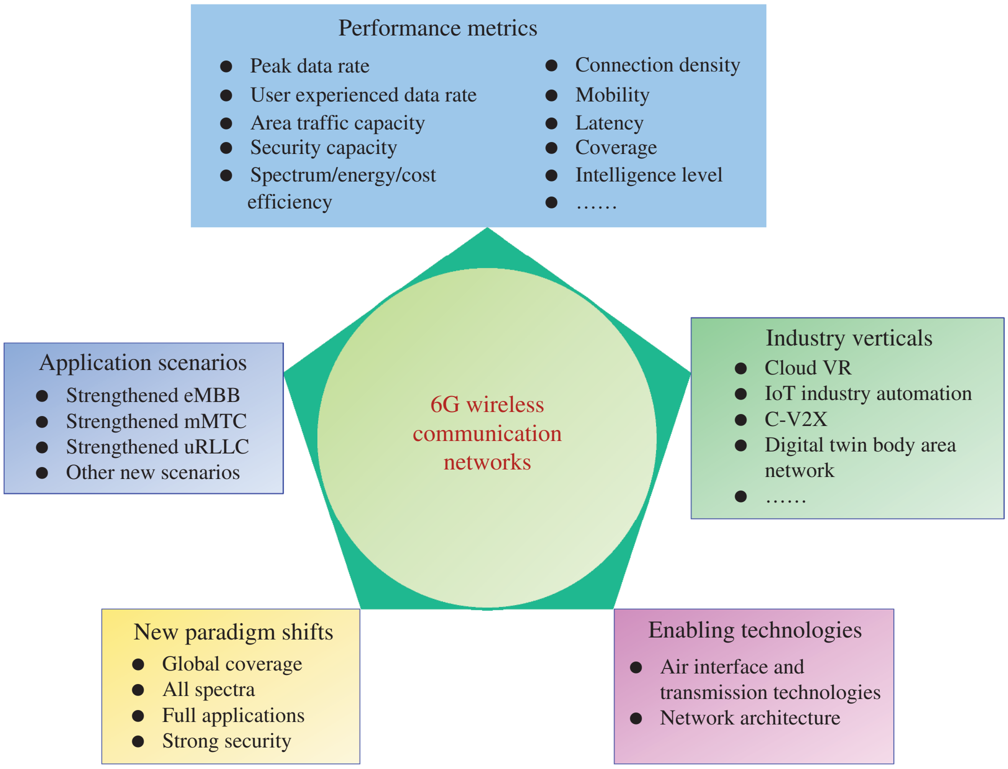

**Figure 2** (Color online) An overview of 6G wireless communication networks.

technologies, including the air interface and transmission technologies, and novel network architectures for 6G wireless networks are given in Section 3. The new paradigm shifts, i.e., the global coverage, all spectra, full applications, and strong security, are presented in Section 4. Conclusions are drawn in Section 5. The abbreviations in this paper can be found in Appendix A.

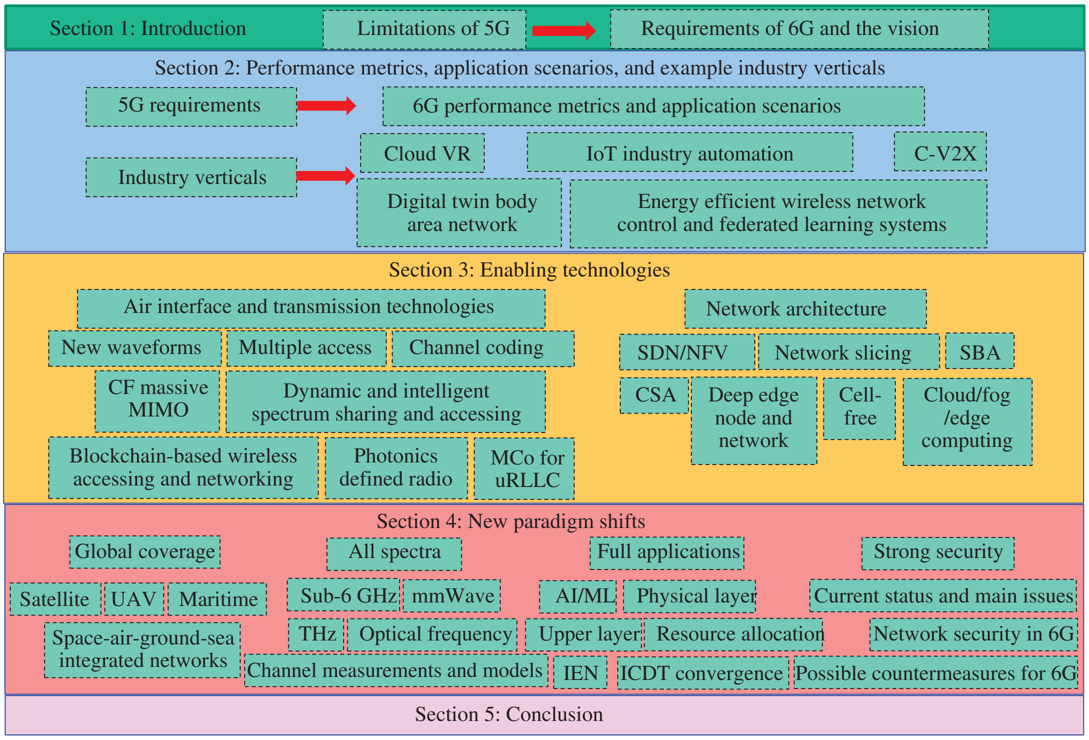

**Figure 3** (Color online) Organization of this paper.

## 2 Performance metrics, application scenarios, and example industry verticals

### 2.1 Performance metrics and application scenarios

Compared to 5G (IMT-2020) [6], 6G networks are expected to achieve superior performance and have more performance metrics, as illustrated in Figure 4. The peak data rate for 5G is 20 Gbps, while for 6G networks it can be 1–10 Tbps with the aid of THz and optical frequency bands. The user experienced data rate can achieve a Gbps-level with these high frequency bands. The area traffic capacity can be more than 1 Gbps/m$^2$. The spectrum efficiency can increase 3–5 times, while the network energy efficiency must increase by more than 100 times compared to 5G to make-up for the increase in data rate by 100 times. This can be achievable by applying AI to achieve much better network management and automation. The connection density will increase 10–100 times owing to the use of extremely heterogeneous networks, diverse communication scenarios, large numbers of antennas, and wide bandwidths. There are multiple types of mobility introduced by satellites, UAVs, and ultra-high-speed trains, which can move with a much higher speed of larger than 500 km/h in comparison to the existing terrestrial terminals. For a selected set of applications, the latency is expected to be less than 1 ms. In addition, other important performance metrics should be introduced, e.g., cost efficiency, security capacity, coverage, and intelligence level [7].

As a comparison, Bell laboratory proposed some key performance metrics in March 2019. The peak data rate is expected to be more than 100 Gbps. The user experienced data rate is 1–10 Gbps. The connection density is $10^7$ devices/km$^2$. The latency is expected to be less than 0.3 ms. The energy efficiency is expected to be 10 times compared to 5G. The capacity is expected to be 10000 times of 5G systems. Other metrics include 10 cm level indoor positioning accuracy and 1 m level outdoor positioning accuracy, and six “9” reliability. In [8], the authors proposed the key performance metrics as $\geqslant 1$ Tbps peak data rate, 1 Gbps user experienced data rate, 10–100 µs latency, 1000 km/h mobility, $10^7$ devices/km$^2$ connection density, 1 Gbps/m$^2$ area traffic capacity, and 10–100 times energy efficiency and 5–10 times spectrum efficiency compared to 5G. The authors in [9] showed a similar vision on the key performance metrics of 6G networks. However, none of these publications has yet taken the kind of traffic and the performance criteria into account which will be demanded to serve distributed AI for implementing wireless network control systems for tactile Internet applications, as mentioned in Section 1. For this field of application, much understanding must be generated over the next years to

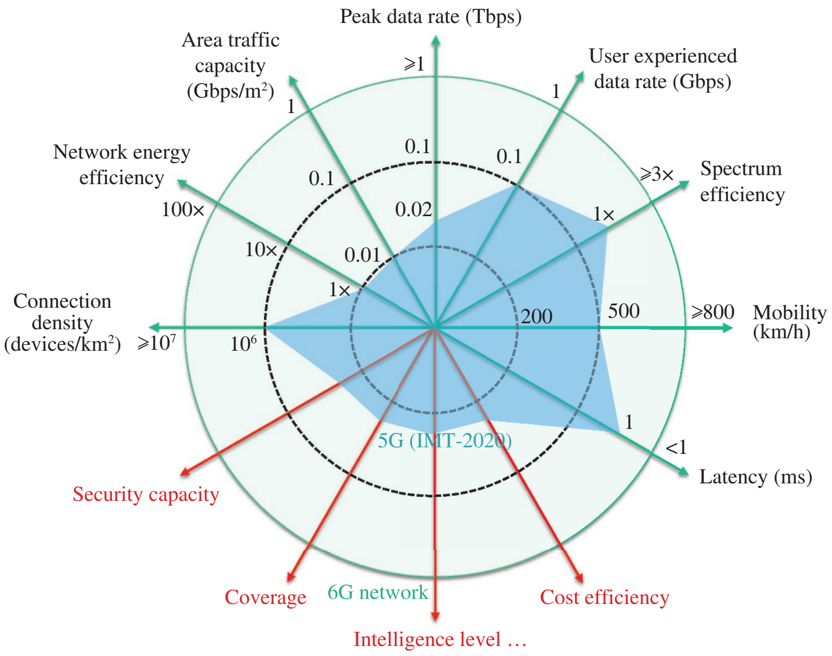

**Figure 4** (Color online) Performance metrics of 6G communication networks.

design and dimension 6G networks to match the upcoming bandwidth, latency, reliability, as well as resilience demands which are set by the applications.

While 5G mainly concentrates on eMBB, mMTC, and uRLLC, 6G networks will largely enhance and extend the application scenarios. In [8], the authors named the enhanced three scenarios as feMBB, umMTC, and enhanced-uRLLC (euRLLC). Several other application scenarios, such as LDHMC, and ELPC, are also promising. In [4], these three scenarios were named as the ubiquitous mobile ultra-broadband (uMUB), ultra-high-speed with low-latency communications (uHSLLC), and ultra-high data density (uHDD), respectively. In [10], apart from the three scenarios, the authors also illustrated other applications, including AI, mobile ultra-wideband, and super IoT.

The international telecommunication union (ITU) released the 6G initial research schedule in February 2020. Its 6G vision and technology trends study are expected to be completed by 2023. The ITU-T focus group technologies for network 2030 (FG NET-2030) was established by ITU-T Study Group 13 at its meeting in Geneva, 16–27 July 2018. It intends to study the capabilities of networks for the year 2030 and beyond, when it is expected to support novel forward-looking scenarios, such as holographic type communications, extremely fast response in critical situations and high-precision communication demands of emerging market verticals.

The potential use cases to widely support the human-centric communication concept in the 2030s contain communications of wearable devices, integrated headsets, implantable sensors, human bond communications, smart cars, smart manufacturing, and Industry 4.0 and robotics [4, 11]. In addition to conventional mobile communication devices (e.g., mobile phones and laptops), taking health-related wearable devices into consideration, the transmit power and frequency band used in these new communication devices should be strict constraints. As these devices will work on the human body and collect required data, the device weight and the security of data should be considered when designing them [4].

In summary, our vision on 6G application scenarios is that it will include the strengthened three 5G scenarios and other new scenarios, as shown in Figure 5. For the strengthened three 5G scenarios, i.e., feMBB, umMTC, muRLLC, MBRLLC, and ERLLC scenarios, the performance will be largely improved in 6G. A variety of potential new application scenarios are also enabled by 6G, such as (1) human-centric services, (2) long-distance and high-mobility communications, (3) extremely low-power communications, (4) convergence of communications, computing, control, localization, and sensing, (5) space-air-ground-sea integrated networks, (6) distributed AI (e.g., federated learning) applications, (7) remote holographic unmanned systems, (8) Internet of bio-nano-things, to name a few [12]. A comparison of 5G and 6G requirements of key performance metrics and application scenarios is illustrated in Figure 6.

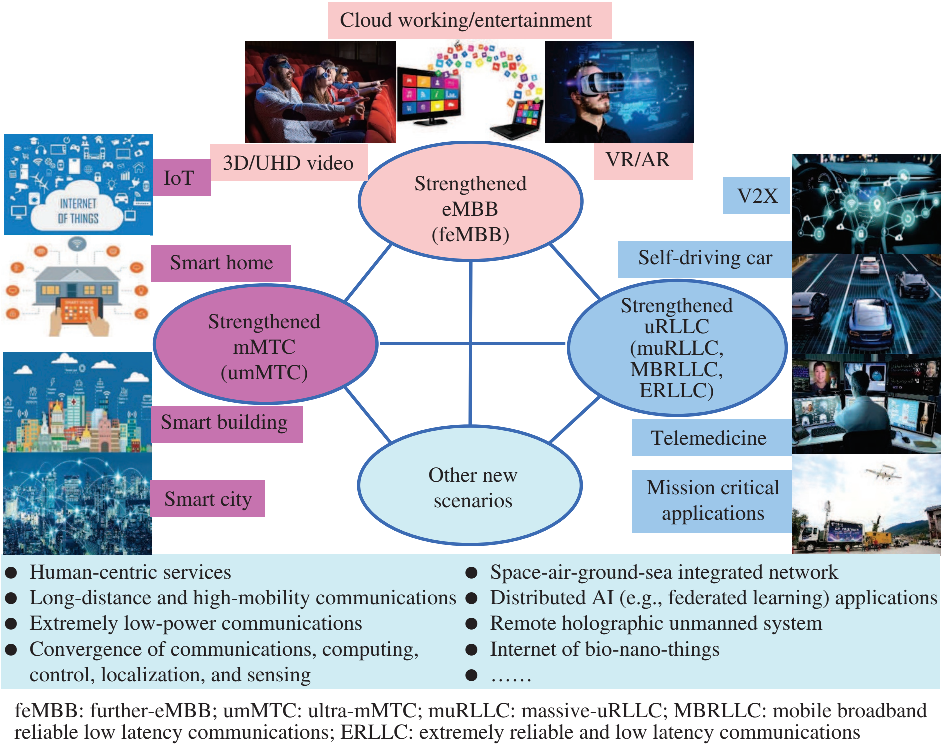

**Figure 5** (Color online) Application scenarios of 6G communication networks.

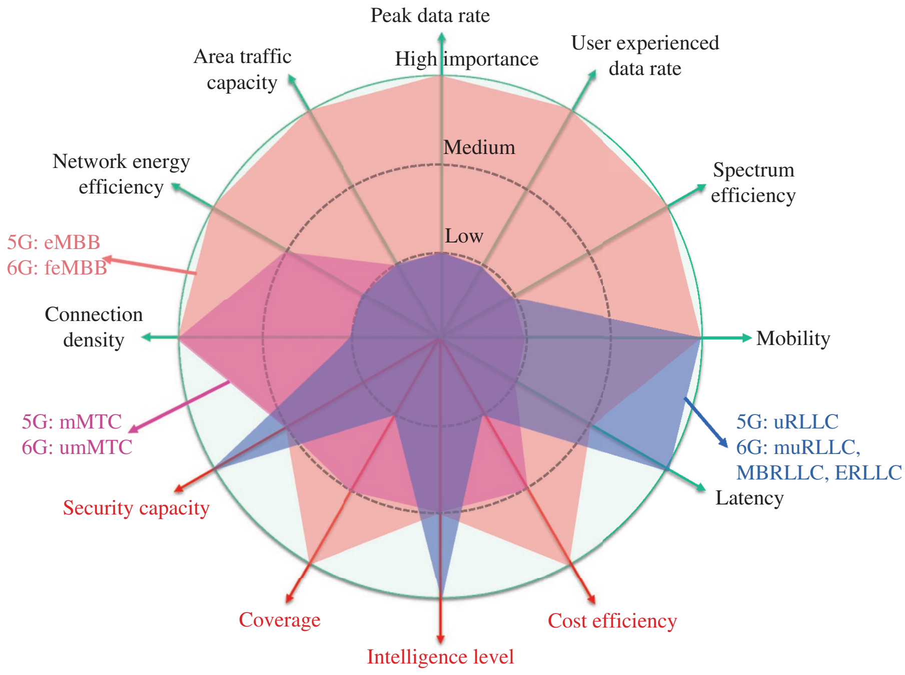

**Figure 6** (Color online) Comparison of 5G and 6G requirements of key performance metrics and application scenarios.

### 2.2 Example industry verticals

The current 5G wireless communication networks have shown the possibility to be the fundamental infrastructure of modern information society. There have been some potential industry verticals based on 5G networks and it will be deepened in future 6G networks. Here we emphasize on example industry verticals, including cloud VR, IoT industry automation, C-V2X, digital twin body area network, and energy efficient wireless network control and federated learning systems.

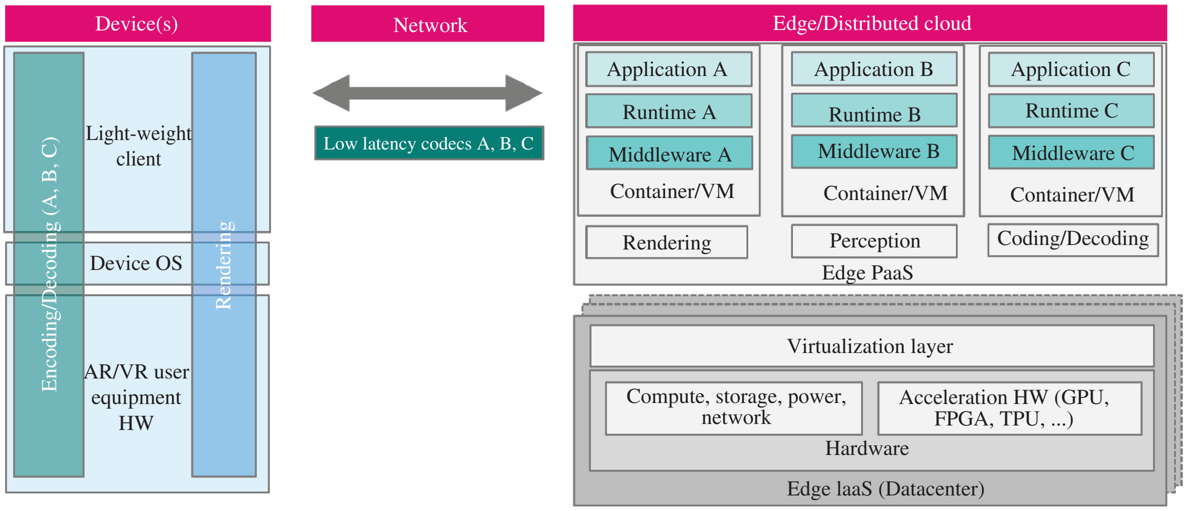

**Figure 7** (Color online) Cloud VR reference architecture [17].

##### 2.2.1 *Cloud VR*

VR technology has been employed in several professional verticals such as education, healthcare, and military [13–16] over the last few years. Because VR provides great immersive and interactive experiences by breaking through cost and risk constraints in the physical world, it has significant potential to reshape our daily life and work eventually. Because VR is a computer-generated world, computing power is one of the key requirements for immersive experiences. Mobile VR is envisioned to be the future owing to its lower price, compact size and portability. However, the processing capability of the mobile VR devices cannot meet the high-fidelity VR requirements. This inevitably leads to unsatisfactory QoE. An ideal VR system must satisfy all the three requirements at the same time: cheap, high-quality graphic, and portability.

Thanks to the advancement of 5G and multi-access edge computing (MAEC) technologies, the idea of cloud VR has been turned into an approachable target. Figure 7 presents a cloud VR reference architecture [17]. We started to see cloud VR services offered by telecommunication operators since 2018, such as China Mobile[1)], LG Uplus[2)] and China Telecom[3)]. The popularity of cloud VR may be attributed to several benefits to customers, developers, and operators. For customers, cloud VR enables them to enjoy high-quality VR service experience anytime and anywhere without buying expensive terminals. For developers, cloud VR allows them to gain more revenue by reaching out to massive customers and reduce porting costs. For operators, cloud VR can be a prime use case to leverage the use of 5G and MAEC. Most importantly, it can help operators to embrace the digital business transformation, from the traffic operation to experience operation, from connectivity to cloud platform and services.

Despite small scale solutions already exist today, there are several crucial challenges which should be improved towards 6G era. We summarize the most important aspect as follows. Firstly, cloud VR platforms require the usage of mobile edge computing (MEC) with expensive accelerators which lead to high investment cost. Secondly, the cloud VR services require extremely low latency which is a key challenge for better customer experience. Last but not least, the cloud VR architecture allows the research community to optimize the processing pipeline. To solve the problems, a thorough understanding and exploration of existing academic and industrial research and development can help to improve the cloud VR system towards 6G.

A set of graphics processing units should provide rendering services to run multiple VR applications simultaneously for different customers in a commercial deployment scenario. In order to save the investment cost, it must bring together many technologies to function. For general purpose, foveated rendering is employed to improve the rendering performance. In cloud VR, the foveated rendering is helped to

1) China mobile launches cloud-based VR service with Huawei. 2018. https://www.telecomtv.com/content/vr-ar/huawei-helps-china-mobile-fujian-release-worlds-first-operator-cloud-vr-31764/.
2) LG Uplus to launch 5G-based cloud VR game service. http://www.koreatimes.co.kr/www/tech/2019/07/133_271632.html.
3) China Telecom Sichuan powers cloud VR with dual-gigabit services. https://www.huawei.com/en/about-huawei/publications/communicate/88/china-telecom-sichuan-cloud-vr-dual-gigabit.

reduce the computational loads in the cloud and lower the transmission bandwidth. Authors in [18] introduced the method to lower the amount of shading by using eye-tracking. The key contribution of their study is to demonstrate how the presence of foveated rendering can improve the rendering performance by reducing the field of view. Authors in [19] presented a cloud VR system based on the simulator platform SIGVerse that collects human gaze-controls and interpersonal-behaviors by using immersive interfaces. Their experiments reveal the effectiveness of the cloud VR system with foveated rendering. Authors in [20] conducted experimental evaluations of DeepFovea, an AI-assisted foveated rendering process and achieved more than 14 times compression on RGB video with no significant degradation in user-perceived quality. Their results indicate that deep learning (DL) based foveation would significantly reduce the rendering load.

To provide an ultimate immersive experience, reducing latency is one of the biggest technical challenges of a cloud VR system. After the frames are computed on edge cloud servers, they have to be captured and encoded before being transmitted over the downlink 5G network. Low-latency video compression schemes are introduced to improve the efficiency and customer experience in cloud VR. Authors in [21] presented an optimized codec to accelerate the encoding process. They develop the methodology by introducing some rendering information such as the camera location, object location, and the distance between the camera and object to reduce the encoding complexity. The experimental results demonstrate that it is possible to save 42% of encoding time without degrading the graphic quality. Authors in [22] explored the approach to interpolate the frames by introducing motion estimation. They use image homography technique for better motion prediction and introduce a dedicated interpolation algorithm for accurate interpolation. The experimental results reported that they achieve 18% speedups on the whole encoding process.

From local rendering to cloud rendering, there are different rendering pipelines, just as the offline rendering pipelines for film production have undergone different evolutionary paths. The local rendering process consists of four conceptual stages: application, geometry processing, rasterization, and pixel processing. The cloud rendering process consists of 4 more stages: user input capture and transmission, application, geometry processing, rasterization, pixel processing, frame capture and encoding, frame transmission, and frame decoding and processing. The slowest process in the pipeline will be the bottleneck and which sets the limit for the end-to-end system. It is necessary to optimize the performance of the cloud rendering pipeline. Authors in [23] designed a VR framework, which split the foreground interactions and background environment for each frame. This system employs a split rendering architecture running on both the client and the server to reduce the computational loads in server. The experimental results demonstrate the split architecture can satisfy the requirements of latency and performance. Authors in [24] proposed a revised planar map rendering pipeline to improve the performance and reduce the latency.

##### 2.2.2 *IoT industry automation*

Industry 4.0 is the short term for the fourth stage of the industrial revolution. Compared to the third stage of the industrial revolution, which uses electronics and information technology (IT) to achieve manufacturing automation, Industry 4.0 aims at bringing the industrial production with significantly improved flexibility, versatility, usability, and efficiency based on the cyber-physical system. As the vision of future smart manufacturing, Industry 4.0 will further introduce information and communication technology (ICT) approach, for instance, integration of the IoT and explore the advantage of ubiquitous connectivity services in industrial manufacturing domain [25]. Owing to the strong focus on machine type communication and ultra-low latency and high-reliability communication, 5G as "one size fits all" solution makes a perfect match with Industry 4.0.

However, owing to the peculiarities of such vertical industry, there are many challenges to be considered as well, for instance, the diversity of use cases with demanding requirements in terms of network performance, challenging propagation environment with potential high interference, consideration of brownfield installations which may require seamless integration, specific safety and security concerns and hurdles of cross-industry terminologies. In order to tackle the above mentioned issues and establish a cross-industry common understanding, the 5G alliance for connected industries and automation (5G-ACIA) was established in April 2018. It is a global forum for addressing, discussing, and evaluating relevant technical, regulatory, and business aspects with respect to 5G for the industrial domain. It reflects the entire ecosystem and all relevant stakeholder groups, ranging from operating industry players (industrial au-

**Table 1** Industrial use cases and requirements [26]

| Use case (high level) | | Availability (%) | Cycle time (ms) | Typical payload size | # of devices | Typical service area |
|---|---|---|---|---|---|---|
| Motion control | Printing machine | >99.9999 | <2 | 20 bytes | >100 | 100 m×100 m×30 m |
| | Machine tool | >99.9999 | <0.5 | 50 bytes | ∼20 | 15 m×15 m×3 m |
| | Packaging machine | >99.9999 | <1 | 40 bytes | ∼50 | 10 m×5 m×3 m |
| Mobile robots | Cooperative motion control | >99.9999 | 1 | 40–250 bytes | 100 | < 1 km$^2$ |
| | Video operated remote control | >99.9999 | 10–100 | 15–150000 bytes | 100 | < 1 km$^2$ |
| Mobile control panels with safety functions | Assembly robots or milling machines | >99.9999 | 4–8 | 40–250 bytes | 4 | 10 m×10 m |
| | Mobile cranes | >99.9999 | 12 | 40–250 bytes | 2 | 40 m×60 m |
| Process automation (process monitoring) | | >99.99 | >50 | Varies | 10000 devices per km$^2$ | |

tomation companies, engineering companies, production system manufacturers, and end-users), the ICT industry (chip manufacturers, network infrastructure vendors, and mobile network operators), academia, and other groups [26]. 5G-ACIA has defined the areas of applications and use cases for automation in manufacturing domains that will utilize 5G technologies.

- Application areas. (1) Factory automation, (2) process automation, (3) human-machine interfaces and production IT, (4) logistics and warehousing, and (5) monitoring and predictive maintenance.
- Typical use cases. (1) Motion control, (2) control-to-control, (3) mobile control panels, (4) mobile robots, (5) massive wireless sensor networks, (6) remote access and maintenance, (7) AR, (8) closed-loop process control, (9) process monitoring, and (10) plant asset management.

One use case may apply to one or more application areas. Such definitions and clarifications are also accepted by the 3rd generation partnership project (3GPP) [27] as the motivation of 5G system design in order to support vertical industries. Other than defining the use cases, it is also important to lay out the specific requirements, especially the challenging ones. Table 1 indicates some typical use cases with clearly stated performance requirements [26]. Among all these, motion control is one of the most challenging closed-loop control use cases in industrial automation. As a motion control system, it is for controlling moving/rotating certain components on machines, which requires ultra-high reliability and low latency, deterministic communication capability, etc. Certain motion control use cases maybe even difficult to be fulfilled by 5G. Hence they could be considered as the initial use cases for 6G.

##### 2.2.3 *C-V2X*

V2X is a key enabler for autonomous vehicles which holds great promises in improving both road safety and traffic efficiency [28]. C-V2X is a standardized V2X solution specified by 3GPP to provide communication capability with low latency, high reliability, and high throughput for vehicle-to-vehicle (V2V), vehicle-to-pedestrian, vehicle-to-infrastructure, and vehicle-to-cloud.

There are two significant versions of C-V2X, namely long term evolution vehicle (LTE-V) and new radio vehicle (NR-V) [29, 30]. LTE-V is developed based on the long term evolution (LTE) system and primarily provides key information delivery and exchange for basic road safety. NR-V is based on the 5G NR system and enhanced to support more stringent requirements on latency, reliability and throughput for advanced V2X applications, such as platooning, high-level autonomous driving, extended sensor and remote driving. NR-V aims to achieve 3 ms or lower latency, 99.999% reliability and even 1 Gbps data rate for extended sensor information. Key technologies for NR-V to meet these key performance indicator (KPI) requirements include effective wireless resource scheduling and powerful MEC. Many methods have been proposed to optimize resource scheduling on PC5 interface [31–33]. The MEC architecture has been defined well by 3GPP V2X applications and the MEC platform (servers) can be deployed either inside of a cellular network, or at the roadside with a connection to the cellular network. The choice will depend on both the business model and related policies. With the help of MEC, C-V2X has the capability to support cooperative perception, cooperative computing, and cooperative decision for autonomous vehicles. The computing task offloading strategies have been investigated extensively for distributed sensor data computing.

With the accelerated permeation of AI, autonomous functionalities and the intelligence level of vehicles will surely progress. The next-generation C-V2X should provide a systemic solution to support the

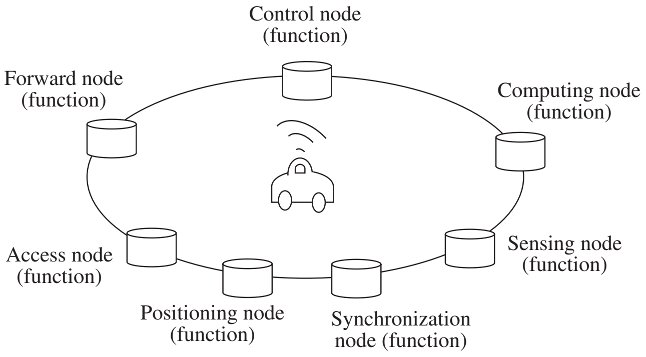

**Figure 8** ICDT-based C-V2X architecture.

cooperation among the key players in an intelligent transportation system (ITS), and should extend its fundamental capability from information delivery to end-to-end information processing, including information acquisition, delivery, computing, storage, security, sharing, and utilization. Some technology challenges and solutions have been reported. Moreover, the following issues need to be further addressed.

**(1) ICDT-based C-V2X architecture and air interface.** An information, communication, and data technology (ICDT) based C-V2X architecture is the cellular network architecture with the full virtualized network resources (communication resources, computing resources, and storage resources) which can be flexibly scheduled for C-V2X communication, computing, and data storage. All the C-V2X functionalities inside of network are dynamically generated according to driving demands.

Figure 8 shows the scope of C-V2X functionalities with the multiple-level distributed architecture. The architecture points out the integration trend of cloud, network, edge, and mobile equipment. The network resources, data, and capabilities are controlled globally for C-V2X applications. Both the control nodes and computing nodes include central nodes and distributed nodes.

The access nodes, i.e., base stations (BSs) or roadside units, are responsible for network access. The future C-V2X architecture should support the deterministic transmission, in additional to the enhanced performance of latency, reliability, and throughput. Synchronization is the premise. To enable cooperative perception for the vehicles equipped with several radars, lidars, cameras, and other sensors, some of access nodes should have multiple functions, namely positioning, sensing, and synchronization. High-accuracy positioning is a key issue for C-V2X. All these capabilities should be exposed for V2X applications.

The future C-V2X may introduce massive MIMO with mmWave bands, visible light communications (VLC), and THz communications to support cooperation among ITS players. These new technologies not only provide enhanced transmission performance, but also provide positioning, sensing, and 3D imaging capabilities. So, the future C-V2X air interface should be designed with a unified wireless waveform for the simplicity of purpose.

**(2) C-V2X technical verifications and testing.** A lot of test studies for C-V2X have been carried out all over the world. But it is still a difficult work to test the C-V2X functionalities and performance for the typical V2X use cases in the field testing scenarios [34]. The digital twin provides virtual representations of systems along their lifecycle and should be developed to a good method for C-V2X based autonomous driving test. By using the digital twin mapping, the real automatic driving vehicle test under the virtual complex road environment can be realized based on the virtual simulation test environment. Actually, an autonomous vehicle can build a virtual driving environment as the digital twin of real road environment by real time cooperative perception based on cloud computing and edge computing. All the decisions for driving behaviors are made from the digital twin environment.

**(3) New spectrum for C-V2X.** The spectrum requirements of C-V2X sidelink communications for autonomous driving are studied in [35]. In a NR-V system, the broadcast mode is for the status information and environmental information delivery and the groupcast mode is for negotiation and decision messages in autonomous driving. The spectrum requirement for broadcast maybe at least 30–40 MHz, while the requirement for groupcast can be neglected. As mentioned above, massive MIMO with mmWave bands, VLC, and THz communications introduce abundant new spectrum resources for C-V2X. The spectrum requirement evaluations with a reasonable methodology and assumptions for these potential C-V2X technologies need further work.

##### 2.2.4 *Digital twin body area network*

In 2016–2018, Gartner listed digital twin as the top ten strategic technology development trends for three consecutive years and believed that digital twin would produce disruptive innovation. Multinational enterprises such as Siemens, Dassault, PCT, and ESI all arrange digital twins. Dassault, Hisense, and other companies try to realize the dynamic monitoring and simulation of human body operation mechanism and medical equipment, accelerate drug research and development, epidemic prediction and tracking, etc.

In 2019, the first 6G summit also proposed that "devices connect senses and motion control, and the digital world and the physical world are intertwined", and devices and surrounding network infrastructure monitor health status [36].

Under the current network conditions, digital technology is mainly used in the monitoring of macro-physical indicators and the prevention of dominant diseases. The real-time and accuracy need to be further improved. With the arrival of 6G technology and the further maturity of interdisciplinary subjects such as bioscience, materials science, and bioelectronics medicine, it is expected to realize a complete "digital twin of the human body" in the future, that is, through the extensive application of a large number of intelligent sensors (>100 devices/person) in the human body, to accurately carry out important organs, nervous system, respiratory system, urinary system, musculoskeletal, emotional state, etc. The real-time "image mapping" forms an accurate replica of the virtual world of the whole the human body, and then realizes the real-time monitoring of the personalized health data of the human body. In addition, combined with the results of magnetic resonance imaging, computed tomography (CT), color ultrasound, blood routine, urine biochemistry, and other professional imaging and biochemical examination, the use of AI technology can provide accurate assessment and timely intervention of individual health status, and can provide an important reference for the next step of accurate diagnosis and personalized manual program of professional medical institutions [37].

Digital twin body area network can simulate virtual human body through 6G and ICT; can track all-weather, predict disease in advance; can also simulate operation and medication for virtual human body, predict effect with virtual human, accelerate drug research and development, reduce cost, so as to improve human life quality.

The digital twin body area network is powerful in the management of epidemic by cloud layer, fog layer, and sensor layer computing, as illustrated in Figure 9 [38]. Take the coronavirus (COVID-19) epidemic management as an example, the following processes will be experienced.

(1) A digital twin network that transmits health information to the personal network slice of edge computing in real-time.

(2) Through the depersonalization model of secure multi-party computing, the anomaly is found by comparing the deployment of fog computing.

(3) The channel resources of the Internet of heaven and earth are allocated to the remote cloud computing center through the independent gateway.

(4) Doctors find the source of infection by tracking the source of crowd behavior through non-contact detection of digital twins.

(5) Simulate survey and verify clinical drugs through the digital twin to speed up the data process.

(6) In the process of isolation, emotional interaction between patients and their families is carried out through synesthesia network.

There is a strong connection between traditional mobile communications and micro-mobile communications in body, body surface, and body domain, as illustrated in Figure 10 [39]. The network architecture features of digital twin body area network include the following.

(1) Small and flexible. In the 5G era, edge computing and network slicing take enterprises or parks as the smallest unit, and in the 6G era, will be even smaller to individuals.

(2) Fog computing and intelligent collaboration. Intelligent selection and collaboration of multiple networks promote network management autonomy.

(3) Open AI crowdsourcing mode. On the basis of SDN before 5G era, digital twins are developed, the proportion of uplink data is greatly increased, and more fine-grained open AI can realize the data value of enterprises and individuals.

(4) Privacy and security. Secure multi-party computing, blockchain, and privacy data does not come out of the body network, only the model leaves the body network, which not only ensures the security and privacy, but also highlights the value of data and remaining computing, storage, and bandwidth resources.

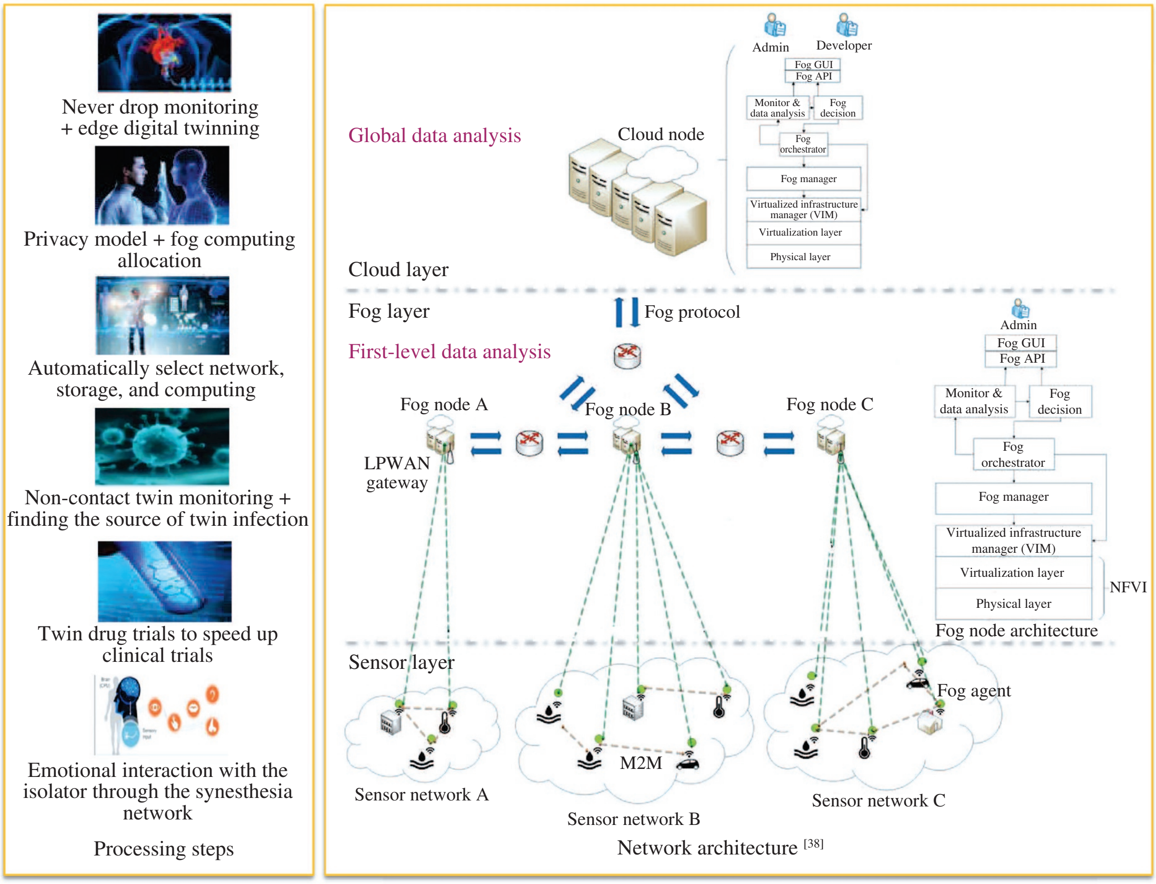

**Figure 9** (Color online) The schematic of the digital twin body area network to help manage the epidemic.

(5) Green and energy. Self-generating equipments such as smart shoe treading power generation and the energy-saving mode of flexible scheduling of computing tasks, solve the last kilometer of the development of wearable agents such as capsule robots in the human body.

#### 2.2.5 *Energy efficient wireless network control and federated learning systems*

The world is moving towards applications that massively use correlation of data for analytics. A new example is the fight of pandemics as COVID-19 with the help of proximity data collected in mobile devices, which then has to be correlated across the network to slow-down the spread of the disease[4]. Also, for automated driving across intersections with a diverse set of participants (bicycles, pedestrians, cars, two-wheelers) it seems important to combine mobile and fixed sensors, e.g., lidar and radar, to learn the situation and control the traffic as needed, in a distributed control approach.

Both simple examples, the mobile terminal enabled big data analytics application, as well as the mentioned tactile Internet application, clearly show that we will require a 6G network to massively support the data traffic required for implementing distributed learning algorithms. Depending on the algorithms split, the latency requirement can be high ($\leqslant 1$ ms) or can be relatively low ($\geqslant 10$ ms). The amount of data that need to be transported, however, will be reaching a new level of magnitude when comparing it to the video and streaming dominated traffic of 5G networks.

The natural approach for preparing 6G networks for these unexplored new traffic requirements is to build on further softwarization of the network functions. This allows to freely adapt to a fairly undefined newly developing requirement. However, when relying on COTS server technology for implementation, this comes at the cost of increase in power consumption. The unprecedented need for compute power will not be served in an energy efficient COTS server, as Moore's law is flattening. That is to say, we cannot rely on semiconductor scaling to compensate the increase in COTS server power consumption.

4) PEPP-PT. www.pepp-pt.org.

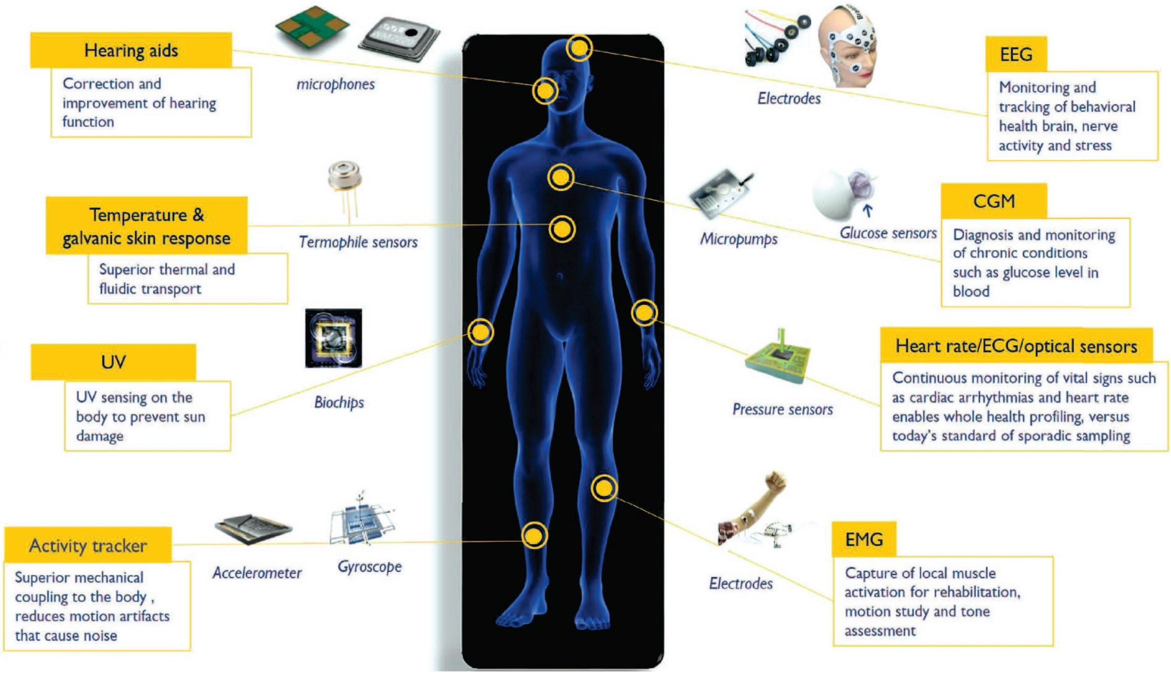

**Figure 10** (Color online) The connection between traditional mobile communications and micro-mobile communications in body, body surface, and body domain [39].

Hence, the only solution foreseeable today is to build domain specific compute engines for 6G which will complement COTS servers and reduce the energy consumption by orders of magnitude. Today we rely on exactly this approach for implementing ML efficiently. For 6G we will have to find an equivalent solution by designing domain specific machines [40, 41] which complement today's COTS servers and AI engines.

### 3 Enabling technologies

In order to achieve the aforementioned system performance metrics, lots of novel enabling technologies will be used in 6G wireless communication networks. In this section, we will first introduce the new air interface and transmission technologies, followed by network architectures of 6G.

#### 3.1 Air interface and transmission technologies

Air interface and transmission technologies provide the connection between the user terminals and 6G wireless communication networks. Here we will focus on several aspects including new waveforms, multiple access, channel coding, CF massive MIMO, dynamic spectrum sharing and accessing, blockchain-based wireless accessing and networking, photonics defined radio, and multi-connectivity (MCo) technique for uRLLC.

##### 3.1.1 *New waveforms*

**(1) Overview of current waveforms.** The waveform is a signal shape in the physical medium formed by a specific method. A flexible waveform needs to consider various parameters, including the time/frequency localization, robustness to time/frequency dispersions, spectral efficiency, latency, and peak to average power ratio (PAPR) [42].

In the development of 5G standard, many kinds of orthogonal frequency-division multiplexing (OFDM) waveform schemes are studied to reduce the out of band radiation. These schemes include the multi-carrier system with sub-band filtering and the multi-carrier system with sub-carrier filtering. The former includes universal filtered multi-carrier and filtered OFDM, and the later includes generalized frequency division multiplexing (GFDM) and filter bank multi-carrier (FBMC). The selection of the new waveform

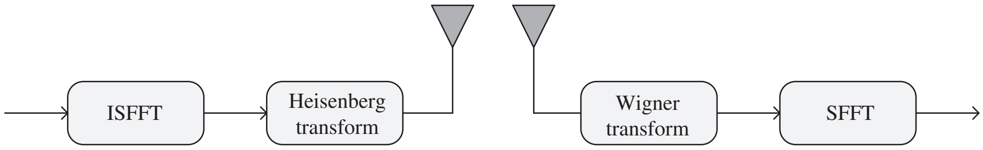

**Figure 11** OTFS Tx and Rx.

needs to consider not only the above performance, but also the frame structure design, the flexibility of parameter selection, the complexity of signal processing algorithm and so on. To flexibly support different applications, multi-service OFDM has been studied. In future systems, the new waveform should also support flexible slicing of the network.

The band above 52.6 GHz is considered as a potential band of 6G systems. Transmitters (Txs) above 52.6 GHz have higher PAPR requirements, because frequencies above 52.6 GHz are faced with more difficult challenges, such as higher phase noise, extreme propagation loss owing to high atmospheric absorption, lower power amplifier efficiency, and strong power spectral density regulatory requirements, compared to lower frequency bands. Therefore, it is necessary to study the waveform suitable for higher frequency bands. Single-carrier system has been proved to be an effective low PAPR transmission method and has been applied to the IEEE 802.11ad standard. With the increase of bandwidth, the flexibility of single-carrier transmission to use large bandwidth is limited. In [42], multi-carrier transmission combined with single-carrier frequency-domain equalization was studied. Its basic idea is to use filter banks to realize a carrier aggregation and single-carrier frequency-domain equalization on each carrier to reduce PAPR and receiver (Rx) complexity.

High-speed mobility is still a limitation of traditional mobile communication systems. With the development of future high-speed train (HST) and the demand of high-speed data transmission, how to improve the transmission rate in high mobility scenario is still a challenge for 6G in the future. A new waveform modulation technique, named as orthogonal time frequency space (OTFS) modulation was studied in [43,44]. OTFS modulates the signal to the delay Doppler domain, and equivalently transforms the time-varying multipath channel to the time-delay-Doppler domain. OTFS can also be viewed as modulating each symbol onto one of a set of two-dimensional (2D) orthogonal basis functions, which was specifically designed to combine the time-varying multipath channels.

The Tx and Rx of OTFS are shown in Figure 11. The transmitted time domain signal in OTFS can be obtained by first applying the 2D inverse symplectic finite Fourier transform (ISFFT) to baseband (BB) data $x(n)$, followed by Heisenberg transform. At the Rx, the received signal $r$ is de-vectorized into a matrix, and then followed by a Wigner transform as well as a symplectic finite Fourier transform (SFFT). OTFS with advanced Rx can achieve a much better performance than traditional OFDM, especially in the high mobility scenario. However, OTFS also has many unsolved issues. The channel estimation and pilot design are more challenging than OFDM [45], and the equalization becomes more complex than traditional OFDM [43]. The combination of OTFS with high-order MIMO should also be studied in the future.

**(2) Extreme low-power waveform design.** Current and novel mainstream waveforms, e.g., (windowed) OFDM, GFDM, or OTFS, are being used or proposed in combination with high modulation cardinality (e.g., 256 quadrature amplitude modulation (QAM)) and with multiple antennas. These waveform technologies have been driven by maximizing the spectral efficiency. The approach is following the correct optimization target for carrier frequencies below the mmWave band.

At the Rx side these waveform technologies require for analog-to-digital (A/D) converters with a resolution of typically 10 bits and beyond. A data rate in the order of 100 Gbps to 1 Tbps quickly leads to a circuit whose A/D converters alone exceed 10 W power consumption [46] within terminals. The result is that the power consumption of terminal transceivers will become dominated by the A/D converters.

However, spectral frequency bands which are being discussed for 6G data rates are to be reaching beyond 70 GHz, possibly into the THz region. At these frequencies the available bandwidth is not scarce. Hence, even when needing to support data rates of 100 Gbps and beyond, a multi-beam link requires less than 5 bit/s/Hz spectral efficiency per beam.

This new boundary condition allows to leave the path of designing more and more spectrally efficient but energy inefficient multi-carrier modulation schemes. Instead, modulation schemes can be designed around optimizing the power consumption of the underlying assumptions on the A/D converter.

The goal of dimensioning an A/D converter is to produce a number of conversion steps per second which must be a multiple of the data rate. At current and future nano-scale semiconductor technologies the voltage swing is very low, but achievable time resolution is high. Achieving the highest figure-of-merit of an A/D converter, i.e., the minimum energy per conversion step is given at 1-bit A/D converters, at the required number of conversion steps per second.

Therefore, a new design paradigm for 6G waveforms could be to find modulation schemes which require very low-resolution A/D converters, the minimum being 1-bit A/D converters. One approach can be the novel waveform design "zero-crossing modulation" [47]. Another approach could be to use continuous phase modulation (CPM) [48]. Further approaches are clearly going to be proposed, as the pressing need for a waveform solution which allows for the terminal's A/D converter power budget to be clearly below 1 W is imminent.

##### 3.1.2 *Multiple access*

Multiple access techniques define how bandwidth resources are shared among mobile users and have been recognized as key milestones for the migration of cellular networks [49, 50]. For the past generations of mobile networks, orthogonal multiple access (OMA) has been used, where orthogonal bandwidth resource blocks are first generated in the time, frequency, or code domains (e.g., time slots, sub-carriers, and spreading codes), and then allocated to users in an orthogonal manner, i.e., one resource block is solely occupied by a single user. The success of OMA in the past-generation mobile networks is mainly due to the fact that it can be implemented in a low complexity way, although it has been known since Shannon's work on multiple access channels that the spectrum efficiency of OMA is sub-optimal [51]. Non-orthogonal multiple access (NOMA) is a paradigm shift for the design of next-generation multiple access techniques. The key idea of NOMA is to encourage the spectrum sharing among mobile users, where the superior spectral efficiency gain of NOMA over OMA is obtained by opportunistically utilizing the users' dynamic channel conditions or heterogeneous QoS requirements [52]. Take the power-domain NOMA as an example [53]. Multiple users with different channel conditions are served at the same time, frequency, and spreading code. As such, NOMA avoids spectrally inefficient situation in which valuable bandwidth blocks are solely occupied by users whose channel conditions are poor. Sophisticated multi-user detection techniques are used by NOMA to ensure that multi-access interference owing to spectrum sharing is effectively suppressed with reasonable computational complexity.

NOMA was originally developed for 5G mobile systems, where its superior spectral efficiency has been demonstrated by extensive theoretic studies as well as experimental trials carried out by academia and industry. However, the vision that NOMA should replace OFDM in 5G systems was not fulfilled. Instead, NOMA was used as an optional transmission model for downlink transmission. We note that this setback for the NOMA standardization progress is not because there were no enough interests in NOMA. Despite of the huge interests from industry and academia, there are still some technical reasons and arguments which cause the difficulty for the standardization of NOMA. Take multi-user superposition transmission (MUST) as an example, which is a study item in 3GPP Release 14 to use NOMA for downlink transmission [54]. There were 15 proposals by different industrial companies. Such divergence resulted in a compromised situation, where MUST was included in 3GPP Release 15 as an optional mode only [55]. Another example is NOMA, which is a study item in 3GPP Release 16 to use NOMA for uplink transmission. With more than 20 different forms proposed to NOMA, the 3GPP working group could not reach consensus, which resulted in the fact that NOMA was not included in 5G NR [56].

Therefore, an important lesson from the standardization of 5G-NOMA is to avoid divergence, which means that the convergence is the top priority to develop a unified framework of 6G-NOMA.

- From the technical perspective. Such a unified NOMA framework should retain the superior performance promised by different 5G-NOMA forms, e.g., supporting massive connectivity, realizing excellent user fairness, and striking a balanced tradeoff between energy and spectral efficiency. It is worth pointing out that most existing 5G-NOMA forms have been developed for particular purposes. For example, sparse code multiple access (SCMA) was developed primarily to support massive connectivity for mMTC, whereas power-domain NOMA is well known for its ability to increase the system throughput for eMBB [57]. Therefore, a general framework is crucial for the deployment of NOMA in 6G systems, where sophisticated tools, such as multi-objective optimization and multi-task learning, will be useful to establish such a framework.

• From the practical implementation perspective. The unified NOMA framework needs to be robust against dynamic wireless propagation environments and users' complex mobility profiles. To realize a robust NOMA transmission, instead of relying on users' instantaneous channel conditions which can be rapidly changing in practice, the use of users' heterogenous QoS requirements could be more reliable in practice [58], because users' QoS requirements are changing in a much slow manner. In particular, users with low QoS requirements can be grouped with users with demanding QoS requirements for spectrum sharing, which can significantly improve spectral efficiency. When there are users with heterogenous mobility profiles in the system, the use of the delay-Doppler plane has been shown to yield more degrees of freedom for the system design, compared to the use of the conventional time-frequency plane [59, 60].

• From the standardization perspective. The unified NOMA framework should be general enough to represent the key features of different industrial NOMA forms based on spreading, power-difference and scrambling, and can also be adapted to emerging communication scenarios and user cases [61]. Take MEC as an example, which is a new type of services provided to mobile users [62]. Initial studies have shown that the use of the NOMA principle can reduce the delay and energy consumption of MEC offloading, and therefore it is important to ensure that 6G-NOMA can effectively support the MEC functionality [63,64]. Another example is massive multiple access, which is to realize the vision that massive IoT will be supported in 6G. The key challenge for massive multiple access is how to connect a huge number of devices featured by short-packet transmission [65]. Initial studies have shown that the use of NOMA can support semi-grant-free transmission, which can reduce handshaking signaling compared to grant-based transmission and also avoid the QoS guaranteeing issues compared to random access protocols [66].

##### 3.1.3 *Channel coding*

The development of channel coding was inspired by Shannon's pioneering work [67], predicting that an infinitesimally low bit error rate (BER) can be achieved with the aid of channel coding upon attaching redundant information to the transmitted messages. Over the past 70 years since Shannons legendary contribution, numerous forward error correction (FEC) codes have been proposed, which can be broadly classified into block codes and convolution codes.

The 5G standards specify three rather different modes of operation, namely the eMBB mode, the uRLLC mode conceived for diverse mission-critical scenarios and finally, mMTC for industrial and IoT applications. Naturally, these three modes have rather different specifications, in particular in terms of the tolerable delay, which is extremely low for the uRLLC mode, which was predominantly designed for low-latency control applications, such as channel quality signaling and scheduling information. Turbo codes, as well as low-density parity-check code (LDPC) and polar codes [68] were compared [69] in terms of their error correction capability, flexible code-rate and codeword length reconfiguration, their ability to support hybrid automatic repeat request (HARQ), their complexity and numerous other considerations [70]. Ultimately, LDPCs were chosen for protecting the eMBB data channels, because they are quite flexible in terms of their code rates, code lengths, and decoding delay, whilst conveniently supporting HARQ [70]. By contrast, polar codes have found favor for the low-delay control channels, as discussed in [70, 71] in the light of a rich set of performance results recorded for diverse codeword length. Further design lessons, performance results, and the extension of polar codes to quantum coding can be found in [72].

**(1) Next-generation polar codes.** Polar codes have been adopted as the control channel coding scheme in 5G, but it is promising to further explore their potential also in the context of the delay-tolerant data channels in a detailed performance vs. complexity comparison. To elaborate a little further, it has been shown in [73] that they exhibit no error floor under successive cancellation (SC) based decoding, which provides the theoretic basis for the application of long polar codes in mobile communications. As a further inspiration, the simulation results of [74] have demonstrated that the BER performance of polar codes relying on cyclic redundancy check (CRC) aided successive cancellation lists is better than that of LDPC using similar parameters.

The efficient combination of polar codes with high-order modulation schemes — including multi-level polar coded modulation [68] and bit-interleaved polar coded modulation [75] deserves further attention with the goal of improving the attainable throughput. Polar codes have the capability of conveniently adjusting the code rate with the fine granularity of $1/N$. By contrast, the granularity of turbo-coded and LDPC coded multi-level modulation is significantly less flexible than that of polar codes. During the code rate allocation of polar coded modulation, only the reliability of the polarization channel should be considered and the polarization channels having high reliability should be chosen for the transmission of

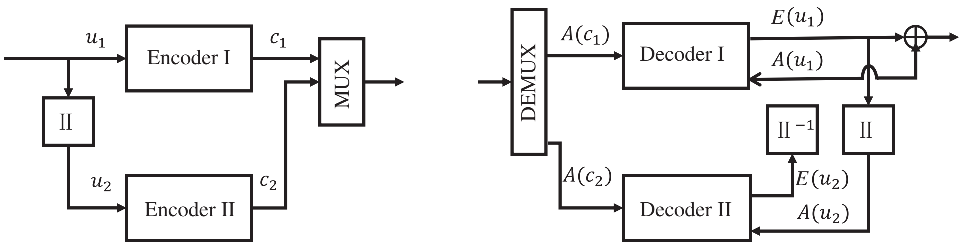

**Figure 12** Parallel concatenated encoder and decoder [93].

the original information bits. However, the complexity of computing the polarization channel reliability is rather excessive [68, 76]. Hence, designing low-complexity code word constructions is a pivotal research issue. Furthermore, optimally mapping the polar encoder's output to the modulator's input is also a challenging task, because the number of legitimate mapping schemes is huge, as argued in [69, 70]. Hence finding near-optimal low-complexity mapping schemes is a promising research area.

To elaborate a little further on the code construction of polar codes, it must take the specific channel characteristics into account, and it directly affects both the choice, as well as the performance vs. complexity of the decoding algorithm. Hence conceiving beneficial code constructions both for additive white Gaussian noise and fading channels has attracted substantial research interest. The associated polarization channel aspects have been investigated in [71, 72, 77], complemented by a range of low complexity code constructions, but there is a paucity of literature on innovative code constructions conceived for fading channels [78–80].

Even though having flexible code parameters is of salient importance, most of the existing polar codes have a length, which is an integer power of 2 and limits their practical application. Hence, similarly to their older counterparts, typically puncturing and shortening are used for appropriately adjusting both the code length and code rate, which imposes a performance degradation. Hence specific kernel matrices have been used for constructing polar code having flexible length without puncturing and shortening in [81, 82]. In this context, multi-kernel based code construction is a promising future research direction. Finally, conceiving powerful soft-decision aided turbo-style detections schemes exchanging soft extrinsic information between the polar decoder and the soft-output demodulator is critical for designing powerful next-generation transceivers.

Stochastic LDPC and turbo decoder implementations have a rich literature owing to their numerous benefits, primarily owing to their fault-tolerance [83, 84]. Surprisingly, their stochastic polar decoder counterparts have hitherto not attracted much attention apart from [85–90]. Hence this is a promising research area worth investigating.

**(2) Next-generation FEC in the turbo-transceiver era.** Historically, the first iterative detection aided channel decoder was Gallager's LDPC decoder [91], but it was not until the discovery of turbo codes that the full benefits of iterative soft-information exchange between the decoder components became widely acknowledged [92]. In simple conceptual terms, the iterative exchange of soft-information seen at the right of Figure 12 [93] is beneficial, because the decoder components refrain from making a hard-decision until they arrive at a consensus and become sufficiently confident in their decision. More quantitatively, it was shown in [94] that Berrou's turbo code is indeed capable of outperforming a similar-complexity convolutional decoder by about 2 dB at the BER of $10^{-4}$, provided that a sufficiently long turbo interleaver is used.

Since the discovery of Turbo code, a large variety of concatenated coding schemes have been conceived, e.g., several potent concatenated schemes, namely parallel concatenated code (PCC) [92] and serially concatenated code (SCC) [95]. Indeed, there are also powerful hybrid concatenated codes (HCCs) [83, 84, 96] that rely on diverse constituent codes and worth exploring in the future. The above-mentioned turbo principle relying on iterative soft information exchange may be employed for detecting any of the above schemes.

**(3) Parallel concatenated schemes.** The component encoders are typically convolutional encoders, binary Bose-Chaudhuri-Hocquenghem (BCH) codes however have also been used, for example, in [94]. At the encoder, the input information bits are encoded by the first constituent encoder (Encoder I) and an interleaved version of the input information bits is encoded by the second constituent encoder

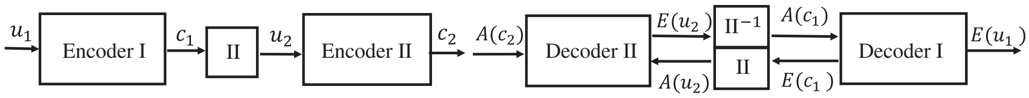

**Figure 13** Serially concatenated encoder and decoder.

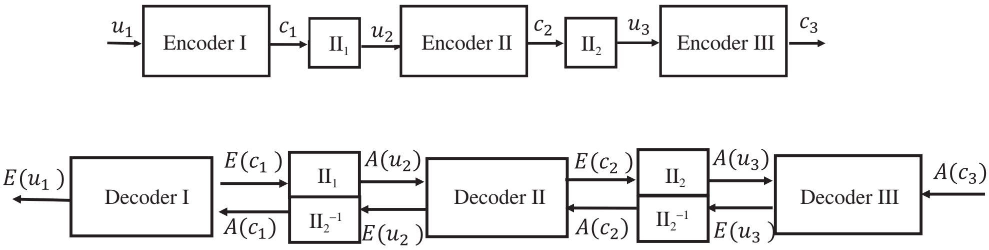

**Figure 14** Three-stage serially concatenated encoder and decoder.

(Encoder II), where represents an interleaver (also termed as a scrambler). The encoded output bits may be punctured, hence arbitrary coding rates may be obtained. Furthermore, this structure can be extended to a parallel concatenation of more than two-component codes, leading to multiple-stage turbo codes [85].

**(4) Multiple-component turbo codes for HARQ.** A particularly beneficial family of PCC is the class of multiple-component turbo codes [86], which rely on unity-rate code components. They are eminently capable of supporting HARQ. Briefly, during the first transmission, no redundancy is assigned, hence the overall code-rate is unity. If the CRC indicates a decoding failure, a differently scrambled version of the same information is transmitted, hence the decoder now has 50% redundancy, and the overall code-rate becomes 1/2. In case of continued CRC failure, a total of $N$ differently interleaved versions of the original information are transmitted, which results in an overall code-rate of $1/N$.

**(5) Serially concatenated schemes.** The basic structure of an SCC [95] is shown in Figure 13. The SCC encoder consists of an outer encoder (Encoder I) and an inner encoder (Encoder II), interconnected by an interleaver. The introduction of the interleaver scrambles the bits before they are passed to the other constituent encoder, which ensures that even if a specific bit has been gravely contaminated by the channel, the chances are that the other constituent decoder is capable of providing more reliable information concerning this bit. This is a practical manifestation of time-diversity. Iterative processing is used in the SCC decoder, and a performance similar to that of a classic PCC may be achieved [95]. In fact, the serial concatenation constitutes quite a general structure and many decoding/detection schemes can be described as serially concatenated structures, such as those used in turbo equalization [87, 94], coded modulation [88,89], turbo multi-user detection [97,98], joint source/channel decoding [99–101], and LDPC decoding [102]. Similarly, a serially concatenated scheme may contain more than two components, as in advocated in [103–106]. Figure 14 shows the schematic of a three-stage SCC [103, 106].

**(6) Irregular FEC: the EXIT-chart-aided design era.** In the early days of FEC design, a criterion was that of maximizing the Hamming distance of the legitimate codewords, which was rather closely related to minimizing the BER even for transmission over Gaussian wireline channels. More explicitly, designers wanted to maximize the Hamming distance between all pairs of legitimate codewords. The family of BCH codes satisfied this design criterion [94]. The Viterbi decoding of convolutional codes relied upon the maximum likelihood sequence estimation (MLSE) algorithm [107], which does not minimize the BER, but the erroneous sequence estimation probability. However, in 1974, Bahl, Cocke, Jelinek, and Raviv (BCJR) had invented another optimal decoding algorithm for the family of linear codes, which acquired the fond co-notation of the BCJR decoder [108]. Although it was capable of directly minimizing the BER, its performance remained similar to that of MLSE, despite its significantly higher complexity. Hence the BCJR decoder remained dormant until the invention of turbo codes, which required an algorithm that provided bit-by-bit confidence metrics for the decoder. After decades of using diverse code design criteria, a historic breakthrough was discovered by Ten Brink in the form of the powerful analytical tool of extrinsic information transfer (EXIT) charts [109–112], which visualizes the convergence behavior of iterative detection aided turbo-receivers.

##### 3.1.4 *CF massive MIMO*

Inter-cell interference is a main limitation to conventional cellular networks. In order to overcome the interference problem, inter-cell cooperation has been studied in LTE under the name of coordinated multi-point (CoMP) transmissions or network MIMO. However, the performance gains of CoMP are still limited under the traditional cellular architecture.

Massive MIMO is one of the key techniques to improve the spectral efficiency for 5G NR. Theoretical results have shown the channel hardening effect of massive MIMO as the antennas increases to infinity. However, considering the correlation between antennas, codebook-based beamforming is usually used to reduce the complexity of multi-user joint precoding in practical implementation [113].

Recently, CF massive MIMO was proposed to overcome the inter-cell interference by breakthrough the cellular architecture [114]. From the point of view of BB transmission, the performance gain of CF massive MIMO comes from the joint processing of a large number of geographically distributed remote antenna units (RAUs) [115, 116]. CF massive MIMO has been shown to achieve higher spectral efficiency than centralized massive MIMO and small cell [117]. Our recent prototyping system in large-scale distributed MIMO has demonstrated that the data rate of 10 Gbps could be achieved by a $128\times128$ large-scale distributed MIMO (or CF massive MIMO) with 100 MHz bandwidth [118]. However, CF massive MIMO is also facing some obstacles such as high implementation complexity, high requirement of backhaul/fronthaul, and difficulty in synchronization and channel state information (CSI) acquisition. At present, researchers have studied the capacity of CF massive MIMO. The results have shown that in the i.i.d. Rayleigh fading channel, when the number of BS goes to infinite, the channels have asymptotic orthogonality. Moreover, when each BS is equipped with an appropriate number of antennas (for example 5–10), CF massive MIMO also has a channel hardening effect [119].

Considering scalability, maximum ratio combining (MRC) reception and maximum ratio transmission (MRT) precoding are usually used in CF massive MIMO. Although they have asymptotic optimality, the results of [120] also show that MRC/MRT cannot fully exploit the spatial multiplexing gain of CF massive MIMO. Using zero forcing (ZF)/minimum mean squared error (MMSE) receiver or ZF/regularized zero-forcing (RZF) precoding can achieve better performance but it would significantly increase system complexity, which is the primary factor restricting the CF massive MIMO's application.

Owing to the need for joint processing, a large backhaul capacity is needed between BSs and the central processing units (CPUs). This is especially true for the joint detection of uplinks, where a large forward link capacity is needed. How to design signal processing methods to minimize the backhaul capacity requirements needs further study.

The exploitation of CF massive MIMO capacity gain depends on the accurate CSI. First of all, precise clock synchronization between BSs is required. One way to solve the synchronization issue is to use hardware, for example, using nodes equipped with high-precision global positioning system (GPS) or nodes using high-precision synchronization protocol, such as IEEE 1588. Another way is to send an over-the-air synchronization signal between nodes. Like massive MIMO, in CF massive MIMO, time division duplex (TDD) mode is usually assumed to reduce overhead. However, in practice, owing to the non-ideal characteristics of radio frequency (RF), reciprocity calibration is needed. Because the nodes of CF massive MIMO are deployed in different locations, reciprocity calibration requires that the over-the-air calibration signals are transmitted among the nodes. Therefore, owing to the influence of inter-node link signal-to-noise ratio (SNR) and the variations of the channel, the accuracy and the frequent of the calibration need to be further studied.

A further important aspect of the CF massive MIMO is the scalability of the power control. The main problem is that while globally optimal policies are known and can be computed in principle (see, e.g., [114] for the max-min fairness policy), these policies do not scale neither in performance nor computationally. For example, it is not known under what exact fading distributions the max-min fairness rate will approach zero when the size of the network increases. Also, the computational load grows very unfavorably with the system size. A remedy is to partition the access points (APs) into clusters and associate each user with one or more such serving clusters [121]. This facilitates the use of local power control mechanisms that can achieve performance well comparable, though naturally inferior, to that of global max-min fairness optimization.

##### 3.1.5 *Dynamic and intelligent spectrum sharing and accessing*

It is a challenging task to support the stringent requirements of 6G on peak data rate and massive access owing to the limited spectrum resources. Although new spectrum bands such as THz and VLC bands are considered for 6G usage to support the peak data rate transmission, it is highly desirable to utilize the low frequency bands, such as sub-6 GHz band, in an efficient manner, for the benefits of broad coverage, low cost, and massive access support. Thus, cognitive radio (CR) techniques [122,123] need to be used to support dynamic and intelligent spectrum sharing and accessing [124,125] in both licensed and unlicensed bands.

• Dynamic spectrum sharing in unlicensed bands. One approach is to operate 6G networks in unlicensed spectrum in addition to the licensed bands. In sub-6 GHz band, the most interest lies in the industrial, scientific, and medical (ISM) bands in 5 GHz spectrum, as there are several hundred MHz of spectrum bandwidths available. However, there are a wide variety of applications such as WiFi, in the ISM bands. Thus, an access protocol needs to be defined for 6G networks to operate in the unlicensed band, similar to LTE unlicensed (LTE-U), or LTE license assisted access (LTE-LAA) [126–128].

• Symbiotic radio (SR). SR is a recent evolution of CR and spectrum sharing. It enables massive access in a spectrum- and energy-efficient manner. Building on ambient backscatter communications [129,130], SR supports the massive access from IoT devices by passively reflecting the signals received from the cellular Txs [131], such as BSs or mobile stations (MSs). Therefore, the MS, BS, or dedicated IoT receiver can collect the massive access data together with the cellular data. It is interesting to note that the IoT transmission shares the same spectrum, and the same infrastructure of the cellular network, and the adding of the IoT transmission can even help the cellular transmission owing to the multipath diversity [132]. Therefore, the new system is called SR network [133].

• AI for dynamic spectrum sharing. The design of spectrum sharing techniques typically requires extensive information exchange among the systems. The network environments in 6G will become more and more dynamic and complex, which imposes difficulty in implementing dynamic spectrum management. AI is becoming an effective enabler to support dynamic spectrum management to tackle these challenges. For example, using deep reinforcement learning (DRL), when operating in the unlicensed band, the LTE network is able to learn the WiFi traffic in order to design a proper protocol to assist fair coexistence [128]. The secondary system in CR network is able to design the modulation and coding scheme by learning the operational pattern of the primary system [133]. In symbiotic radio networks, the user association problem can be solved with limited environmental information using DRL [134].

##### 3.1.6 *Blockchain-based wireless accessing and networking*

Blockchain is a disruptive technology for the IoT. Initially devised for cryptocurrencies (e.g., bitcoin [135]), blockchain has been widely deployed in the areas beyond the financial sector. As the name suggests, blockchain is a chain of informative blocks connected by hash pointers for recording and storing a public shared database. The mathematical property of hash pointers can prevent a confirmed blockchain from tampering because any change to a block will affect its hash value and further influence all child blocks. Usually, blockchain is distributed and it maintains a group of active nodes, known as miners, in a peer-to-peer (P2P) network through a specific consensus process named mining. Currently, blockchain can do more than simple transactions in cryptocurrencies but run Turing-complete programs, namely smart contracts, in a distributed manner, e.g., Ethereum [136]. Smart contracts are verifiable scripts in blockchain and can enforce a variety of digital actions, such as authentication, control, among others.

Being able to establish multifold trust relationships between network entities and unite multilateral groups without any middleman, blockchain provides a promising solution for integrating and sharing various resources across different parties in wireless networks, making it attractive to upcoming 5G and future 6G networks. Indeed, in the 2018 mobile world congress (MWC) and federal communications commission (FCC) outlined their vision of blockchain deployment in future networking for 6G. Recently, a decentralized, trustworthy wireless access paradigm enabled by the blockchain technology, namely blockchain radio access network (B-RAN), was proposed with its prototype [137,138], as shown in Figure 15. B-RAN can build trusted physical links between service providers and clients while supporting, e.g., spectral sharing, cooperative transmissions, multi-hop data brokers, D2D communications, etc. [137,139]. By efficiently pooling and sharing a variety of network resources, blockchain can support and enhance a number of services, such as MEC [140], IoT applications [141,142], energy trading [143], V2X connections [144], and network slicing [145]. Owing to its distributed nature, B-RAN inherently supports federated-style

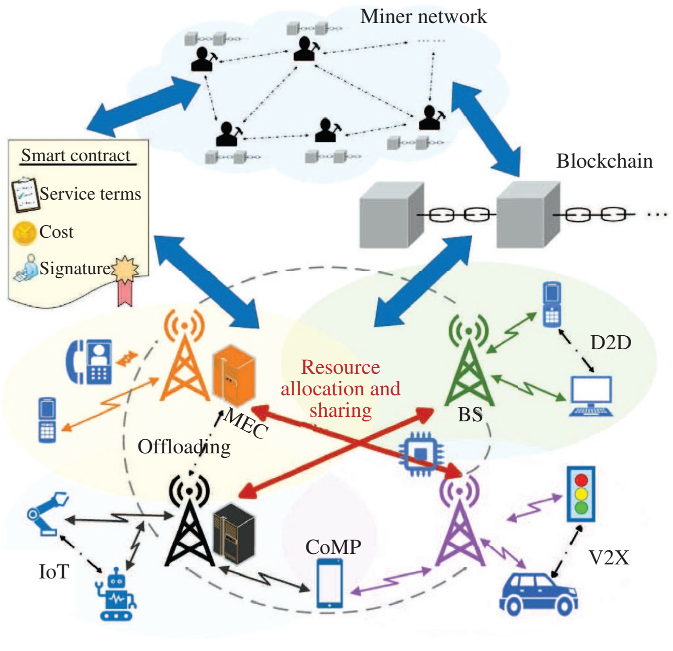

**Figure 15** (Color online) An illustration of blockchain-based networks.

learning at the bleeding edge [146, 147]. In principle, B-RAN plays as a multi-sided platform [147], by leveraging network effects and attracting more participants, to deliver intelligence services to every corner of the network via the integration and coordination of communication, computing, caching, and control units.

Currently, the blockchain-based networking is still in its infancy, with many problems unsolved. First, cryptocurrency blockchains can usually safeguard security but suffer a relatively long latency, also known as the security-latency tradeoff [148]. It still needs more investigations to achieve shorter latency for the delay-sensitive wireless services [149]. Second, current blockchain technologies are facing the scalability problem, which limits the network throughput of blockchain based networks. Studies that analyze and design new blockchain structures may help remove this obstacle [150, 151]. Third, most consensus mechanisms, e.g., proof-of-work, consume a large amount of energy to achieve resistant and robust performance against misbehaviors and are thus unsuitable for mobile devices with limited power and capability [137, 142]. Hence, energy efficient consensus mechanisms are urgently needed in mobile environments.

#### 3.1.7 *Photonics defined radio*

To offer super-broadband mobile services, 5G adopts an NR frequency band above 6 GHz for small cells. One of the typical frequency bands is 28 GHz, for high-speed wireless transmission faster than 10 Gbps. However, owing to limitation of available RF resources, it would be rather difficult to increase the transmission capacity of radio in frequency bands below 100 GHz. The THz band featuring an extremely large bandwidth available between the mmWave and infrared radiation [152, 153] is expected to offer over 100 Gbps to accommodate the exponential increasing wireless data traffic, so they have been recognized as promising candidates for future 6G services [154].

The THz spectral range is not exploited to its full potential owing to the current limitations in sources and detectors. To open the frequency range for applications, photonic solutions have been at the technological forefront. Photonic techniques are desirable solutions for mmWave and THz generation in terms of their energy efficiency, bandwidth, and above all, their tuning range. Methods of generating THz frequencies based on photonic heterodyne mixing techniques can not only overcome the bandwidth limitation of electrical components but also effectively promote the seamless integration of fiber and wireless networks [155], for example, THz-over-fiber (ToF) or fiber-to-the-antenna (FTTA) architectures. Thus, the advantages of the huge bandwidth of fiber communication and high mobility of wireless communication make the fiber-THz-fiber seamless communication system a promising choice [156, 157].

At the THz Tx, the optoelectronic conversion of data signals from the optical to the THz can offer a

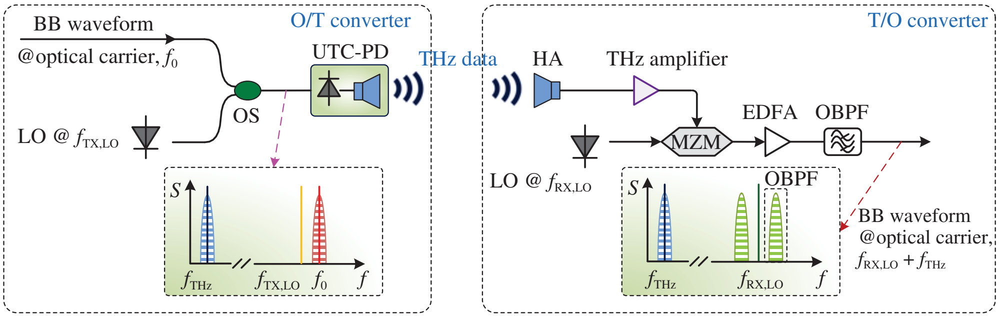

**Figure 16** (Color online) Concepts of direct O/T and T/O conversion.

variety of advantages over conventional all-electronic approaches, including wideband tunability of the THz carrier frequency, and the opportunity to utilize advanced optical modulation and multiplexing mechanism for the generation of multi-gigabits data streams before conversion to the THz domain. As shown in Figure 16, the BB waveform is first modulated onto an optical carrier at frequency $f_0$ and sent to the local optical to THz (O/T) converter. The optical signal is then translated to a THz carrier by photo-mixing with a local oscillator (LO) laser tone at a frequency $f_{\mathrm{Tx,LO}}$ in an ultra-fast uni-traveling-carrier photodiode (UTC-PD). The THz data signal is radiated into the free space by an antenna. At the Rx, the THz signal is converted to the optical domain by means of an ultra-broadband optical modulator, thus leading to high-density photonic integration without all-electronic down-conversion to the BB or to an intermediate frequency. The THz data signal is received by a horn antenna (HA), amplified by a THz amplifier, and then fed to a Mach-Zehnder modulator (MZM) for modulation onto an optical carrier at $f_{\mathrm{Rx,LO}}$. After modulation, the optical signal contains an upper and a lower modulation sideband. An optical bandpass filter (BPF) is used to suppress the carrier and to select one of the modulation sidebands. It is expected that the combination of direct O/T and T/O conversion in ultra-compact devices has the potential to greatly accelerate THz communications and to advance the integration of THz wireless links into fiber-optic infrastructures.

The full deployment of the THz wireless communication technology is facing multiple challenges. While photonics technologies can help in terms of link efficiency, generating high data rates, the system still needs more output power at the Tx, in particular for applications such as backhaul, where the distance will have to reach 1 km [158]. Another challenge is related to the energy consumption, as the wireless data traffic will soon be the highest consumption of energy per habitant in the world [159]. Such challenges will still require developments of technologies to generate more power at the Tx while increasing the overall system efficiency. The primary technology that could improve both the power at the Tx and the overall efficiency of the system is "photonic integration". Photonic integration will naturally reduce coupling losses, such as the loss from fiber to chip and in particular the loss between the laser and the photo mixer [160]. It should also enable the use of multiple antenna systems that would lead to advanced active array antennas to compensate for the path loss and allow for some tracking. However, even with such developments, there is still a clear need for amplification at both the emitter and Rx, thus low-noise and wide-bandwidth THz amplifiers for both Tx and Rx are also at key priority [161]. Application of advanced signal processing techniques, such as the compressed sensing, multi-antenna precoding, and others is also required in order to develop an effective medium access control.

In addition, the graphene-based technology for THz technologies is also a promising area of development, in particular for enhanced detection and emission using graphene-based field-effect transistors, and modulators in graphene-based metamaterials [162, 163]. Although the current performances are not at the state of the art compared with photonics-only-based technologies, the physical properties of graphene are indeed encouraging. In particular, the development of room temperature detectors would be interesting to enhance the detection sensitivity of the system. Finally, every radio wave at frequencies <3 THz should be regulated, and a global consensus for its use for passive and active services needs to be reached.

#### 3.1.8 *MCo for uRLLC*

In 5G, the uRLLC is designed to support the mission-critical applications, such as autonomous vehicles and industrial Internet, which require a high level of reliability and low latency. According to the 3GPP, the main objective of the uRLLC is to minimize the latency down to 1 ms while ensuring packet error rates of less than 99.999%. As is known, in 4G, reliability is obtained by the HARQ procedure. However, the strict latency constraint in the uRLLC does not endorse multiple retransmissions. Fortunately, researchers in industry and academia have found several new approaches to solve this conflicting problem and proposed some advanced techniques in 5G. In order to increase the probability of successful access, the grant-free technique has been standardized where the user equipment (UE) can initiate a transmission without sending any service request to acquire a dedicated scheduling grant. In addition, the uRLLC can occupy the resources which are already assigned to other types of applications by introducing a preemptive scheduling mode, once it has data to transmit. The other related techniques include mini-slot, control resource set, and packet duplicate [164].

Naturally, the uRLLC in future 6G is expected to have better ability than its 5G version. This is driven by the factor that the mission-critical applications, such as robotics and autonomous systems, hunger for higher reliability and lower latency, as described in Subsection 2.2.2. Let us take smart factory as an example. Increasing the reliability of communications is one of the most critical and challenging tasks for massive production. Reliability also plays an important role in multi-factory production, because the failure of one entity may induce a vigorous chain reaction to the others. If the end-to-end reliability and latency can reach 99.99999% and 0.1 ms in future 6G, the productivity and products quality are deemed to be improved significantly. On the other hand, some upcoming new applications will blur the boundary between the eMBB and the uRLLC. For example, it is widely accepted that as a full immersive technique, extended reality (XR), will emerge in many commercial services in the future. XR is expected to encompass augmented mixed, and VR technique can capture all the perceptual inputs stemming from human senses, cognition, and physiology [3]. However, current 5G cannot support XR owing to its inability to deliver huge data in a very low latency. Combining the eMBB and uRLLC may generate a new application scenario in future 6G, which is characterized by higher reliability, lower latency, and higher data rate.

Because the latency of a communication system is mainly determined by the intrinsic packet length or time slot, the new uRLLC requirements mentioned above pose an unprecedented challenge to the basic principle for 6G's design. This is because the reliability and latency of a communication system are inherently contradictory with each other. Fortunately, the potential solution can be found in recent information-theoretic achievements in finite packet length regime. In 2010, built upon Dobrushin's and Strassen's previous asymptotic results, Polyanskiy et al. [165] provided a unified approach to obtain tight bounds on $R$ (coding rate), as a function of $n$ (packet length) and $P_e$ (packet error probability). The rationale behind this approach is that when $n$ is finite, $R$ becomes a random variable composed of channel capacity and dispersion. Channel dispersion is also a random variable introduced as a rate penalty to characterize the impact of $n$. On the other hand, the probability that the coding rate is smaller than $R$ gives the packet error probability $P_e$. Because the latency constraint can be illustrated equivalently by packet length $n$, this finding paves a possible road to enhance the system performance of the uRLLC from an information-theoretic point of view [166].

One of the promising solutions to support both higher reliability and higher data rate is the application of MCo technique, because it can provide a communication framework to make the diversity via multiple routes to the destination [167]. From the recent information-theoretic achievements on finite packet length, we learn that the MCo transmission can be modelled as a single-input multiple-output (SIMO) system with short packets and its reliability is highly dependent on the number of antennas. In this case, the design principle of 6G's uRLLC turns into the following mathematical problem. For a given data rate $R$ and packet length $n$, how many antennas are needed to support the desired reliability $P_e$? Solving this problem would provide important guidance for designing practical systems, because it would yield minimum implementational cost. In addition, for a given number of antennas, as the variance of information density achieved by a capacity-achieving distribution, the channel dispersion is introduced to show how far the data rate is deviated from the capacity owing to the shortening of packet length. In other words, the key issue to solve this problem is to find out the relationship between the channel dispersion and the antenna number under certain conditions.

Another potential solution to balance the reliability and high data rate with low latency is the distributed antenna system (DAS) technique [168], which is naturally an extension of the MCo. Basically, DAS is implemented through massive separated RAUs and is especially useful for providing quality coverage of a specific area, such as an industrial plant. Similar to the MCo, DAS sends the data to the same

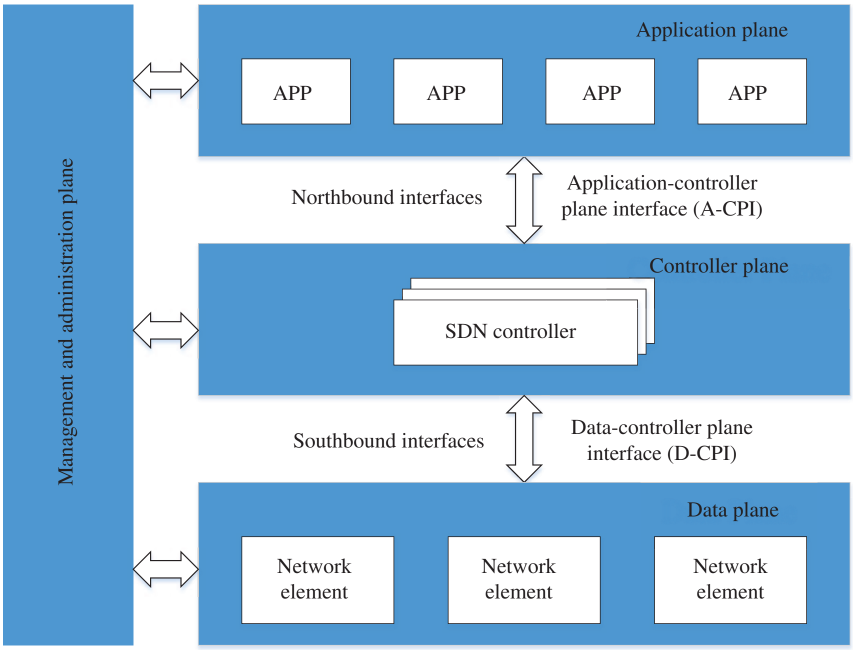

**Figure 17** (Color online) A high-level overview of SDN architecture [171].

destination through multiple radio links, and moreover it can take advantage of flexible balance between reliability and data rate. The reason behind is that the DAS can be treated as a distributed MIMO [168]. Therefore, a tradeoff can be made between diversity and spatial multiplex by exploring the inherent property of MIMO [169]. Supposing that the spatial degree of freedom (DOF) of the distributed MIMO is dof, a full multiplex gain (i.e., highest data rate) can be obtained if the number of dof independent data streams is transmitted to the destination. Alternatively, a higher reliability will be achieved at a cost of a reduced data rate, provided that a number of $d <$ dof independent data streams are sent at the Tx. As a special case, a maximum reliability but with a minimum data rate will be achieved if all the RAUs send an identical data stream to the same destination, which is the same as the SIMO case. More importantly, this approach can be easily extended to multi-user scenarios with the balanced uRLLC performance.

### 3.2 Network architecture

In order to better realize requirements of 6G wireless networks, the novel network architecture should be studied and applied according to network characteristics and specifications. Here we will discuss 6G network architectures in terms of SDN/NFV, network slicing, SBA, CSA, deep edge node and network ($\mathrm{DEN}^2$), CF/cell less, and cloud/fog/edge computing.

#### 3.2.1 *SDN/NFV*

**(1) SDN and its evolution.** SDN is a network architecture that breaks the vertical integration in traditional networks to provide the flexibility to program the network through (logical) centralized network control. It separates the control plane and the data plane, in which the control plane or the control function is logically centralized at one or a set of control entities called SDN controllers, while the data plane is simplified and abstracted for network applications and services requesting through the SDN controllers [170].

From high level, the SDN architecture can be divided into four planes, i.e., data plane, controller plane, application plane, and management and administration plane, as shown in Figure 17 [171]. The data plane consists of distributed network elements that forward and route the data packets to the SDN controllers according to the instructions made in the controller plane via the southbound interfaces, also called data-controller plane interface (D-CPI). The controller plane is composed of one or a set of logically centralized SDN controllers, which are responsible for translating the applications' requests and exerting more granular control over the network elements. It also provides relevant information up to the applications. Thus, the controller plane plays the role of the bridge between the data plane and application

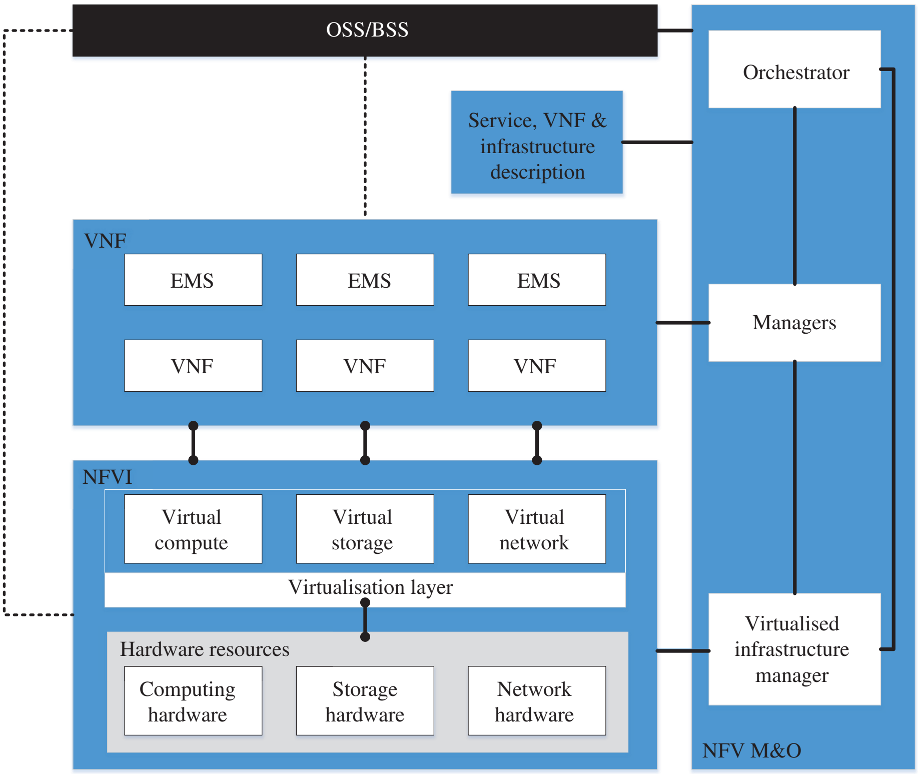

**Figure 18** (Color online) A high-level overview of NFV architecture [175].

plane. The application plane consists of applications and communicates their requirements towards the controller plane via the northbound interfaces, i.e., application-controller plane interface (A-CPI). The management and administration plane was recently proposed by open networking foundation (ONF) and Internet engineering task force (IETF). This plane is responsible for provisioning and monitoring the networks [170–173].

The SDN architecture is programmable, agile, and cost effective, making it a key enabler for 5G. SDN can be used to provide an overall framework to enable 5G to function across a control plane and better data flows for data moving across the 5G network. Many advancements of applying SDN in 5G have been made [174]. However, there are also many problems in terms of deployment, reliability, security and so on. First, deploying SDN in existing networks will incur economic, technical, and organizational challenges. SDN deployments will incur considerable cost in the initialized equipment transformation and professional training. 5G networks will inevitably increase the complexity of SDN design and deployment. Second, to guarantee uRLLC in 5G, a fast and expensive out-of-band wide area network between SDN controllers and switches will be needed, which will pose the huge challenges on the reliability of SDN networks. Third, because SDN controllers are logically centralized and run on Windows or Linux operating systems, the risk of being attacked or failures will also increase. Thus, security is also a fundamental challenge [172].

In the future 6G, the hybrid SDN may be promising to effectively alleviate the above challenges. Hybrid SDN refers to a networking architecture where both centralized and decentralized paradigms coexist and communicate together to different degrees to configure, control, change, and manage network behavior for optimizing network performance and user experience. It is consisted of the traditional network and the pure SDN network, and thus can reap the benefits of both two networks. For hybrid SDN, the effective management of heterogeneous paradigms and the profitable interaction between two networks is of particular importance [172, 173]. Besides, with the introduction of AI to communication networks, the increased automation of SDN networks is expected.

**(2) NFV and its evolution.** NFV is a network architecture where network functions are implemented in software that runs on top of general-purpose hardware such as high volume servers. It transforms the network functions originally implemented on dedicated hardware, such as middleboxes or network appliances, to software running in the cloud environment or on general-purpose hardware [170].

A high-level overview of the NFV architecture is shown in Figure 18. In general, the NFV architecture is composed of three main components: NFV infrastructure (NFVI), virtualized network function (VNF), and NFV management and orchestration (NFV M&O). The NFVI provides the necessary virtual resources to support the execution of VNFs. The VNF is the network function implemented in software running over the NFVI. It is usually accompanied by the element management system (EMS). The NFV M&O covers the orchestration and lifecycle management of physical and software resources supporting the NFVI, and also the lifecycle management of VNFs. The NFV M&O also interacts with the operations support system (OSS) or business support system (BSS) landscape so that NFV can be integrated into an existing network management landscape [175, 176].

Although both SDN and NFV use network abstraction and virtualization techniques, they are quite different. SDN aims to separate network control functions from network forwarding functions, while NFV aims to abstract network forwarding and other networking functions from the hardware on which it runs. SDN abstracts physical networking resources, like switches, routers and so on, and moves decision making to a virtual network control plane which will decide the traffic forwarding. In this approach, the hardware continues to direct and handle the traffic. While NFV aims to virtualize all physical network resources beneath a hypervisor, it allows the network to grow without the addition of more devices. For example, when SDN executes on an NFV infrastructure, SDN forwards data packets from one network device to another. The SDN's networking control functions for routing, policy definition and applications run in a virtual machine defined by the NFV. Thus, NFV provides basic networking functions, while SDN controls and orchestrates them for specific uses [177]. In conclusion, NFV is closely related to SDN. They are highly complementary to each other, but not dependent on each other. They can be mutually beneficial and accrue greater value. For example, by adopting SDN techniques, NFV can enhance its performance [178].

NFV is a key enabler for 5G, which helps to virtualize the various appliances in the network. In 5G, NFV will enable virtualization of cloud RAN, which will help to deploy the networks and reduce the capital expenditure. Besides, NFV will provide infrastructure for network slicing, which permits a physical network to be separated into multiple virtual networks to support different RANs or various services. Future, NFV will enable the 5G networks to support serviced-based architecture by dynamically creating service chains. Therefore, NFV makes the 5G networks elastic and scalable, improves the agility and simplifies the management [179].

However, the M&O of SDN and NFV is still an open challenge in 5G and 6G, which faces the complexity, reliability, security, and multitenant issues. First, although the management responsibility for the various parts of the network and service provisioning is well defined in the SDN and NFV, the entire complexity of the M&O will increase significantly for the separation of control and management, the multivendor system, the improved functionalities, real-time resources allocation, dynamic network conditions, and network slicing. Second, owing to the increased complexity of the M&O, it is more likely for the network to have the unexpected and unforeseen "black swan" type of failures, which may be hard to deal with. Thus, the SDN and NFV face issues in terms of reliability and security. Third, the 5G and 6G network is expected to be multitenant, where network operators and service providers share the same physical infrastructure. Thus, the network M&O has to deal with the corresponding multitenant issues [180].

##### 3.2.2 *Network slicing and its improvement*

Network slicing is a network architecture that enables the multiplexing of virtualized and independent logical networks on the same physical network infrastructure [181]. A network slice is a logically isolated end-to-end network that is tailored for a particular service type with agreed upon service-level agreement (SLA).

The concept of network slicing can be traced back to the late 1980s when the concept of the slice was first introduced in the networking field. Next-generation mobile network (NGMN) then defined the network slicing in the context of 5G [182]. As depicted in Figure 19, the network slicing concept mainly consists of three layers, namely the service instance layer, the network slice instance layer, and the resource layer. Each service instance represents a service provided by the network operator or third party. The network slice instance provides the service instance with the required network characteristics, i.e., a service instance is realized by a network slice. A network slice instance may be fully or partly, logically and/or physically, isolated from another network slice instance. Each network slice may contain zero, one or more sub-network instances, which may be shared by two or more network slices. A sub-network

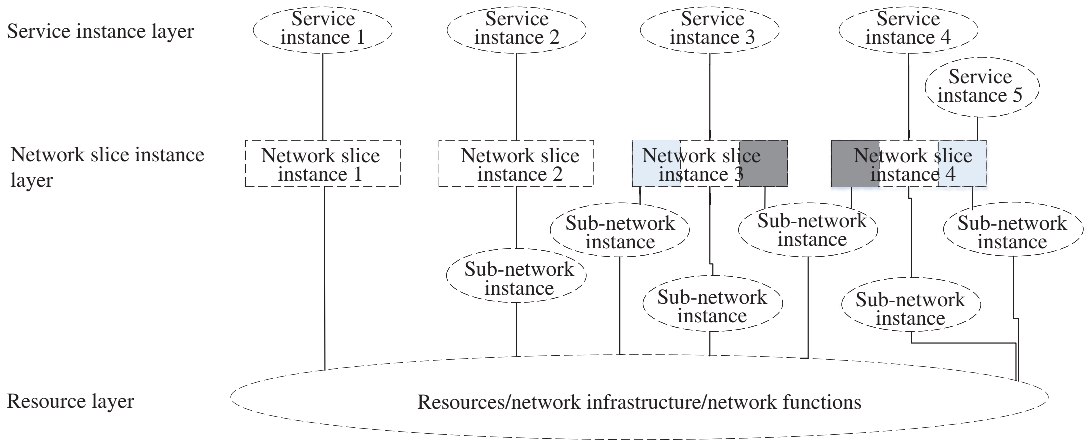

**Figure 19** (Color online) Network slicing conceptual outline [183].

instance can be a network function running on the physical/logical resources [183].

Network slicing builds a set of dedicated networks each customized for one type of services. Thus it can guarantee improved performance, better scalability, enhanced reliability and security, etc. [184]. Different slice types are defined in [185]: (i) eMBB; (ii) mMTC; and (iii) uRLLC. Network slicing is based on SDN and NFV and thus inherits most of the problems and challenges from them, i.e., the slice M&O in terms of the complexity, reliability, security, and multitenant issues.

In addition to the challenges inherited from SDN and NFV, network slicing has some additional considerations that must be addressed in the future 6G. (i) Slice isolation. Isolation is the most important property of network slicing, meanwhile, it is also the main challenge for its realization. To guarantee the service quality of each slice, different areas of isolation should be realized, including traffic, bandwidth, processing, and storage. In this case, the main challenge is the orchestration and control that need to be implemented to harmonize the different isolation techniques in different domains, while there is still not a holistic and final standardized network slice architecture for this. Besides, the isolation techniques significantly rely on the SDN and NFV technologies, which are not yet mature. (ii) Dynamic slice creation and management. To accommodate different services and meet diverse requirements, the efficient dynamic slice creation and deletion is necessary. However, it is challenging to create or delete slices because it has to guarantee that these operations have no effect on the currently running slices, which involves the isolation and security issues. Besides, the network slices should be able to scale dynamically with the varying load. Thus, efficient sharing is needed, which will also cause some issues like isolation and security. In this context, the lifecycle management of network slices is a critical problem to be solved [180, 184].

#### 3.2.3 *SBA and its evolution*

To meet the diverse requirements of services and introduce new services with as little impact as possible on other entities in the network, SBA has been introduced for 5G core network [186, 187]. Figure 20 depicts the reference SBA for 5G core network. The authentication server function (AUSF), as the name implies, is an authentication server. It holds the extensible authentication protocol (EAP) for authentication. The access and mobility management function (AMF) hosts all mobility management related functionality and terminates the non-access stratum (NAS) and N2 reference point messages. NAS messages are sent between the UE and the AMF for mobility management, and between the UE and session management function (SMF) for session management. The policy control function (PCF) provides a policy framework incorporating network slicing, roaming, mobility management, and the SMF controls the user plane function (UPF) for session management (e.g., session establishment, modify, and release). The unified data management (UDM) stores subscriber data and profiles, and the UPF is the user plane gateway serving the UE by connecting the RAN nodes to the data network (DN). The application function (AF) enables dynamic policy and charging control for applications. The UE is the mobile terminal, and the next-generation radio access network (NG-RAN) is the new air interface for 5G. The DN may be, for example, operator services, Internet access, or third-party services. There are also two new defined functionalities, network function repository function (NRF) and network exposure

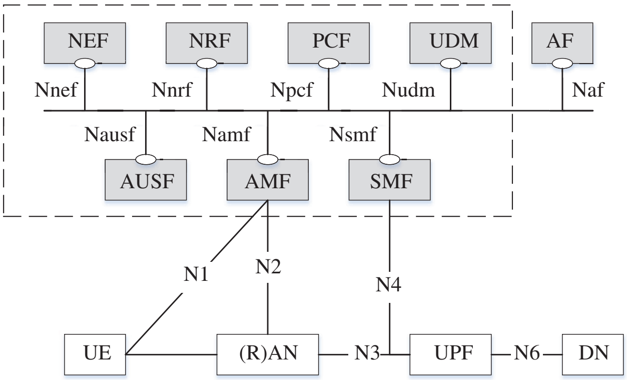

**Figure 20** SBA for 5G core network [188].

function (NEF). NRF can provide registration and discovery functionality to network functions, for them to discover and communicate with each other. NEF offers an interface gateway which allows external users, such as enterprises or partner operators, the ability to monitor, provision and enforce application policy such that users can be inside the operator network [188, 189].

The SBA is based on the related technologies across cloud computing, virtualization, microservice, stateless service, etc. Cloud computing provides on-demand computing to the SBA. Virtualization realizes more flexible and efficient resource management and usability. Microservice is an emerging and useful architectural design pattern, where the network is divided into small-granularity, highly cohesive, and loosely coupled services. Each service can realize a specific functionality. Thus, microservice can enable the SBA with flexibility, granularity, and independent scaling [189]. The case study and deployment considerations about the SBA in 5G can be found in [190]. However, the SBA also brings new security challenges to 5G and 6G. The SBA introduces the set of security features that enable network functions of the SBA to securely communicate within the serving network domain and with other network domains. These include network function registration, discovery and authorization security aspects, as well as the protection for service-based interfaces. SBA domain security is a new security feature in 5G and 6G. To ensure security between UEs in the SBA, security mechanisms such as transport layer security and open authorization are needed [191].

##### 3.2.4 *CSA*

Diversified objectives, changeable service scenarios and personalized user requirements not only require 6G network to have a large capacity, ultra-low delay, but also a remarkable degree of plasticity. In the face of the ever-changing business requirements in the distributed scenario, a 6G network service architecture should have enough flexibility and scalability and be able to make very fine-grained adjustments to the network in the control layer.

However, the SBA of 5G core network is based on coarse-grained configuration, which lacks real-time perception and dynamic adaptation to the change of service demand. Hence, the SBA of 6G core network should be significant cognitive function, i.e., CSA. The CSA has two features. First, it can accurately recognize target behaviors, scene semantics, and user characteristics. Second, it adjusts the network services adaptively and dynamically through the unified service description method. These features are supported by a cognitive interface, the lightweight learning agent, and the distributed service analyzing module. With the cognitive interface, the 6G core network has the ability of situation cognition and can perceive the change of service demand in a very fine-grained way. Then the lightweight learning agent makes the decision according to gained information by rule matching or approximate reasoning. At the same time, the distributed service analyzing module evaluates the service running state and provides a reference for the decision making of a lightweight learning agent. Thus, a complete cognitive closed loop of perception, decision-making, and evaluation is formed in the CSA.

Moreover, 6G core network functions will further sink to the edge of the network, i.e., edge core. With CSA and edge core, 6G core network will leverage edge computing to form a multi-center architecture to provide efficient, flexible, ultra-low delay, and ultra-large capacity network services. The original 5G core network running in the cloud will no longer directly participate in the control of the network. It just helps edge cores to communicate with each other. With core network sinking to the edge, the network response delay is reduced, and the flexibility of network management is improved. Besides, owing to the sinking deployment of the core network, the CSA will realize the whole network coverage from the core network to the UE. On this basis, the UE can adopt a variety of communication modes and can switch seamlessly when service scenario and requirement change. Edge core supports service adaptation, service migration, service collaboration, and service evolution through distributed service agents running in edge core. Taking service migration [192, 193] as an example, edge core discovers migration requirements and completes migration decisions through service cognition and invokes various modules through the north interface of the network layer to realize various processes such as state data transmission, cell handover, and user session switching. The whole process ensures low delay and transparency for users.

In this CSA enhanced by edge AI, 6G will further evolve from current "human-machine-thing" interactions to "human-machine-thing-genie" interactions [194]. These new ubiquitous, social, contextual, consciousness-based communication and control scenarios require intelligent service coordination of the real world and virtual world, as well as the efficient collaborative computing of various end devices and network nodes [195]. With the help of such intelligent and collaborative computing scheme, the 6G network can fully provide the ubiquitous immersive IoE services, including virtual scenarios and real scenarios by sensing various types of objective and subjective information.

However, current service capabilities of 5G network are no longer able to meet the requirements of future immersive IoE services. In the 6G era, end devices with various AI capabilities will seamlessly collaborate with a variety of edge and cloud resources. This "device + edge + cloud" distributed computing architecture can provide dynamic and extremely fine-grained service computing resources on-demand [196]. With the maturity of AI technology and the reduction of AI hardware costs, more and more smart end devices such as smart phones, AR/VR glasses, smart cameras, and smart TV, also including other IoT devices, will constantly increase in users' daily life [197]. The collaborative AI services among distributed end devices will become an important enabling technology for 6G. Therefore, how to integrate these scattered AI capabilities, and find the optimal combination of services to provide the best experiences for users based on different scenarios and user needs is a problem that manufacturers need to explore.

##### 3.2.5 *DEN$^2$*

As next-generation mobile communication networks target more on vertical industry scenarios, the communication technology innovations at local scope become more important. The overarching benefit of DEN$^2$ is to bring the communication services and intelligence towards the edge in order to realize the vision of pervasive intelligence. By doing so, it is not only possible to push the network performance to the upper limit, but also possible to explore the industry-grade isolation that is essential requirements to empower many industrial use cases in a cost-effective way.

However, a single deep edge node cannot leverage the power of large-scale system. A huge amount of relatively independent and isolated edge networks (e.g., in the order of millions) also cannot leverage the power of digital transformation platform at a larger scale, which may even become the hurdles of future industry innovation. Hence, the essential design of DEN$^2$ is to have large-scale networking based on cooperative and controllable deep edge node entities. Figure 21 illustrates the architecture of DEN$^2$. The goal of DEN$^2$ is not only about to provide the computational and intelligent capability to the edge, but also an architectural revolution that enables deep convergence of wireless communication and computing resources at the deep edge network with real-time adaptive collaboration, which is different from MEC or fog computing.

It has the vision to establish an ultra-high-performance platform which could provide unified access for all kinds of industry and non-industry terminals. On the other hand, DEN$^2$ is envisioned to become the key innovation platform for the future mobile communication system. In order to design DEN$^2$, there are a few enabling technologies to consider, e.g., industry terminal pass-through, the convergence of wireless and core networks, full autonomous self-driving network, new network protocols such as identification and address separation, perception, and user-centricity.

It also has the vision to enable highly distributed AI with moving the intelligence from the central cloud to the deep edge nodes, to allow reducing latency, costs, and security risks, thus making the associated business more efficient. The key capability of the DEN$^2$ from this perspective is native AI support, which covers data accessing, storage, processing, inferencing, knowledge distribution, etc. Hence, DEN$^2$ should consider how to handle data security and privacy as well as how to provide efficient synchronization on data and service by default, which requires a simplified but high-performance solution that could be done

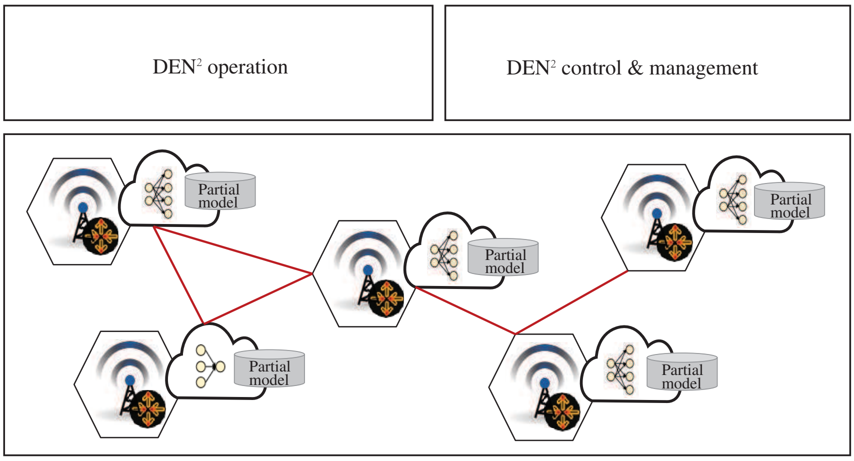

**Figure 21** (Color online) An illustration of DEN$^2$.

through the reconstruction of the protocol stack. DEN$^2$ also needs to consider how to support real-time collaboration among deep edge node entities.

#### 3.2.6 *CF architecture*

Since it was first introduced in 1970s [198, 199], cellular networks have become a core structure of mobile networks. Cellular networks offer many advantages in terms of practical implementations and the use of radio resources. However, the main inherent limitation of cellular networks is the boundary effect: the users at the cell boundary receive weak desired signal (due to path loss) and strong interference from other cells. As a result, these cell-edge users perform badly. The boundary effect is not a problem in the past and current mobile networks. This is the reason why the cellular-based topology is still the key option for mobile networks for many decades. However, in beyond 5G and future 6G communication systems, the high demand on data rate certainly will cause ultra-densified and heterogeneous BSs/APs deployment. While, with the density of BSs/APs, the cell coverage becomes smaller. The distance between BSs/APs has been reduced to tens of meters in 5G networks. Such densification of future wireless networks leads to more interference which makes the boundary effect a main bottleneck of cellular systems. From information theory, Refs. [116, 200] have shown that cellular networks are hitting the physical limit to the area spectral efficiency. Whatever the advanced technologies we use, we cannot overcome the boundary effect. Although there have been many techniques proposed in the last decades such as network MIMO, CoMP with joint transmission, and distributed antenna systems, their ability to overcome this bottleneck is limited. This is because these techniques are still based on cellular structures and/or require too complicated co-processing and high deployment costs. In addition to the issue of network convergence owing to the boundary effect, future wireless networks face many further issues, such as load balancing, handover overhead, interference management, to name a few. For example, heterogeneous communication networks need to interconnect with each other, and thus many issues in terms of routing, protocol, and management raise.

To solve the above problems, the CF (or cell-less) massive MIMO networks have been proposed in [117] as a practical and scalable version of network MIMO. Figure 22 shows a general system model of CF massive MIMO where many (thousands or more) APs jointly serve many user terminals in the same time-frequency resources. All APs are distributed in a large area (e.g., the whole city) and connected to one or several CPUs. A specific design of CF architecture using the SDN controllers for high-density smart city is illustrated in Figure 23 [201]. In this example, compared with the traditional cellular networks, where a (mobile) terminal needs to be associated with a BS/AP in advance, in this network, a terminal can decide to access several BSs/APs via different uplinks and downlinks depending on the wireless channel status and its demands. As a result, BSs/APs do not need to maintain a list of associated terminals. Instead, the associate control in the SDN controller will decide which BSs/APs the terminals should be associated via the control link. Moreover, the transmission control in the SDN controller will create dynamic uplinks/downlinks and backhaul links to support the joint transmission or reception between terminals and BSs/APs. That means the BSs/APs in the same group can cooperate with each other to

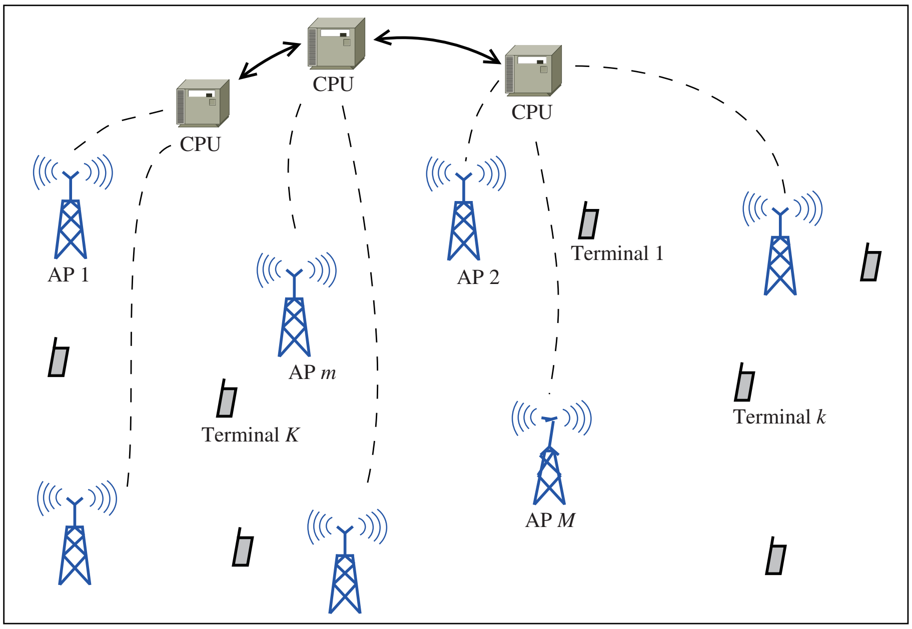

**Figure 22** (Color online) CF massive MIMO system model.

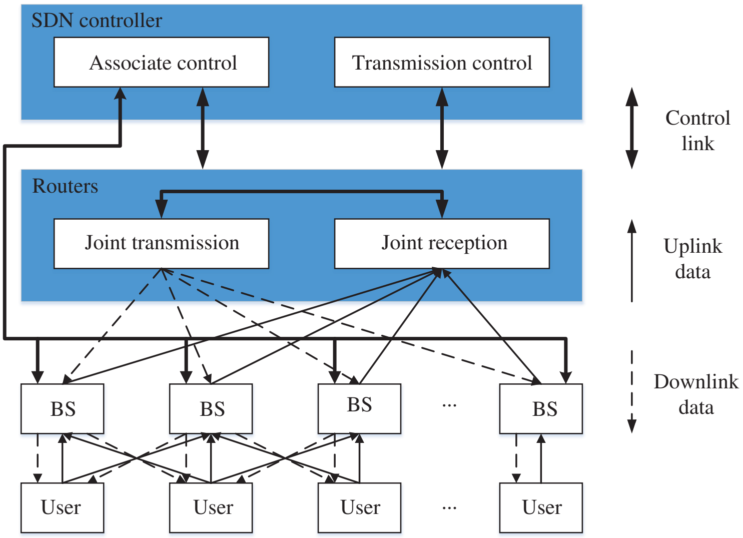

**Figure 23** (Color online) SDN-based CF architecture [201].

realize the joint transmission and reception for a specified terminal [201].

CF massive MIMO has been attracted a lot of research interest, and become one of the promising candidates for beyond 5G and towards 6G wireless communication systems [202–204]. The main benefits of CF massive MIMO are as follows.

• High network connectivity (coverage probability). In CF massive MIMO, there are no cells, and hence, no boundary effects. In addition, because the APs are distributed over the whole coverage area, the macro-diversity gain can be obtained. As a result, CF massive MIMO can provide very high network connectivity. All users in the networks can be offered very good services.

• Huge spectral and energy efficiency. Similar to colocated massive MIMO, CF massive MIMO offers many degrees of freedom, high array and multiplexing gains owing to the use of many service antennas (APs) to serve a much smaller number of user terminals. Therefore, CF massive MIMO can offer very high spectral efficiency and energy efficiency.

• Simple linear signal processing and low-cost devices. Because the number of APs is large, together with the law of large numbers, in many propagation environments, CF massive MIMO can have the favorable propagation and channel hardening properties. With favorable propagation, linear processing schemes, such as maximum ratio (MR), ZF, MMSE, can be used because they are nearly optimal. With channel hardening, the signal detections, power control, and signaling control can be done based only on the statistical channel knowledge (large-scale fading). This simplifies the system signal processing significantly. In addition, in CF massive MIMO, each AP has a few antennas. Therefore, CF massive MIMO is expected to be built by low-cost, low-power components and simple signal processing APs.

To achieve completely the above benefits of CF massive MIMO, many challenges need to be tackled. Some important future research directions on CF massive MIMO are listed below.

• Scalable signal processing. In [117], MR processing is proposed to be used at the APs. With MR processing, each AP just uses its local channel estimates to precode the symbols intended for all terminals. There is no sharing of instantaneous CSI among the APs. The communication between the CPUs and the APs is mainly limited to the payload data and power control coefficients. MR processing is simple and can be implemented in a distributed manner to offer good performance. Other linear processing schemes such as ZF and MMSE are much better than MR processing in terms of the system performance [205]. However, they cannot be directly implemented in a distributed manner. So the system is not scalable, i.e., it is not implementable when the number of APs/terminals grows large. Thus, designing a scalable signal processing that performs closely to the ZF and MMSE schemes is a challenging and important research direction.

• Scalable power control. In CF massive MIMO, because the APs and terminals are distributed in a very large area, the near-far effects and inter-user interference need to be controlled properly. Therefore, power allocation is very important. In [117, 206], the power controls are proposed to maximize the minimum rates and the energy efficiency of the system, respectively. These power controls are done at the CPU via solving a sequence of convex second order cone programs, under the assumption that the CPU has perfect knowledge of large-scale coefficients of all AP-terminal links. These schemes are not scalable with the network size (the number of APs/terminals). In [121], a distributed power control scheme is proposed where the power control is done at the APs with local knowledge channel conditions. This power control scheme works well, and most importantly, is scalable. However, compared to the centralized scheme in [117], the performance of this distributed power control is still far below. In addition, because this scheme is a heuristic power control, its performance depends a lot on the propagation environments. Scalable power control is still a very important open problem of CF massive MIMO.

##### 3.2.7 *Cloud/fog/edge computing*

**(1) The evolution of computing technologies.** Over the past decades, computing paradigms have evolved from distributed computing to centralized cloud computing. Now the pendulum swings back. Cloud computing is an architecture with centralized resources and management in the clouds, referring to data centers, backbone Internet protocol (IP) networks and cellular core networks [62]. Owing to centralized resources and management, it enables end devices and consumers with elastic on-demand resource allocation, reduced management efforts, flexible pricing model (pay-as-you-go), and easy applications and services provisioning [207]. However, this main advantage of cloud computing — consolidated resources and management — is also its main weakness. Centralized resources and management mean that functions and controls are located far from where tasks are generated. Owing to long physical distance, limited communication bandwidth, intermittent network connectivity, etc., cloud computing alone cannot meet the requirements of many delay-sensitive applications in 5G, such as automatic driving. Aiming at providing cloud computing capabilities at the edge of the network, in 2014, the industry specification group within European telecommunication standards institute (ETSI) proposed MEC. As defined by ETSI, MEC is a network architecture that provides IT and cloud-computing capabilities within the RAN in close proximity to mobile subscribers [208]. In March 2017, the ETSI expanded the scope of MEC and after that replaced the term "mobile" by "multi-access" [207]. The edges of non-mobile networks are also being considered in MAEC. Fog computing, first initiated by Cisco, is further promoted by OpenFog consortium to extend and generalize edge computing [209]. The OpenFog consortium defines fog computing as "a system-level horizontal architecture that distributes resources and services of computing, storage, control, and networking anywhere along the continuum from cloud to things" [210–212]. This thanks to the proliferation of smart devices, which can connect with each other and provide shared and flexible resources along the cloud-to-things continuum, driving the new service model call fog as a service technology [213]. A key difference between MEC and fog computing is that MEC functions only

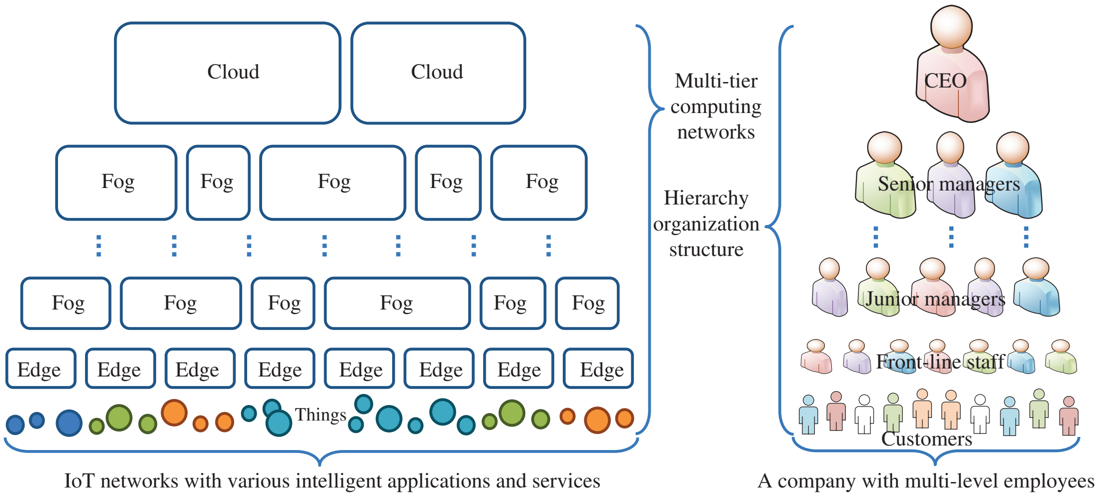

**Figure 24** (Color online) Multi-tier computing network architecture, which integrates cloud, edge, and fog computing technologies to enable intelligent services for anything at anytime and anywhere [216].

in stand-alone mode, while fog computing has multiple interconnected layers and could interact with the distant cloud and the network edge [207, 214]. In [215], the combination of AI and edge computing was introduced for dealing with several emerging future communication issues.

**(2) An integrated multi-tier computing network.** Cloud computing, edge computing, and fog computing are not competitive but interdependent. They complement each other to form a service continuum, and fog is the bridge that connects centralized clouds and distributed edges of the network [209]. For example, together with the edge, fog ensures timely data processing, situation analysis, and decision making at the locations close to where the data is generated and should be used. Together with the cloud, the fog supports more intelligent applications and sophisticated services in different industrial verticals and scenarios, such as cross-domain data analysis, pattern recognition, and behavior prediction. Thus, an integrated multi-tier computing paradigm involving collaborations between cloud computing, edge computing, and fog computing is inevitable in the future.

Multi-tier computing networks can be thought of as a large company with a top-down, multi-tier organization structure: managers and employees at different levels in the company have different resources, capabilities, and responsibilities in terms of data access and processing, task assignment, customer development, and decision making, as shown in Figure 24 [216]. Cloud computing is equivalent to the top hierarchical level in the company, possessing the most information sources, the strongest analytical intelligence, the maximum storage space, and the highest decision-making authority. As such, cloud computing is expected to handle challenging tasks at the global level, such as cross-domain data analysis and processing, abnormal behavior diagnosis and tracing, hidden problem prediction and searching, new knowledge discovery and creation, and long-term strategic planning and decisions. Edge computing, on the other hand, is equivalent to front-line staff, having the least resources and capabilities but being able to directly interact with customers in different application domains. Therefore, edge computing is good at handling delay-sensitive tasks at the local level, such as data collection, data compression, information extraction, and event monitoring. Between the cloud and the edge within the network, there is fog computing, which is equivalent to midlevel management in the company. Like an efficient management system with many levels of resources, duties, and responsibilities, fog computing is a hierarchy of shared computing, communication, and storage resources that can collaboratively handle complex and challenging tasks at the regional level, such as cross-domain data analysis, multi-source information processing and on-site decision making for large service coverage. Because user requirements are usually dynamic in terms of time and space, fog computing can provide a flexible approach to incorporate distributed resources at different geographical or logical locations in the network, thus offering timely and effective services to customers.

## 4 New paradigm shifts

To realize the performance metrics and application scenarios of 6G, the current 5G wireless communication networks are expected to have four new paradigm shifts, i.e., global coverage, all spectra, full applications, and endogenous network security. Global coverage utilizes satellite communication, UAV communication, terrestrial communication, and maritime communication to achieve a space-air-ground-sea integrated network. All frequency spectra will be fully explored, including sub-6 GHz, mmWave, THz, and optical frequency bands. Full applications will be combined with communications, computing, control/caching, and AI to enable higher intelligence. Endogenous network security will also be considered when developing 6G networks, both for physical layer and network layer.

### 4.1 Global coverage: space-air-ground-sea integrated networks

There have been some preliminary studies on the integration of different networks, including the integration of 5G and satellite network [217–220], 5G and UAV network [221–223], and space-air-ground integrated networks [224–227]. However, the comprehensive integration methodologies of space-air-ground-sea networks for global coverage have not been well studied. In this subsection, we first summarize the recent advances, technical challenges, and potential opportunities in satellite communication networks, UAV communication networks, and maritime communication networks, respectively. Then, we introduce a feasible integration approach of space-air-ground-sea networks and shed light on the future research directions toward the space-air-ground-sea integrated 6G system.

#### 4.1.1 *Satellite communication network*

Satellite communication networks can provide valuable benefits, e.g., seamless wireless coverage, for extending current terrestrial communication networks, and are deemed as promising solutions to be incorporated in future wireless network architectures [217–220,228,229], as shown in Figure 25. Multi-beam satellites have been widely adopted in satellite communication systems as they can provide increasing wireless data rates. Commonly for multi-beam satellite communication systems, a four-color frequency reuse scheme where adjacent beams are allocated with non-overlapping frequency spectrum (or different polarizations) is adopted to mitigate the co-channel inter-beam interference. Recently, a more aggressive full frequency reuse scheme has been advocated to enhance the spectral efficiency of satellite communications further. Nevertheless, the inter-beam interference owing to full frequency reuse should be carefully considered. Note that it is usually not straightforward to extend the interference mitigation approaches widely adopted in terrestrial communication networks to satellite communication networks owing to the significantly different channel propagation properties and the practical satellite payload limitations.

There exist plenty of technical challenges associated with future satellite communication networks.

(1) Accurate modeling of the satellite channels is of vital importance to the physical layer transmission design of satellite communication networks. Different from the terrestrial propagation environments, satellite wireless channels usually exhibit distinctive propagation properties, e.g., low rank properties and long propagation delay. Therefore, satellite channel properties need to be further investigated and characterized.

(2) With the capability of enhancing the spectral efficiency and energy efficiency, etc., massive MIMO has been widely recognized as a core ingredient of cellular wireless networks for 5G and beyond. The application of massive MIMO in satellite communication networks is rather challenging due to, e.g., size, weight, cost, power consumption, feeder link restrictions, etc. Deployment of massive MIMO for future satellite communication networks should be carefully designed with the potential practical concerns taken into account.

(3) Similar to the terrestrial cellular counterpart, satellite communication networks will involve multiple satellites, which cooperatively provide services to user terminals. Some typical multi-satellite network architectures include cooperative satellite transmission by sharing the same orbit window, different clusters, and multi-satellite relay transmission, etc. Resource allocation poses one key challenge in satellite communication networks.

(4) As a complementary solution for global seamless and ubiquitous coverage, satellite communications will play important roles in 5G and beyond. It is widely expected that satellite communication networks will be integrated into the 5G and beyond terrestrial communication networks. The major challenges

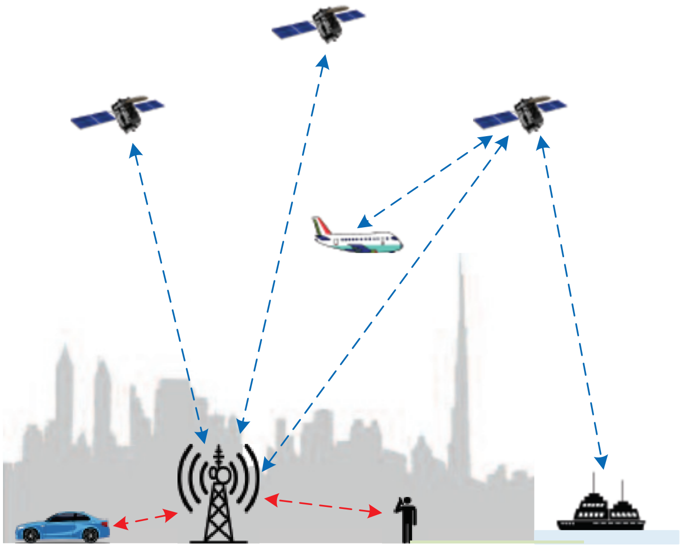

**Figure 25** (Color online) Integration of terrestrial and satellite communication networks.

in the integration of satellite and terrestrial communication networks are related to the physical layer transmission and media access control (MAC) protocols, e.g., random access, HARQ, etc.

##### 4.1.2 *UAV communication network*

With the inherent properties such as flexibility, mobility, and adaptive altitude, UAVs have been considered as an indispensable component and a promising technology in future wireless networks. UAVs can either be used as new aerial communication platforms [230,231], i.e., BSs, or be served as new aerial users in different scenarios [232]. We firstly classify UAVs according to different altitude platforms, so-called low altitude platform (LAP) and high altitude platform (HAP), respectively. When UAV is applied to the actual specific wireless network, in addition to the capability of the UAV itself, its environmental status, such as its own flight altitude and other factors must also be considered. According to the flying altitude of UAV, the corresponding communication network can be divided into two types: LAP-based communication networks and HAP-based communication networks.

LAP-based communication network has many unique characteristics, such as diverse and changeable topologies, switching constraints, and 3D transmission characteristics. With regard to topology structure, the meshed UAV structure has a good performance in terms of reliability and flexibility. In [233], the performance of mmWave UAV mesh network in beam management and network self-healing was studied. In addition to the above characteristics, there are three mechanisms playing important roles in LAP-based communication networks. Firstly, in order to complete military or civilian tasks, LAPs should be moved to the target regions by using a variety of effective movement control mechanisms, which include single-LAP movement control and multi-LAP cooperative mechanisms. The authors in [231] proposed a relatively simple mechanism to control all UAV nodes' desired trajectory, which minimized the total flight distance of all UAVs in motion. Secondly, after LAPs are moved into target regions, networking mechanisms take effect in LAP networks, which can improve QoS and network resource utilization efficiency. Besides, good network mechanisms can respond well to configuration changes in LAP networks. Thirdly, transmission mechanisms can help LAP-based communication networks provide reliable and high-speed data transmission. In [234], the authors proposed a broader neighbor discovery scheme in term of network recovery, which can improve the packet-delivery ratio and reduce the total network disconnection time by increasing the transmission power of 'Hello' messages. Furthermore, in the civil field, LAP networks also face various challenges related to network security [235].

HAP-based communication networks have witnessed an unprecedented improvement in providing mobile communications and broadband wireless access services. As a typical HAP-based communication network, the airborne network have several unique characteristics. First, most of these network nodes will have multiple links to achieve network reliability, high capacity, and low latency. Second, most of the nodes will be mobile so both the network links and the network topology will vary with time, some faster than others. Third, the distances between adjacent nodes will vary significantly, from hundreds of meters to tens of kilometres. Fourth, the power supply to the nodes will be limited, so the energy efficiency of each node impacts not only the operation cost, as in the case for terrestrial networks, but also commercial viability. Fifth, when applicable it would be highly desirable for antennas on the airborne platforms to be conformal in order to meet the aerodynamic requirements and maintain mechanical integrity.

Compared to conventional BSs or other aerial platforms, the advantages of HAP-based communication networks are their abilities of wide area coverage, favorable HAP-ground channel characteristics, rapid deployment, and quick response to temporal and spatial traffic demand [236]. Firstly, in consideration of the above advantages, HAP-based communication networks have attracted several prospective studies. The authors in [237] proposed single HAP and multiple HAP LTE cellular capacity analysis and integrated LTE technology into HAP-based communication networks. The study in [238] aimed at evaluating a pilot-based estimation technique for single-carrier frequency division multiple access (FDMA) LTE deployed via HAPs and analyzed the single-carrier FDMA performance on HAPs with pilot-based channel estimation. In [239], the authors proposed a novel framework for maximizing the sum rate of a relay-aided HAP drone wireless system when the CSI was not available, which can provide broadband wireless connectivity to ground users effectively. The authors in [240] investigated a vertical framework to backhaul/fronthaul small-cell base stations via networked flying platforms, which utilized HAPs and free space optics to transport traffic between the access and core networks. Secondly, owing to the special stratospheric transmission characteristic, the HAP channel model shows differences with other UAV channel models in the 3D transmission nature. The authors in [241] proposed a non-stationary 3D wideband geometry-based stochastic model (GBSM), which is more practical to characterize the HAP-MIMO channel. Thirdly, there are several key challenges for future HAP-based communication networks. These key challenges include safety and regulations, seamless integration with existing networks, optimized telecommunications payload, optimized signal processing and protocols, needs for high-speed backhaul/fronthaul links [236], as well as interference dynamics and limited energy [242].

Similar to satellite communications, UAV can be a promising solution for emergency wireless systems in cases of natural disasters or severe ground disruption [234, 235]. In comparison, UAV shows lower cost, lower latency, and better SNR than satellite communications. However, the high velocity of UAV leads to dynamic channel characteristics over time and space in air-to-ground and air-to-air communications. The non-stationary channel of UAV also brings some challenges to coverage and connectivity. In addition, airframe shadowing is also a challenge caused by the structural design and rotation of the UAV [233].

Some possible future research directions on UAV are listed below.

(1) When UAVs are used as flying BSs, the battery draining of UAVs could cause network nodes to fail. Therefore, the UAV charging or replacement scheme needs to be investigated to ensure the network coverage function normal.

(2) ML can be used to predict the users' mobility and their load distribution, which enables cache-enabled UAVs to dynamically optimize their deployment and trajectory. Efficient methods need to be developed to combine ML with cache-enabled UAVs.

(3) It requires a new multiple access technology to make use of UAVs and mmWave technologies in 5G communication systems. Besides, mmWave technologies need to be investigated during harsh weather conditions.

(4) In drone-aided networks, UAVs can provide a variety of services, such as caching, computing, network controlling, and drone cells. In such networks, UAV scheduling needs to be investigated to achieve high energy efficiency.

(5) UAV communications involve some security and privacy problems such as eavesdropping and jamming. These problems may pose threats to personal information, so it needs to be comprehensively investigated.

(6) High-speed mobility support and conflict avoidance are other challenges that need to be developed in the future.

(7) In order to make the UAV communications more efficient in practice, more practical channel models, including humidity, temperature, obstacles, suburban and urban environments, need to be investigated.

(8) Antenna aperture efficiency, scanning angular range and scanning speed are some of the issues to be addressed.

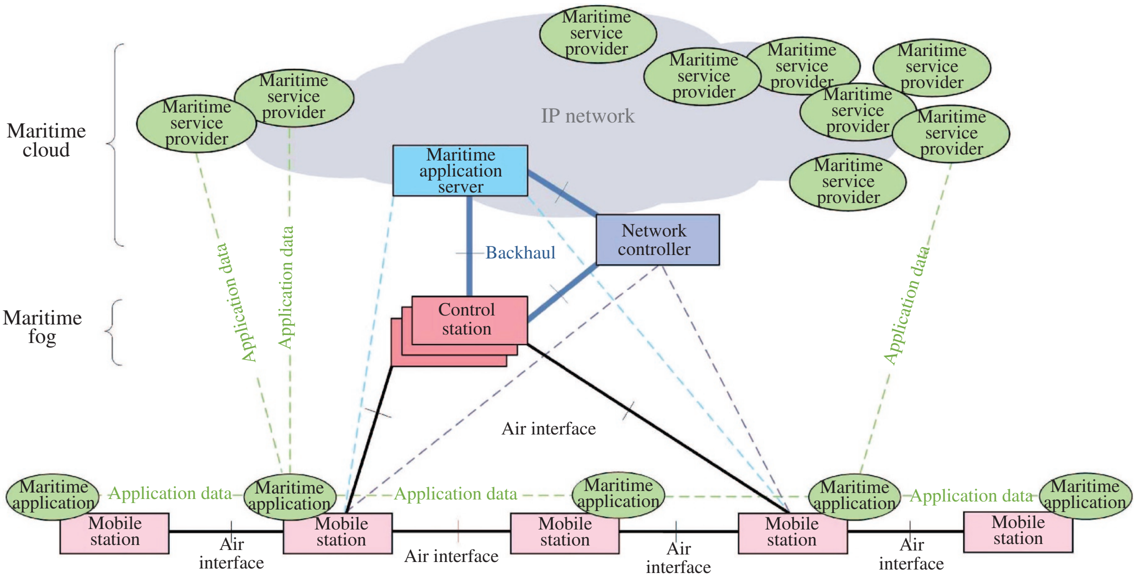

**Figure 26** (Color online) Maritime MTC network functional view and topology for service-centric and software-defined networking [244].

#### 4.1.3 *Maritime machine-type communication network*

The oceans cover more than 70% of the Earth's surface, and the international maritime shipping industry is responsible for the carriage of around 90% of world trade. Although 5G is all about enabling revolutionary use cases, maritime communication, particularly, maritime machine type communication (MTC) has not yet received enough attention in the 5G community. It can be foreseen that maritime MTC will become an imperative component in the evolving landscape of the communication system beyond 5G.

Progresses in IoT in the maritime domain for the modernization and mobilization of maritime related businesses as well as maritime safety and traffic management continue to challenge the traditional maritime communication systems, which have gradually shown their incompetence in meeting the ever-increasing demand and unique requirements from maritime IoT. These requirements and challenges include: (1) ubiquitous connectivity and service continuity, (2) traffic nonuniformity, (3) service-centricity, (4) device heterogeneity, (5) simplicity and reliability, (6) capacity and scalability, (7) inter-operationality, and (8) radio spectrum internationality [243].

The unique requirements of maritime IoT warrant not only a unique network and air interface design but radio spectrum allocation as well. To materialize this concept, we need the following.

• System architecture. The first and foremost requirement of maritime MTC is the provision of ubiquitous connectivity between vessels and shore over open oceans worldwide to ensure maritime service continuity. This poses a unique and serious challenge because unlike terrestrial cellular communication where wide area wireless coverage is provided via mass deployment of BSs, it is obviously unrealistic to cover the open oceans with such BSs. The solution is the deployment of a satellite MTC network to form a space-earth integrated maritime MTC system as graphically illustrated in Figure 26 [244]. Solid lines denote physical interfaces and dashed lines denote logical interfaces. The control station can be either the satellite space station or shore station. This space-earth integrated system provides seamless global maritime MTC to support various maritime IoT services, including navigation, observation and monitoring, and dynamic networking for emergencies, enabling global maritime information resources management, coordination, and optimization.

• Air interface. Communication at sea differs significantly from its land-based counterpart. The unique maritime MTC requirements impose great challenges to not only the maritime MTC network architecture but also the air interface [245]. At least three types of wireless communications can be identified for a full-fledged maritime MTC system: (1) satellite communication for offshore and worldwide coverage, (2) terrestrial communication for nearshore high-traffic area deployment where higher spectral efficiency/capacity and latency are critical, and (3) proximity communication for maritime IoT proximity services.

• Radio spectrum. International spectrum has been allocated for maritime MTC by ITU [246]. Specif-

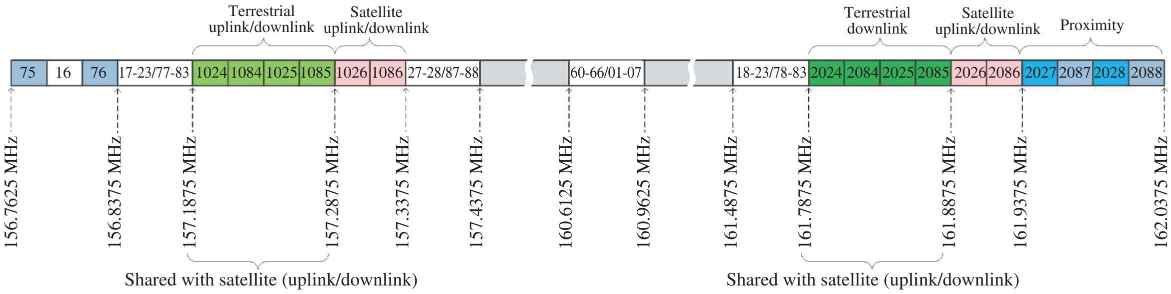

**Figure 27** (Color online) International frequency allocations for maritime MTC [243, 246].

ically, as shown in Figure 27, frequency channels 2087, 2088, 75, 76, as well as channels 2027 and 2028 in the very high frequency (VHF) maritime mobile communication band (156–174 MHz) are allocated for the maritime MTC proximity communications. Also, in the VHF maritime mobile communication band, paired channels 24, 84, 25, and 85 are allocated to the maritime MTC terrestrial communication, with the lower legs (i.e., channels 1024 and 1085) to uplink, and upper legs (i.e., channels 2024, and 2085) to downlink for frequency division duplex (FDD) operation. Paired channels 26 and 86 are dedicated to satellite with the lower leg channels 1026 and 1086 to uplink, and upper leg channels 2026 and 2086 to downlink. These channels can also be used as duplex channels (both uplink and downlink) for optional TDD operations.

While still in its early stage of development, the maritime MTC technology, backed up by the internationally allocated VHF spectrum, will continue to evolve beyond 5G with the culmination and synergy of various technological efforts to bring the maritime IoT concept one step closer to reality.

##### 4.1.4 *Space-air-ground-sea integrated networks*

It is generally recognized that, owing to their limitations of radio spectrum, service geographical area coverage, and operation cost, 5G terrestrial communication networks alone cannot achieve ubiquitous, high-quality, and high-reliability services at anytime and anywhere, especially in coping with the upcoming trillion-level connections of IoE devices in remote areas. In order to provide truly ubiquitous wireless communication services globally, it is imperative to develop a space-air-ground-sea integrated network to achieve worldwide connectivity and make various applications accessible especially in remote areas [226, 247, 248]. Different from the traditional terrestrial coverage, the 6G space-air-ground-sea network will integrate underwater communication networks to support broad-sea and deep-sea activities. However, the underwater communication exhibits different propagation characteristics from terrestrial networks, and the complex and unpredictable underwater environments can cause plenty of issues to be resolved, such as intricate network deployment, severe signal attenuation, and physical damage to equipment [249].

In the space-air-ground-sea integrated network, various networking technologies have their pros and cons in providing services, in terms of coverage, transmission delay, throughput, reliability, etc. Via effective inter-networking, different network segments can cooperate to support seamless service access and enhance service provision in an efficient and cost-effective manner. Specially, satellite communications can complement terrestrial networks for service access in areas with limited or no terrestrial network coverage (e.g., remote areas, disaster scenarios, and open seas). Meanwhile, the complementary properties of satellite link (wide coverage) and fiber-optical backbone (high data rate) can be considered as alternative backbone technologies to wireless backhaul. UAV communications can help to alleviate the terrestrial network burden and to enhance service capacity in congested locations with highly dynamic data traffic load. In addition, the satellites/UAVs with remote sensing technologies can support the reliable acquisition of monitoring data and assist terrestrial networks for efficient resource management and planning decisions [250].

The space-air-ground-sea integrated network can have a layered heterogeneous architecture in essence. It is vital to consider the efficiency of the dynamic collaboration of multi-dimensional heterogeneous resources in the integrated system, for data transmission, processing, sensing, and caching [251]. To facilitate efficient operation and management in the integrated network with multi-dimensional resources and highly dynamic networking environment, an SDN-based hybrid and hierarchical control architecture should be in place, as shown in Figure 28. Specifically, massive network information is required at the SDN controllers to effectively make centralized decisions on network operation and service provisioning.

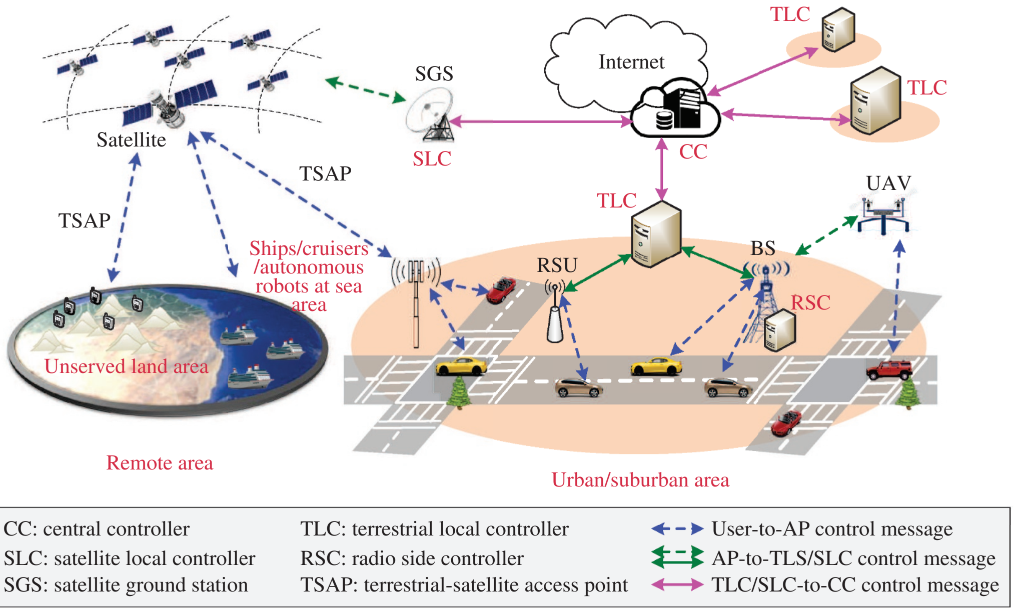

**Figure 28** (Color online) Space-air-ground-sea integrated network control architecture.

Such information includes statistical data and periodic updates on user mobility, temporal and spatial distribution of service demands, QoS requirements, and network resource availability. The network control architecture should be versatile and flexible.

- Hybrid control architectures for different scenarios. The SDN controllers incur high signaling overhead for network management owing to the collection of massive network information [225]. To avoid the unnecessary signaling overhead for efficient resource utilization, distributed control architectures are suitable for services in remote areas. Specifically, owing to low user density, resource management in such scenarios is relatively straightforward, and distributed algorithms can generally achieve satisfactory performance in local operation without involving SDN controllers. In urban/suburban scenarios, on the other hand, the network management is complex, resulting from high service demands, multi-dimensional resources, and complicated communication environments. Hence, SDN controllers are beneficial in these scenarios to maximize service quality and network performance.
- Hierarchical control architectures for urban/suburban scenarios. For scalability, SDN controllers in urban/suburban scenarios should be organized in a hierarchical manner, where SDN controllers at different levels target at network operation in different scales, i.e., for local, regional, national, and global domains. For instance, each RSC schedules transmission and processing within its coverage area with fast response, while the local controllers coordinate the RSCs to allocate resources in the neighborhood of multiple RSCs. Further, the central controllers act as coordinators for the local controllers to perform network-wide resource management for end-to-end service delivery and seamless coverage even in high mobility situations.

To effectively integrate various network segments with different scales and diverse radio access technologies in the space-air-ground-sea network, there exist many challenges and opportunities that require further studies, including the following.

- Mobility management. In the integrated network, there are multiple types of mobility introduced by satellites (especially low Earth orbit (LEO) satellites), UAVs, ships, terrestrial users (especially vehicular users), and other maritime devices. All the diverse mobility patterns should be properly accounted for the network operation and service provisioning. For seamless mobility management, it requires innovative engineering solutions, especially for vertical handoff between heterogeneous network segments, in addition to horizontal handoff within the homogeneous segment. When considering an LEO satellite network as the backhaul, its capacity is constrained by the highly dynamic satellite constellation topology and the limited contact duration among LEO satellites, which further complicates the decision process in selecting satellite or terrestrial backhaul.
- Transport networking protocols. The transmission control protocol (TCP)/IP protocol suite has been widely used in both terrestrial and satellite networks. However, as the protocol suite was originally designed to support best-effort services in the wireline Internet with arbitrary network topologies, it has some limitations when applying to the future integrated network. First, satellite communication networks (e.g., Starlink) have dynamic but deterministic topologies, which will deviate even more from random topologies in the Internet in coming years with densely deployed LEO satellites. Second, LEO satellites have high mobility and the communication delay varies significantly. The large variations of round trip time (RTT) can affect the transmission rate and degrade the TCP performance. Third, the transmission rate asymmetry in satellite uplink and downlink channels also affects the TCP performance. A low-rate uplink reduces the arrival rate of acknowledgments (ACKs) and the increase of the TCP sending rate, leading to poor channel utilization in the downlink. Last but not least, with emerging network virtualization technology such as in 5G cellular systems, the future integrated network will be logically circuit-switching under the network slicing framework. As a result, in-path network intelligence can be achieved for effective and efficient transport protocols, different from packet switching in the Internet that relies only on end nodes in transmission control [252]. Further research is necessary to exploit various characteristics of the integrated network in networking protocols for maximal performance.

• Routing strategies. The routing strategy in LEO satellite networks and UAV group networks is a major issue. When utilizing the LEO satellite networks to support long-distance communications, inter-satellite routing is indispensable to provide a reliable data transmission path for satellite users. Basically, LEO satellite networks are characterized by limited on-board processing and storage capabilities, dynamic network topology, uneven data flow, and high bit error rate. A LEO satellite network uses a constellation of several similar satellites to provide wireless networking services to the Earth [253]. The LEO satellite network topology in the whole inter-satellite communication process is time-varying and redundantly connected multipath may exist, which causes fast retransmit and recovery and delayed acknowledgments. Several cooperative routing algorithms for satellite networks have been proposed to improve the network throughout and end-to-end delay performance [254–259]. In UAV group networks [260, 261], the mobility and altitude of UAVs, transmitted power, inter-UAV distances, extraneous noise, uneven UAV access users' traffic distribution in both spatial and temporal domains, will largely affect the routing protocol [262] and conventional routing protocols fail to work. Several novel routing algorithms have been proposed recently for UAV group networks to achieve better performance [263–268]. Nevertheless, routing strategies which can adapt to high mobility, dynamic topology, and different routing capabilities in LEO satellite networks and UAV group networks are still in their early stage and need further studies.

• Energy efficiency. Different from terrestrial BSs which are mostly connected to the power grid at all time, the UAVs and satellites are powered by a battery and/or solar energy. The propulsion energy required to maintain the aloft and mobility of UAVs dominates their energy consumption, while the intense radiation and space-variant temperature affect the satellite energy consumption. With limited battery and solar energy capacity, the flight duration of UAVs is short and satellite functionalities for transmission, processing, and sensing can be severely constrained. Therefore, improving energy efficiency to increase the endurance of UAVs/satellites in the integrated networks remains a critical but challenging research topic.

The 6G wireless networks are envisioned to provide global coverage, support disparate radio access technologies, make use of all available radio spectra, and achieve strong network security. Different from the traditionally evolved wireless communication networks, the layered heterogeneous space-air-ground-sea integrated system will embrace high dynamics of the future 6G networks in provisioning diverse services with multi-dimensional resources. It is essential to leverage groundbreaking and cutting-edge techniques for system management and service optimization. Recent advances in SDN, AI, and network slicing technologies provide enabling tools for seamless integration of the heterogeneous network segments to maximize the benefits from their complimentary characteristics to support various new use cases and applications.

The SDN-based hybrid and hierarchical network control architecture will play an important role in efficient network control and inter-segment operation, and dynamic networking. Further, network slicing based resource management potentially can enhance customized service provisioning to different users with different granularities in various coexisting applications. Considering the massive and complex network information in the large spatial-temporal domains within the space-air-ground-sea integrated system, innovative AI-assisted engineering solutions are required for automation in system operation and control, to achieve flexibility and scalability in system management, to make proactive and real-time decisions, and to maximize the overall performance of the integrated system. Particularly, AI is a promising tool to automate and optimize network slicing, including determining the optimal slicing granularity, monitoring service level agreement for each service type, and the slice adaption in a dynamic network environment with uncertainties. In user mobility management, AI-based techniques should be developed to facilitate analysis and prediction of user mobility patterns for location-dependent big data informatics services such as in smart transportation. In addition, further research is required to investigate AI-based joint resource management for communication, computing, sensing, and caching in the different network segments. There exist various challenges and opportunities in exploiting contemporary AI techniques for the space-air-ground-sea integrated system. Various model-based optimization solutions developed in the past few decades for communication networks provide useful insight and pave a way for developing new AI-based solutions that are optimized for and pertain to space-air-ground-sea integrated networking. Painstaking efforts from both academia and industry are required over the next ten years to meet technical challenges towards developing the space-air-ground-sea integrated system.

### 4.2 All spectra: sub-6 GHz, mmWave, THz, and optical frequency bands

While sub-6 GHz and mmWave bands have been widely investigated, THz and optical frequency bands need further investigation. Owing to their varying wavelength and bandwidth, their channel propagation characteristics have large differences and should be carefully studied. Here we summarize works on channel measurements, propagation characterization, and channel models for different frequency bands. Then, we try to propose a general channel model framework for all frequency bands.

#### 4.2.1 *Sub-6 GHz bands*

Sub-6 GHz bands have been the primary working frequency in the third generation, 4G, and 5G owing to wide coverage capabilities and low cost [269], which are also indispensable in 6G. The propagation characterization and channel models in sub-6 GHz bands have been extensively investigated in 5G. The signal fading in propagation mainly originates from two aspects: large-scale fading depending on path-loss and shadowing, as well as small-scale fading coming from multipath propagation. Generally, the models of small-scale fading can be divided into two categories, deterministic and stochastic. Deterministic channel models aim to provide a realistic and site-specific description of the channel between Tx and Rx. Stochastic channel models condense the channel characteristics to a statistical description, e.g., the power delay profile (PDP). To close the gap between the above two approaches, a spatial channel model (SCM) has been developed to incorporate not only a random PDP but also a random angular profile by 3GPP [270]. Furthermore, as the standardized 3GPP 3D SCM model has been proposed for LTE-Advanced (LTE-A) to provide additional flexibility for the elevation dimension, thereby allowing modeling for 2D antenna systems [271]. To lay a solid foundation for 5G, many general 5G channel models that can support various 5G communication technologies and adapt to different scenarios have been proposed, such as COST 2100 channel model [272], 5GCMSIG channel model [273], and 3GPP TR 38.901 channel model [274]. However, in 6G, the higher performance requirements pose a new challenge for channel modeling to support more technologies and scenarios, including massive MIMO, direct V2V, and HST scenarios.

#### 4.2.2 *mmWave bands*

Spectrum has become scarce at traditional microwave bands, and thus mmWave communication operating between 30 GHz and 300 GHz is considered as a promising technology to provide ultra-high capacity in the future-generation systems [275]. mmWave wireless communication systems significantly differ from the conventional mobile communication systems using sub-6 GHz bands, which should consider the propagation characteristics and hardware limitations.

It is known that the target data rate of sub-6 GHz 5G mobile communications is Gbps level, and the target data rate of 5G mmWave is about 10 Gbps. There are two key ways to increase the wireless transmission data rate: one is by improving the spectral efficiency and the other is by using large frequency bandwidth or spectrum resource. The spectrum resource in the microwave frequency band has been applied to various wireless systems and is nearly exhausted. Therefore, in order to achieve the goal of about 10 Gbps data rate, people explored the mmWave spectrum resource in 5G. Although we can find much more spectrum resources in the mmWave band than in the microwave band, improving the spectral efficiency is still essential to achieve high data rates. In this context, the massive MIMO technology was proposed for 5G mmWave to improve the spectral efficiency, but at the expense of using tens or even hundreds of RF chains and antennas, which means high fabrication cost, power consumption, and volume. Furthermore, additional challenges are the A/D and digital-to-analog (D/A) converters with high sampling rates for wide frequency bandwidth and the real time processing of massive data.

The key observation from field channel measurement results is that mmWave propagation introduces severe path loss which increases with carrier frequencies rapidly. Therefore, directional transmission using large antenna arrays at both BSs and UEs is imperative to compensate the distance-dependent path loss with high antenna gain and reduces the multi-user interference, corresponding to the increase of signal-to-interference-plus-noise ratio (SINR). Thanks to the shorter wavelength at mmWave bands, large antenna arrays can be emitted in a certain physical size whereas the aperture of the array with the same number of elements will be remarkably enlarged below 6 GHz. Combining with full-dimension or 3D beamforming technology, mmWave communications can provide excess DOF in beam sweeping and not dramatically worse coverage range. Moreover, although the potential of mmWave directional transmission is exciting, ultra-dense small cell networks is necessary to reduce the average propagation distance and ensure the sum capacity of mmWave networks. The other distinguishing feature of mmWave systems is their vulnerability to blockage. Penetration losses at mmWave bands are much larger than those at lower frequency bands and seem to be site-related (e.g., building structures, materials, and incident directions). On the other hand, diffraction is extremely weak owing to the smaller wavelengths of mmWave signals in comparison with the dimensions of surrounding objects, while reflection becomes rich which will enhance the coverage in actual mmWave networks.

To progress the deployment of mmWave networks in real environments, there are some new considerations for the optimum network design and the coverage enhancement from channel measurement and modeling perspectives.

- High-efficient and flexible channel sounder design. It is essential to fully understand the channel characteristics based on channel sounding data. However, despite extensive channel measurement campaigns have been conducted in multiple indoor and outdoor environments at several mmWave candidate bands, there is an unfair comparison of mmWave channel characteristics across different frequency bands and scenarios employing different channel sounders. Therefore, flexible channel sounders are needed to support sufficiently high measurement speed, frequency bands flexibility, and multiple measurement scenarios.
- Full-dimension channel characterization. With the increase of the total number of antenna elements and carrier frequency, far-field distance of large antenna array will significantly increase, leading to spherical wavefront property within the coverage radii of mmWave small cells. On the other hand, to leverage spatial diversity or 3D beamforming technology, it is crucial to record spatial propagation characteristics (especially in elevation plane) where several sounding methods with respect to different antenna configurations are promoted, including: (1) rotated directional antenna, (2) uniform virtual array, (3) RF switch based antenna array, and (4) beamforming based phased array. Combining with high-resolution parameter estimation algorithms, more effective multipath components (MPCs) characterized by power, delay, and angles can be estimated based on original data.
- Multiple transmission schemes and applications. Existing mmWave channel models mainly concentrate on feature extraction from field channel measurement data or simulation data to describe frequency-dependent and site-specific channel characteristics in a more general manner. However, mmWave propagation characteristics are sensitive to the deployment environments and transceiver configurations, corresponding to different transmission schemes and application. Moreover, it is crucial to take account of the potential mmWave network architectures during measurement planning which may benefit by more precise system configurations. For example, cell radii will increase with the increase of BS antenna height because more diffraction and reflection signals over surrounding buildings can be received in dense urban environments. Conversely, medium range BSs placed at 2–3 m are characterized by requirements derived from microcell scenarios (e.g., street canyon), roadside scenarios, and relay schemes.

##### 4.2.3 *THz bands*

Traditionally, the 26.5–300 GHz frequency band is defined as the mmWave band and 300–10000 GHz as the THz band. In recent years, however, the definition of 100–10000 GHz (or 0.1–10 THz) as the THz band has been commonly accepted.

With the explosive growth of data traffic in wireless communication systems, THz frequency bands have been envisioned as a promising candidate to provide large bandwidth and supply sufficient spectrum resources for near future 6G [276–278]. Specifically, THz communication is able to realize high transmission rates from hundreds of Gbps to several Tbps. In addition, THz band commendably bridges the gap between mmWave and optical frequency bands [279]. Compared with mmWave frequency bands, THz communication possesses large transmission bandwidth and better security performance.

In contrast with optical frequency band, THz communication is much easier to tracking and alignment of beam, and adapts inconvenient climate conditions including fog, dust and so on. Enabled by these obvious advantages, extensive researches of THz communication have been developed worldwide. The United States, Japan, and the European Union have officially deployed 6G technology, achieve the enhance mobile broadband with a peak data rate greater than 100 Gbps, and plan to adopt THz band above 0.275 THz. In November 2019, 6G technology research and development working group and overall expert group were established, indicating the official start of 6G research in China. Furthermore, the world radiocommunication conference 2019 (WRC-19) has allocated the THz frequency band from 0.275 THz to 0.475 THz for mobile and fixed communication services [280].

In the forthcoming 6G wireless networks, THz communications are expected to support a wide variety of application prospects. Owing to the strong directionality and high path attenuation of THz waves, THz communication is applicable for indoor communication scenarios [281]. Based on the features of high data rates and low latency, it is available for THz communication to bring the better services to human life and facilitates the high-quality applications for different indoor scenarios. Notably, one of the most promising indoor scenarios is the wireless data center [282]. For the moment, the architecture of the traditional data centers is based on wired connections, and the wiring complexity and maintenance cost of the wired data centers is extremely high. To this end, the concept of wireless data centers has been proposed to solve the problem existing in the traditional case. Besides the potential indoor THz application scenarios mentioned above, THz communication will also be used for the secure communication scenarios [283]. When the UAVs accomplish the reconnaissance mission, the undistorted high definition (HD) video information can be transmitted to other combat units, e.g., UAVs, manned aircrafts, and tanks. In terms of the integrated space information networks, there are some typical THz communication applications, such as the satellite cluster networks, inter-satellite backbone networks, and satellite-to-ground networks. In contrary to the ground THz communications, the space applications in the atmosphere-free environment do not suffer from the atmosphere attenuation, which is vital for the long-distance space communications at THz band. Moreover, micro and nano scale communication scenarios are some potential applications in near future 6G, such as on-chip THz communication, nanoscale IoT, nanoscale sensing networks.

In general, there are three kinds of THz communication systems that are widely developed, including solid-state based THz systems, direct modulation based THz systems, and photoelectric combination based THz systems. Firstly, as for the solid-state based THz communication system [279], the sub-harmonic mixer is used as modulator and demodulator at the Tx side and Rx side, respectively. Noticeably, the super-heterodyne transceiver scheme is also adopted in the 220 GHz solid-state communication system where the 220 GHz sub-harmonic mixer performs the function of spectrum conversion. However, the solid-state THz system endures the phase noise deterioration, conversion loss, and it needs high gain power amplifiers. This solid-state THz system is easily integrated and saves the power energy. Secondly, with regard to the direct modulation based THz system, a continuous THz carrier wave is generated by the THz source, and the amplitude shift keying (ASK) modulation signal from the BB is loaded on the modulator to realize the modulation of the THz carrier wave directly. One may note that the direct modulation based THz system possesses high flexibility, achieves higher output power and is easily integrated with small size. However, this modulation mode is very difficult and endures relatively low spectrum utilization. Then, to the best of our knowledge, the photoelectric combination based THz system is able to achieve extremely high data rates and high bandwidth utilization. Accordingly, this photoelectric combination based THz system suffers from low transmitting power, short communication distance and high power consumption.

There are different ways to generate and detect THz signals, but for wireless communication applications, THz integrated circuits (ICs) are the most important. Up to now, lots of ICs at the 0.1–0.8 THz band based on SiGe, CMOS, GaAs, InP processes etc., have been reported. For low-cost THz signal generation based on the semiconductor process, the most substantial problem lies in the low output power that is usually bellow 0 dBm. For instance, a 270–290 GHz multiplier chain (×9) with an on-chip dielectric-resonator antenna (DRA) was designed in a commercial 0.1-μm GaAs pseudomorphic high electron-mobility transistor technology with cutoff frequencies $f_{\mathrm{T}}/f_{\mathrm{MAX}}$ of 130/180 GHz [276], where the

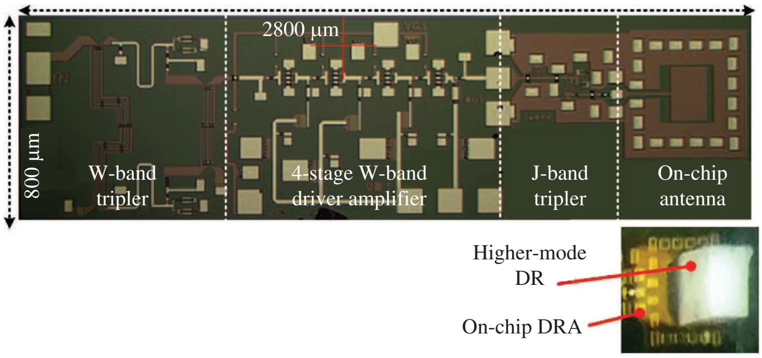

**Figure 29** (Color online) Microphotograph of the multiplier with on-chip antenna.

effective isotropic radiated power (EIRP) is around 2 dBm. The microphotograph of the chip and the on-chip antenna is shown in Figure 29.

There are a number of challenges for THz technologies to be employed in 6G. First, the propagation of electromagnetic waves at THz frequencies suffers from severe atmospheric losses, and it does not have strong penetration and diffraction abilities compared with microwave or mmWave. Second, with semiconductor technologies, it is currently very hard to produce high power THz Txs. Third, current THz Rxs tend to have much higher noise figures. The first challenge dictates that THz communications are more suited for short distance line of sight (LOS) applications on the ground, or long-distance links between satellites. The second challenge demands for research on innovative power amplifier designs. One promising solution is power combining using antenna arrays. The third challenge requires research on sensitive THz Rxs. For communications between satellites for 6G, high power THz Txs, highly sensitive Rxs, and adaptive antenna arrays at both the Tx and the Rx are three areas of significant importance.

THz systems for imaging have been around for over two decades. In contrast, research on THz communication systems is still in its infancy. Significant research effort is needed to develop high performance and low-cost device and packaging technologies to make them commercially viable for 6G.

##### 4.2.4 *Optical frequency bands*

The optical spectrum is already a key enabler for the global Internet. Optical fiber communication networks not only connect all continents, they but also form the backbone of modern communication networks that provide high speed data access to metropolis, cities, towns, and increasingly also to homes. Extending the optical fiber medium to include the free-space medium for last mile connectivity and for mobile access seems a natural step.

Therefore, in recent years, there has been a significantly increasing interest from academia and industry in optical wireless communication (OWC) technologies. Now, there is also a wide range of OWC technologies, as a result of the many use cases they can support. The main OWC technologies are (i) free space optical (FSO) communications, (ii) VLC, (iii) optical camera communications, and (iv) wireless networking with light, which is also referred to as light fidelity (LiFi).

The optical spectrum can be used to provide fast, safe, robust, and efficient next-generation communication systems. In terms of the optical bandwidth, it is three orders of magnitude larger than the spectrum resources available in the RF bands [284]. However, current OWC technologies require electrical-to-optical (EO) conversion and optical-to-electrical (OE) conversion, which both are accomplished by optical devices such as light emitting diodes (LEDs) and lasers at the Tx and photo-detectors (PD) or solar cells at the Rx [285]. The electrical bandwidth of these devices is limited, which limits the full exploitation of the optical spectrum resources. However, clearly research efforts have increased in this area within the last 15 years having led to step-change advancements. For instance, recent results show significant improvements of the bandwidth of organic light emitting diodes (OLED) leading to 20 times higher data rates compared to state-of-the-art data rates achievable with OLEDs and this is now reported in *Nature Communications* [286]. Moreover, it has been shown that it is possible to achieve link data rates of 15.7 Gbps with COTS LEDs that cost less than \$0.5 [287], as shown in Figure 30.

Generally, the detectors are square-law detectors, which leads to low Rx sensitivity. It is, therefore, important to continue with the development of new OE/EO conversion devices, which have high bandwidth in the order of the hundreds of gigahertz, and detector devices, which can operate close to the

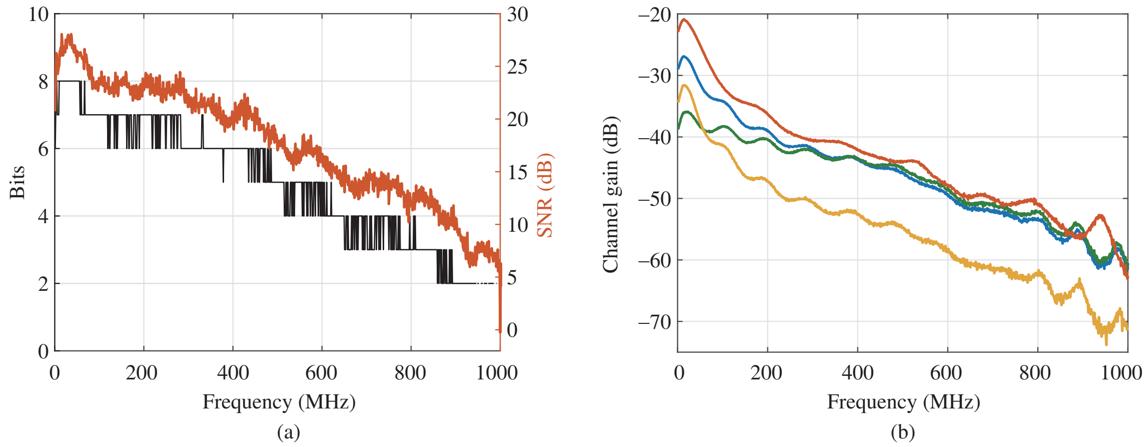

**Figure 30** (Color online) (a) SNR of a COTS red LED at a distance of 1.6 m and bit loading pattern. Binary phase shift keying (BPSK) is still achieved between 900 MHz–1 GHz which is well above the 3-dB bandwidth of the device. (b) Channel gains of the different colors (red, green, blue, and yellow) of the wavelength division multiplexing (WDM) system which achieved 15.7 Gbps with COTS LEDs [287].

quantum limit such as single photon avalanche diodes (SPADs) [288]. Moreover, it is important to consider coherent transmission systems for VLC and LiFi. Initial results have shown that coherent reception is feasible and achieves higher Rx sensitivity [289].

Besides, light beams can achieve near perfect energy concentration, making them a promising solution to achieve highest multiplexing gains while enabling very high energy efficiency [290]. The super high directional gain also makes the information transmission unlikely to be eavesdropped on, making the links safer than their RF counterparts [291]. Furthermore, as the optical signals are not subject to fading, because the size of the detector is significantly larger than the wavelength, OWC systems also have the potential to provide precise and reliable positioning services with centimeter-level accuracy [292–296]. Unlike global navigation satellite systems or other RF positioning systems, most optical positioning systems do not rely on fast pseudo-noise codes, thus they can get rid of precise synchronization units and expensive RF chains. Prior knowledge of user locations can be used to significantly improve channel estimation and beamforming in both RF and optical wireless BSs as well as enhance AI for resource allocation and mobility management [297].

There are commercially available products for indoor wireless access using light and many real-world pilots have been deployed [284]. An expansion of these networks to outdoor optical wireless networking will be the next stage of the development [298]. Because of advanced digital modulation techniques based on OFDM, the impact of ambient light and sunlight is almost negligible [299]. Moreover, ordinary solar panels can turn sunlight into energy and at the same time act as data detectors for high-speed optical wireless signals [285]. A last-mile proof-of-demonstrator of an ordinary solar panel acting as an optical wireless data receiver and energy harvesting device has been deployed at the Orkney island in Scotland/UK [300].

One of the major advantages of the optical spectrum for wireless communications is the containment and control of the radiated signals by means of simple optical subsystems. This means that it is possible to cover a few square meters down to a very small area of only a few square centimeters with the same light source. This makes this technology very versatile to build terabit point-to-point backhaul systems as well as ultra-small cell terabit mobile access systems. In the latter, the signals can be piggybacked on existing lighting systems. It has been demonstrated that LiFi can sufficiently and effectively support indoor mobile communication systems including both effective interference management and handover [301]. In addition, sleep mode concepts can be used effectively. For instance, while all the lights may be turned on for illumination, only those luminaires may activate their communication function when there are active users within their coverage area. This will enhance efficiency and at the same time will reduce co-channel interference.

VLC is also a viable solution to enable gigabit wireless networks underwater [302], as shown in Figure 31. This is deemed an important feature within the trends in 6G to extend wireless networking capabilities beyond terrestrial cellular communications.

In terms of FSO systems, wireless optical links are constructed between buildings or satellites, where

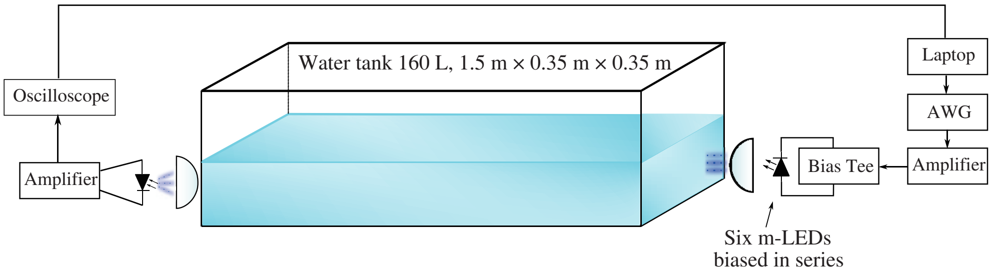

**Figure 31** (Color online) Underwater communication system using micro LEDs [302].

the relative position shifts are small enough to be compensated by conventional acquisition, tracking, and pointing (ATP) subsystems [303, 304]. This means that FSO appears to be an ideal candidate for high-speed backhaul links and fixed meshed optical wireless networks. However, the requirements for ATP systems in high-speed mobile communication scenarios are very challenging. In this case a grid-of-beam and an associated beam-switching approach might be more advantageous. Another promising way to manage high-speed mobility and multi-user access in OWC is the newly proposed optical mobile communication (OMC) technology [290]. OMC uses optical phased arrays to steer optical beams to mobile users with high energy efficiency. Different from conventional ATP subsystems, optical phased array steers the laser beams by changing the optical phases. Specifically, a pixelated liquid crystal spatial light modulator device can be used to adjust the beam phase. This approach introduces more DOF to optical systems and makes the optical elements work in a way which is comparable to massive MIMO systems. Through optical phased array, the original beam can be split with adjustable power allocations and redirected to multiple users at different locations, making it possible to serve a large number of mobile terminals.

##### 4.2.5 *Channel measurements and models for 5G and beyond*

Currently there are nine standard 5G channel models, i.e., COST 2100 [272], MiWEBA [305], QuaDRiGa [306,307], METIS [308], 5GCM, mmMAGIC [309], 3GPP TR 38.901 [274], IEEE 802.11ay [310], and IMT-2020 [311] channel models. They are used to model various channel characteristics, including massive MIMO, dual/high mobility, 3D, mmWave, spatial consistency, blockage, atmosphere attenuation, etc. However, none of them can fully capture all the mentioned channel characteristics [312].

Many channel models have been proposed by using different channel modeling methods to accurately describe the underlying channels. Here we briefly introduce channel measurements and models for 5G and beyond, including mmWave, THz, optical, satellite, UAV, maritime, underwater acoustic, HST, V2V, ultra-massive MIMO, orbital angular momentum (OAM), and industry IoT communication channels.

mmWave and THz channels show similar channel characteristics, such as large bandwidth, high directivity, large path loss, blockage effects, atmosphere absorption, and more diffuse scattering. THz bands show more severe path loss, atmosphere absorption, and diffuse scattering than mmWave bands. mmWave channel has been well studied at 26/28, 32, 38/39, 60, and 73 GHz bands [313, 314]. Even though, mmWave channel measurements with MIMO antennas, high dynamics, and outdoor environments are still required [315, 316]. For THz channels, most of the channel measurements are around 300 GHz band [317–319]. The channel characteristics above 300 GHz are still not clear and need extensive channel measurements in the future. The deterministic mmWave channel models include ray tracing, map-based, and point cloud models. The ray tracing model is applied to IEEE 802.11ad, while the map-based model is applied to METIS. The quasi-deterministic (Q-D) model is used in MiWEBA and IEEE 802.11ay. The stochastic models include Saleh-Valenzuela (S-V), propagation graph, and GBSM. GBSM is used in several standardized channel models, such as NYU WIRELESS, 3GPP 38.901, METIS, and mmMAGIC. The ray tracing model and GBSM are also widely used in THz channel modeling. Meanwhile, human/vegetation blockage and rain/cloud/snow/fog attenuations also need to be modeled for mmWave/THz channels.

Optical wireless channels exhibit some unique channel characteristics, such as complex scattering properties for different materials, non-linear photoelectric characteristics at Tx/Rx ends, background noise effects, etc. The channel scenarios can be further classified as directed LOS, non-directed LOS, non-directed non-LOS (NLOS), tracked, etc. [320]. The optical wireless channel has no multipath fading,

Doppler effects, and bandwidth regulation. The measured channel parameters include channel impulse response (CIR), path loss, shadowing fading, root mean square (RMS) delay spread, etc. The proposed deterministic models include the recursive model, iterative model, DUSTIN algorithm, ceiling bounce model, and geometry based deterministic model. The proposed stochastic models are classified as GBSM and non-GBSM.

Satellite communication orbits can be classified as geosynchronous orbit and non-geostationary orbit. The usually applied frequency bands for satellite communications are Ku (12–18 GHz), K (18–26.5 GHz), Ka (26.5–40 GHz), and V (40–75 GHz) bands. The channel is largely affected by weather dynamics, including rain, cloud, fog, snow, etc. [321]. Rain is the major source of attenuation, especially at frequency bands above 10 GHz. Besides, satellite communication channel shows extremely large Doppler frequency shift and Doppler spread, frequency dependence, large coverage range, long communication distance, etc. As satellite communication channel is mainly LOS transmission, the received signal is stable in general, except the effects of weather condition and tropospheric scintillation. The received signal amplitude can be modeled by Markov-chains. Meanwhile, some preliminary studies try to use GBSM to model the satellite channel [322].

The UAV channel shows unique channel characteristics, such as 3D deployment, high mobility, spatial and temporal non-stationarity, and airframe shadowing [323, 324]. In general, UAV channel can be classified as air-to-air and air-to-ground channels. Both narrowband and wideband channel measurements have been conducted, most of which are at 2, 2.4, and 5.8 GHz bands. The measured environments include urban, suburban, rural, and open field. The measured channel parameters include path loss, shadowing fading, RMS delay spread, K-factor, amplitude probability density function (PDF), etc. A comprehensive summary of air-to-ground large-scale path models was given in [323]. UAV small-scale channel models include deterministic and stochastic ones. The deterministic models include ray tracing and analytical models such as two-ray model. The stochastic models include the GBSM and Markov model.

Maritime communication channels mainly include air-to-sea and near-sea-surface channels [325]. For air-to-sea channel, the UAV or relay is used as the BS to communicate with ships on the sea surface. This type of channels is also named as the UAV-to-ship channel. For near-sea-surface channel, a ship can communicate with other ships (ship-to-ship) or fixed BS near the sea (ship-to-land). The unique features of the maritime propagation environment cause sparse scattering, sea wave movement, ducting effect over the sea surface, time non-stationarity, long communication distances, and climate factors. Maritime channel measurements are conducted at 2.4 GHz and 5.8 GHz bands with maximum distances up to 10 km. The path loss is shown to be different for ship-to-ship, ship-to-land, moving, and drifting scenarios. The underwater channel is prone to multipath propagation owing to refraction, reflection, and scattering. It disperses in both time and frequency domain, which leads to the time-varying and Doppler effects. Channel measurements are unusually conducted at several kHz, ranging from 2 kHz to 32 kHz. Ray tracing can be used as a deterministic simulation method for maritime channel and underwater acoustic channel. Apart from it, the two-ray model and three-way model are also used in practice. Stochastic models include GBSM and two wave with diffusion power. Rayleigh, Ricean, and log-normal distributions are usually used for underwater acoustic channel.

The HST channel has problems such as frequent and fast handover and large Doppler spread. mmWave/THz and massive MIMO are potential key technologies to be utilized in HST communication systems. Some preliminary channel measurements have been conducted in open space, hilly terrain, viaduct, tunnels, cutting, stations, and intra-wagon scenarios [326]. Vehicular network is a typical industry vertical application of 5G/6G for uRLLC scenario. V2V channel at sub-6 GHz band has been widely investigated, while mmWave V2V channel needs more measurements. A survey of current mmWave V2V channel measurements was given in [327]. In summary, V2V channels were measured at 28, 38, 60, 73, and 77 GHz bands. All of them are configured with single antenna at both sides of Tx and Rx, and either the Tx or the Rx can be fixed or moving. The measured environments include highway, urban street, open area, university campus, parking lot, etc. mmWave V2V MIMO or even massive MIMO channel measurements with high mobility are promising in the future. For HST and V2V channels, the high mobility and non-stationarity need to be considered. Ray tracing can be used to simulate the HST/V2V channel. Stochastic channel models include the GBSM, QuaDRiGa-based model, dynamic model, Markov model, and propagation graph model.

Ultra-massive MIMO utilizes thousands of antennas to largely improve the spectral and energy efficiency, throughput, robustness, and DOF of wireless communication systems. It can be combined with other key technologies, such as mmWave/THz, V2V, and HST communications. Owing to the use of large antenna arrays, the channel shows spherical wavefront, spatial non-stationarity, and channel hardening properties, which have been validated by previous massive MIMO channel measurements at sub-6 GHz/mmWave bands in indoor and outdoor environments. At sub-6 GHz band, the dimension of the massive MIMO array can be several meters. At THz band, owing to the developments of plasmonic nanoantenna arrays, it is possible to realize ultra-massive MIMO up to $1024\times1024$ [328]. For massive MIMO channel, the spherical wavefront, non-stationarity, and cluster appearance and disappearance properties need to be considered. In general, the spherical wavefront can be modeled in GBSM with accurate propagation distance calculation for each individual antenna element. The non-stationarity is usually modeled by the concept of the visible region and the cluster birth-death process.

OAM represents electron rotation around the propagation axis generated by the energy flow [329]. OAM based on communications can be obtained from traditional MIMO theory under certain conditions. However, beam divergence and misalignment will severely decrease the transmission distance of OAM waves. Moreover, reflection will destroy the orthogonality of OAM waves, thus degrading the performance in NLOS scenario. Up to now, there are only very limited channel measurements to verify the feasibility of OAM in different scenarios and no realistic OAM channel models are available. The current researches focus on OAM wave generation/detection, antenna design, and the discussion of OAM potentials in wireless communications. The limited OAM channel analysis results mainly aim to verify the feasibility of OAM in different scenarios. Channel modeling for OAM wave propagation is still an open issue.

The industry IoT channel exhibits many new channel characteristics, such as varied path loss, random fluctuations, NLOS propagation, large amounts of scatterers, and multi-mobility [330]. Only a few channel measurements have been conducted in industry IoT environments. These measurements are mainly at sub-6 GHz band as in current IoT standards. However, channel measurements at mmWave bands are also promising in industry IoT environments for future massive connections with high transmission data rates. In [330], different path loss channel models were compared for industry IoT channels, including the free space path loss model, single-slope model, 3GPP models (RMa, UMa, UMi, InH), industry indoor model, and overall path loss model.

Wireless channels are the foundation of any new communication systems, including the future 6G wireless communication networks. As the 6G channel encounters wide frequency bands and complex scenarios, it causes many new problems and needs to be studied in the future [331, 332].

- 6G wireless channel measurements. Channel measurement is the direct way to study any new wireless channels. Current channel sounders are limited in their performance to fully satisfy the demands of channel measurements, such as the frequency band, signal bandwidth, number of RF channels, system dynamic range, and measurement speed. Channel sounders are expensive especially for high frequency bands. Channel measurement is also time consuming and even impossible for extreme environments. How to design a high-performance channel sounder which can fulfill the requirements of future 6G channels is urgent. Meanwhile, the measured data should be processed by high resolution channel parameter estimation algorithms [333].
- Channel measurements and models for intelligent reflection surface (IRS) based 6G technologies. IRS is a recently proposed concept beyond massive MIMO where future man-made structures are electronically active with integrated electronics and wireless communication making the entire environment intelligent [334]. IRS can be implemented with ultra-massive antenna arrays and controlled by reconfigurable processing networks with the aid of AI and ML. As the wireless channel becomes intelligent and reconfigurable, IRS shows great potential to satisfy the future 6G demands. Channel measurements and modeling for IRS based 6G technologies are indispensable to validate and evaluate the performance of IRS, but they are largely open issues in the current research.
- AI enabled channel measurements and models. Some preliminary studies have shown the potential of AI and ML based wireless channel measurements and models, for example, MPCs clustering, scenario classification, and channel prediction, by using clustering, classification, and regression algorithms [335]. Different ML algorithms, such as artificial neural network (ANN), convolutional neural network (CNN), and generative adversarial network can be applied to wireless channel modeling [336]. One of the benefits of applying AI and ML over traditional channel modeling methods is that they can partly predict wireless channel properties for unknown scenarios, unknown frequency bands, and future time instants.
- A general standard 6G channel model framework. In the previous communication systems, the evolution of standard channel models showed a clear trend. In second generation, the channel model was for narrowband single-input single-output (SISO) channel. In addition, only 2D azimuth angle and time domain were considered with wide sense stationary assumption. In third generation, the channel model dealt with wideband SISO channel and evolved to time-delay domain. In 4G, the channel model extended to wideband MIMO and further evolved to 3D (azimuth and elevation angles) and space-time-delay domain. In 5G, the channel model extended to ultra-wideband massive MIMO. The channel was fully characterized in 3D, non-stationary, and space-time-delay domain. The frequency bands also increased from sub-6 GHz to mmWave bands. Meanwhile, the scenarios have also been much more complicated. The standardized 5G channel models prefer to use a general channel model framework with different parameter sets for different scenarios. In [337], a general 3D non-stationary 5G channel model was proposed to cover the four challenging scenarios, i.e., massive MIMO, HST, V2V, and mmWave. However, all of the existing standard channel models only concentrated on terrestrial communication networks and frequencies up to mmWave bands, while 6G channels should cover space-air-ground-sea integrated networks and frequencies up to optical frequency bands. It will be more challenging to derive a general 6G channel model framework. As 6G wireless channels become heterogeneous and show different scales over the wavelengths, how to describe 6G wireless channels with a general standard channel model framework is an open issue which needs careful investigations.

- The complex relationship among channel model parameters, channel characteristics, and system performance. The channel model parameters include large-scale parameters (path loss and shadowing) and small-scale parameters (multipath amplitude, delay, angles, Doppler shift, etc.). Channel characteristics include time autocorrelation function, spatial cross-correlation function, RMS delay spread, RMS angle spread, Doppler power spectral density (PSD), stationary interval, etc. System performance uses channel capacity, BER, energy efficiency, and spectral efficiency as the normal metrics. It is well known that channel model parameters will affect the channel characteristics as well as the system performance. However, it is a complex non-linear relationship and needs more investigations in the future.
- The performance evaluation of 6G channel models. Accuracy, complexity, and generality are three metrics to evaluate the performance of 6G channel models. The accuracy of a channel model is measured by comparison with measurement results or its effect on system performance. Complexity is measured by the number of channel parameters, computation operations, and simulation time. Generality of a channel model is that whether it can be applied to multi-frequency bands and multi-scenarios by adjusting channel model parameters. A good channel model should be a trade-off among the three metrics, while the theory of such trade-off is still an open issue.

#### 4.3 Full applications: AI enabled wireless networks

With the rapid progress in AI and ML, it is expected that 6G networks will have much higher intelligence than their predecessors. The intelligence would cover both operations and services.

With given resources, the optimization of network operation is typically a multi-objective performance optimization problem subject to a series of complex constraints. Network resources include node and device ability, available spectrum, computing power, energy, and communications channels. It would be desirable to meet multiple performance requirements with minimum precious resources such as energy. These multi-objective performance optimization problems are usually non-deterministic polynomial (NP)-hard, and in situ optimization is hard to realize. With the development of ML techniques, especially DL, however, BSs equipped with dedicated AI chips could carry out relevant learnings to allocate resources efficiently to achieve performance close to the optimum.

##### 4.3.1 *AI and ML technologies: an overview*

The emerging AI and ML are powerful and scalable to improve the quality of the next-generation wireless network. Mobile big data (MBD) [338, 339] is a new research area combining mobile computing and big data, which poses great challenges on the source, analytics, applications, characteristics, and security. One of the core advantages of AI and ML lies in their data-driven characteristics [340]. According to [341], most scenarios are hard to develop exact mathematical models in 5G network. AI and ML methods improve the efficiency and latency of network by learning features from massive data rather than pre-established fixed rules. Besides, the next-generation wireless networks tend to evolve into complex systems and the service requirements vary in different applications and networks [342]. The adaptive and predictive AI and ML algorithms can build self-aware and smart networks. The taxonomy of ANNs is listed in [343]. The massive data exchanged between the device and server can be processed by both recurrent ANNs and feed-forward ANNs.

The AI and ML are applicable for various network layers [344] owing to their powerful capabilities. Adaptive BS, proactive caching, and resource allocation are the three crucial applications of AI and ML techniques [345] other than big data. The ultra-dense future wireless network increases the energy consumption as well as data traffic. AI and ML methods can help to improve the energy efficiency by developing more efficient allocating and scheduling. ML methods can also be applied to optimize the physical layer of wireless networks. The conventional model-based methods cannot handle some complex and unknown channels and meet the higher requirements of next-generation wireless systems. Potential opportunities of ML can be utilized to redesign current BB system, such as decoding and detection modules.

The AI algorithms can be applied to solve various kinds of problems including sensing, mining, prediction, and reasoning, which all contribute to enhancing the 5G cellular networks [346]. For example, AI makes it possible to sense the variations in network traffic, resource utilization, user demand, and possible threats, and further makes it possible to smartly coordinate UEs, BSs, and network entities [346–348]. In this subsection, AI-based wireless communication network architectures are introduced, which involves applications in all layers of wireless networks.

##### 4.3.2 *Physical layer applications*

With the fast growth of wireless communications, the requirements on both the spectral efficiency and energy efficiency will advance beyond 5G. Different from 5G, the physical layer will become more and more complicated in the new era, which poses many new challenges [349,350]. First, it is hard to precisely describe a communication system by a mathematical model because it is too complicated and with many practical imperfections. Second, in order to achieve the global optimality, the barriers between different physical layer blocks must be removed through cooperation. Third, the drastically increasing hardware complexity to address new performance challenges requires new implementation methods to make the algorithms more practical.

In order to deal with the aforementioned challenges, AI and ML have been taken into consideration. On one hand, purely based on the training over MBD, AI and ML can help to optimize the physical layer of communication systems. On the other hand, the construction of traditional communication systems usually relies on expertise. AI and ML can also help address the problems which could not be modeled or exactly solved.

For the data-driven option, there have been many related work. In [351], DL was employed for channel estimation and signal detection in OFDM systems. A DL based pilot design was proposed in [352] for channel estimation of multi-user distributed massive MIMO systems. In [353], deep neural networks (DNNs) were considered for MIMO detection. In [354], DL was used for the decodability detection and early stopping for polar code decoding. In [355], a DL aided SCMA was developed to construct a codebook minimizing the BER and learn an efficient decoding strategy. Though data-driven learning can help address some challenges and is robust, it will suffer from high complexity, especially for a large-scale system [349].

For the model-driven option, the DL approach can exploit both expertise and big data to optimize the system [356]. The complexity can be significantly reduced because the prior knowledge can remove some unnecessary learning efforts. Successful applications of this model-driven DL have been reported in literature. In [357], DL was combined with expert knowledge to generate an OFDM receiver. A learned denoising-based approximate message passing network for beamspace mmWave massive MIMO systems was proposed by [358]. In general, many physical layer modules can be addressed by Bayesian optimal estimators implemented by iterative approaches. DL can also be combined with unfolded iterative estimation process to learn the required parameters, as shown in Figure 32. This approach has found its applications in massive MIMO detection [359,360] and polar decoding [361]. The corresponding hardware implementation has also been studied [362].

As an extension of model-driven AI and DL, an interactive learning design paradigm (ILDP) recently is proposed to make full use of domain knowledge of wireless communications and adaptive learning ability of AI and DL [363, 364]. In contrast to the conventional model-driven approach that follows a non-interactive paradigm, the ILDP consists of communication model module and adaptive learning module, which work in an interactive manner, thus is able to extract useful information in real-time and sufficiently adapt to the ever-changing environments. The ILDP has been implemented to design efficient beam training and tracking for multi-user mmWave communications, fulfilled with DRL [363] and bandit

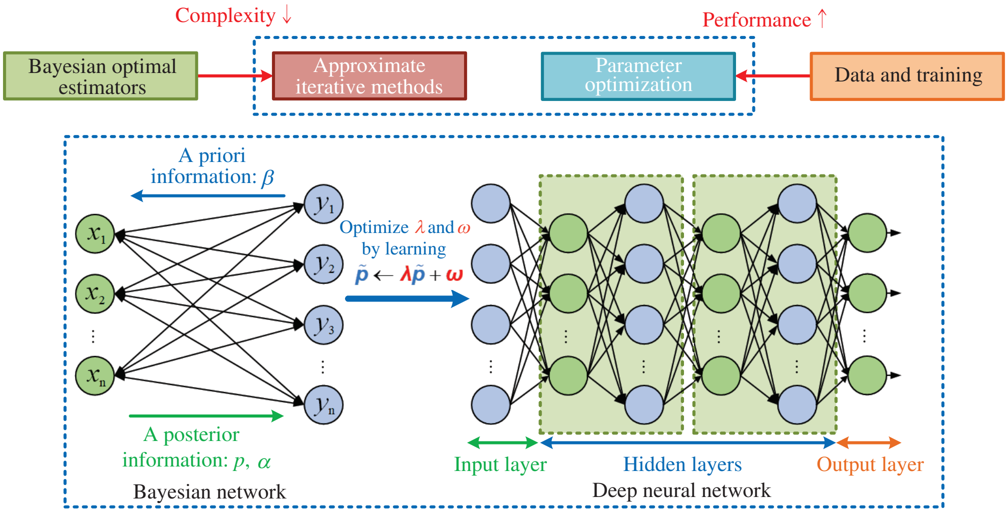

**Figure 32** (Color online) DL-aided Bayesian optimal estimators for physical layer communications.

learning [364]. The results show that they can capture dynamic spatial patterns and adjust beam training strategy intelligently, without knowing priori information about dynamic channel modeling. The ILDP is powerful in intelligently sensing the dynamic environment of wireless communications and has a wide application that is not limited to physical layer.

In addition to physical layer modules, DL can also be applied to design end-to-end systems. In [363], DL was used to generate an end-to-end system, which includes all signal-related functionalities. In [364], a new algorithm was developed to design an end-to-end system even when the channel model is unknown. This functionality is achieved by iteratively training both the Tx and the Rx with true gradient and approximated gradient, respectively. As indicated in [350], the DL-aided iterative Bayesian solver can also be employed for the end-to-end system. The resulting system has advantages in implementation efficiency because all physical layer functionalities can be uniformly implemented with the same hardware accelerator, which is reconfigurable and scalable for different applications. In order to deploy the DL-based approaches, efficient hardware implementation is essential to bridge the gap from theory to practice.

Existing researches have shown the advantages of applying AI and DL to physical layer communications. Thanks to the power of DL, the issues that could not be addressed by traditional approaches can be well taken care of. However, because this research topic is still in its infancy, there are many issues remaining for the future research [365]. First, we need to identify whether DL will outperform the traditional approaches, or they have their own application areas. Second, we have to balance the training efforts of performance of DL-based physical layer communications. Third, we need to figure out the proper learning metric for communication systems. Fourth, it is important to generate sufficient and valid MBD for learning. Last, in order to make the DL-based approaches applicable, efficient hardware implementation is essential to bridge the gap between theory and practice.

#### 4.3.3 *Upper layer applications*

Through learning features from the big data generated by wireless network's infrastructure and sensor devices, network configurations can be optimized, resulting in better network performance. AI aided network traffic, and performance prediction and control have been studied for cellular networks.

Because the customers' demand for quality and quantity of the data traffic increases immensely, the network traffic control in 5G scenario becomes a critical issue. For the 5G UDNs traffic management, a long short-term memory resource control algorithm was adopted in [366] to predict the traffic load at BSs. The resource action policy is adjusted to avoid congestion based on the prediction results to obtain lower packet loss rates, and higher throughput and mean opinion scores. Other applications of the wireless traffic prediction model for the 5G traffic management under the eMBB, mMTC, and uRLLC scenarios have been discussed in [367]. Supervised learning, unsupervised learning, and DRL algorithms could be applied to traffic management. Distributed and efficient ML algorithms for optimizing the uplink traffic in 5G mMTC and uRLLC scenarios have also been discussed. Furthermore, an AI assistant content

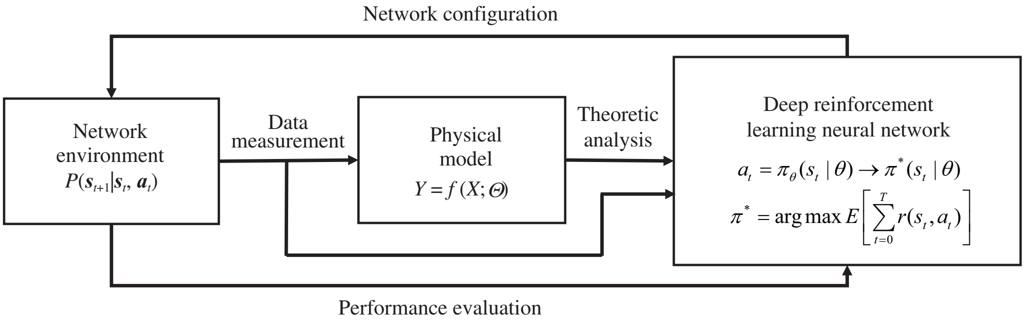

**Figure 33** The framework of 5G network optimization based on DRL with the help of physical model.

retrieval algorithm framework was proposed in [367] for optimizing the data traffic.

ML techniques can help optimize and manage the mobile networks and reduce the operational costs by performance prediction. In [368], prediction tasks were first categorized, and then related ML methods, such as Gaussian process regression, exponential smoothing of time series, and random forest, were used to demonstrate satisfying prediction results for the real-world data set, and to reduce the operational costs for the management of wireless mobile networks. In [369], the SINR, inter-carrier interference (ICI), modulation coding scheme (MCS), and transmit power were used to characterize performance, predict optimal radio parameters, and enhance network performance. In [370], the user traffic was exploited to predict the mobile traffic demand and provide better resource allocation. Recently, federated learning has also been considered for wireless edge networks and has drawn increasing interests from both academia and industry. A comprehensive survey of this specific topic can be found in [371].

One critical challenge in future 5G and 6G networks with massive MIMO is the introduction of interference management based on beamspace transmissions. In the beamspace domain, the geometrical information, e.g., user position, becomes significantly important for performance optimization. However, the large number of beams and adjacent cells, along with the complex relationship between beamspace channel and BS parameters, e.g., tilt angle, all make the interference modeling and consequent performance prediction/optimization very difficult. Therefore, by exploiting the large amount of historical beamspace channel information, ML tools can model an approximate function between the BS parameters and beamspace channel information at any geometrical location of an interested scenario or even the straightforward performance metric. Several learning frameworks have been proposed to solve the above problems. The common key problem is to compress beamspace channel information, which highly affects the complexity of learning network.

A typical framework of 5G network optimization based on DRL with the help of physical model is given in Figure 33. The network controller observes the network state ($\boldsymbol{s}_t$). Then the controller decides the strategy of network configuration ($\boldsymbol{a}_t$), such as beam pattern and power control, via neural network according to the network state. $\pi_\theta$ represents the network with parameter $\theta$ and $\pi^*$ represents the optimal policy, i.e., maximizing the accumulated performance gain. The probability of state transition $P(\boldsymbol{s}_{t+1}|\boldsymbol{s}_t, \boldsymbol{a}_t)$ is dependent on the network environment, which is generally unknown to controller. After network configuration, the network environment feeds back the performance gain ($r$), such as coverage and interference level, which can be used to train the DRL neural network. Besides, to further improve the capacity of DRL, the framework introduces a reasonable 5G physical model based on careful analysis on measurement data and reasonable assumptions. The physical model can possibly expand the network state or supervise the training process of the neural network. The efficiency and effectiveness of DRL neural network are expected to be improved for further applications.

#### 4.3.4 *Resource allocation applications*

One important task of resource allocation is to predict users' future service requests, location and mobility, and the network conditions in 5G cellular networks. This helps the on-demand resource orchestration process to properly balance the resources, guarantee high resource efficiency, and reduce network cost by avoiding overision. For example, the processing resources of the central unit can be properly configured to achieve more pooling gain according to the predicted traffic fluctuation. In addition, some parts of access resources can be turned off if no traffic is predicted in the corresponding coverage area. AI has great potential in these areas. A general framework of DL-based resource management is illustrated in

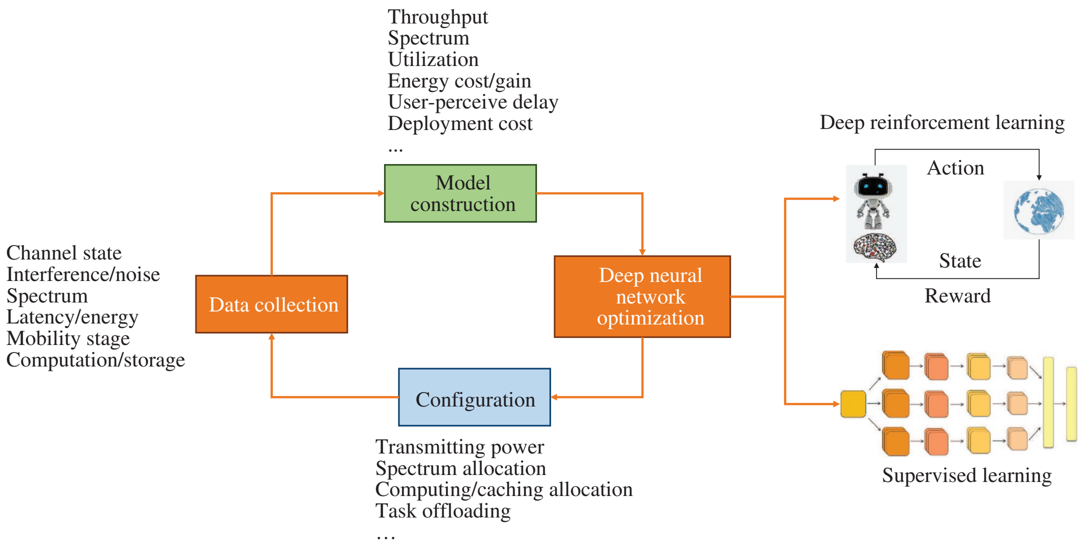

**Figure 34** (Color online) An illustration of virtual vehicular networks.

Figure 34, which consists of data collection, model construction, DNN optimization and configuration.

AI can help to allocate radio resources efficiently. An important example of AI application in resource allocation is the intelligent vehicular networks. The vehicular network brings unprecedented challenges unseen in traditional wireless communications systems owing to its strict and diverse QoS requirements as well as the inherent dynamics in vehicular environments, such as fast time-varying wireless channels and network topology. How to dynamically allocate the resources for ITS to maximize the QoS for end users with small provisioning overhead is a nontrivial task. AI provides a powerful tool to tackle these problems [372]. The key motivation of using AI for resource allocation in vehicular networks was discussed in [373]. According to [373], DRL approach can address resource allocation problems in ITS that are difficult to handle in the traditional optimization framework.

With the development of recent innovative applications, such as AR and self-driving, more and more computation-intensive and data-intensive tasks are delay-sensitive. AI can also help improve these applications by using computing, caching, and communication wisely. MEC in UDN is expected as an effective solution for meeting the low latency demand. However, the distributed computing resource in edge cloud and energy dynamics in the battery of mobile devices make it challenging to offload tasks for users. To tackle the dilemma, a collaborative and on-demand DNN co-inference framework, named "Edgent" was developed in [374]. Its core ideas include: (i) DNN partitioning that adaptively divides DNN computation between device and edge to leverage hybrid computation resources in proximity for real-time DNN inference. (ii) DNN right-sizing that accelerates DNN inference through early-exit at a proper intermediate DNN layer in order to further decrease the computation latency. Furthermore, an intelligent offloading system was constructed in [375] for vehicular edge computing by utilizing DRL. Moreover, to design an efficient resource allocation policy, a DRL with the multi-timescale framework was proposed in [376].

AI technology can be also applied to allocate caching and computing resources. According to [377], AI-based cache-enabled communications are important research directions. A DRL based proactive caching mechanism was developed in [378] for vehicular multi-view 3D video streaming, where clustering technique is used to implement the DRL algorithm working in an action space of variable size. Furthermore, coded caching, which can create multicast opportunities depending on the cumulative memory available at all users, was modified in [379] to make the transmission robust to in-and-out wireless network quality. In this active field of coded caching research, the placement delivery array (PDA) for characterizing the coded caching scheme has attracted vast attention. However, designing PDA has high computational complexity. In [380], a deep neural architecture was proposed to learn the construction of PDAs to reduce the computational complexity. The problem of variable size of PDAs is solved using neural attention and reinforcement learning. Moreover, a DRL method was developed in [381] to reduce the complexity of an

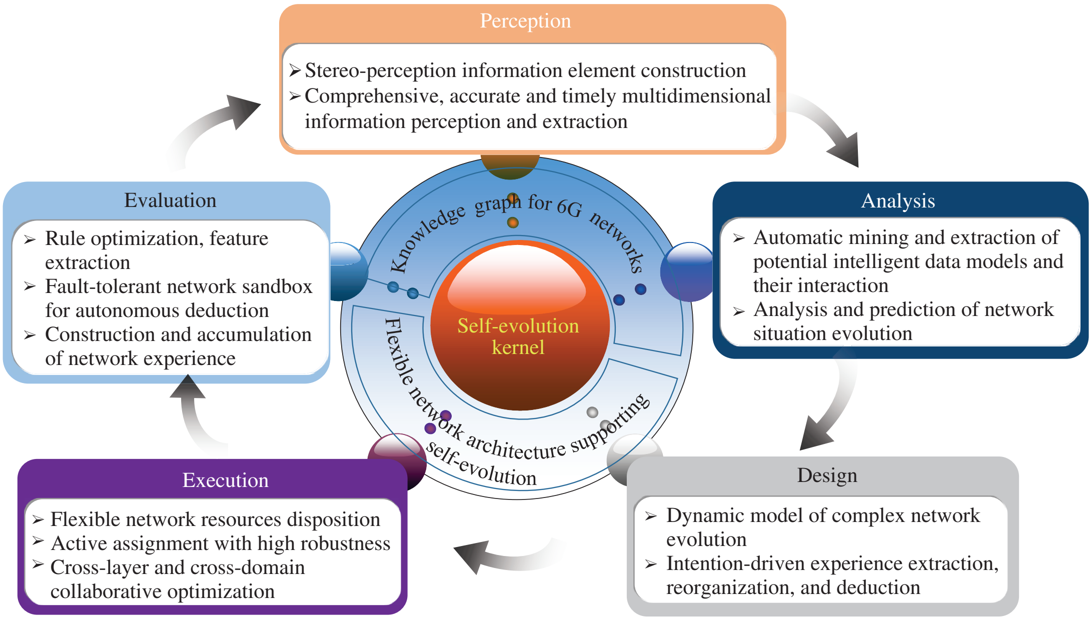

**Figure 35** (Color online) The self-evolution closed-loop structure of the intelligence-endogenous network.

integrated framework for dynamic orchestration of networking, caching, and computing resources.

As pointed out in [377], AI for network traffic control and resource management in cache-enable networks remains immature, owing to the difficulty in uniquely characterizing the network environment and traffic features subject to an appropriate input and output dataset to the learning structures. We should design an intelligent technique that can predict an environment action event a priori so that the strategy and agents can be adjusted according to the potential action in a proactive manner.

##### 4.3.5 *Intelligence endogenous networks (IENs)*

5G mainly focuses on addressing the urgent needs of specific scenarios and key applications in the vertical industries. After that, how to expand network capacities to achieve high-bandwidth mobile connection for anything, anytime and anywhere, and to further support sensory interconnection, holographic transmission, and digital twin, becomes the new vision of mobile communication network evolution [3]. Facing the service requirements on supporting virtual and physical fusion, immersive, holographic, contextualized, personalized, ubiquitous communication, and the network requirements on the integrations of heterogeneous network technologies and space-air-ground-sea integrated networks [382], the current networks' operation paradigms with rule-based algorithms, limited by rigid preset rules, are difficult to dynamically adapt to continuously changing user needs and network environments. Moreover, network operation experience cannot be effectively accumulated, which limits the continuous improvement of network capabilities. This means that under the present operating paradigms, networks do not have the ability to self-evolve. Any upgrade and improvement must rely on a lot of professional studies, which is unacceptable for 6G networks with unprecedented scale and operation complexity. Adding "intelligence genes" into the network to form intelligence and self-evolution capabilities is an important way to resolve the above problems.

At present, AI technologies, represented by DL and knowledge graph, are developing rapidly. By introducing AI technologies into the network, multi-dimensional subjective and objective knowledge of the network and the related users, services, and environments are characterized, constructed, learned, applied, updated, and fed back. Based on the acquired knowledge, the stereo-perception, decision deducing, and dynamic adjustment of the network can be realized. Thus, the network can automatically change as it needs for whatever new services we want. We refer to such a network as "IEN". The IEN benefits from a self-evolution closed-loop structure, as shown in Figure 35.

Till now, quite a few AI methods have been applied to network researches, but most of them merely use ML algorithms to solve specific network problems [383–385]. However, the IEN is not the result of simply applying AI methods into specific problems, but the practice to design and construct the AI system that fits well into network systems and the corresponding network architecture and operating environment for such a system. The realization of this goal is under the guidance of principles and methods of modern AI, combined with the natural attributes and operation characteristics of the network system.

Taking this direction, the theoretical problems and key technologies needed to be resolved to build an IEN include the following.

- Knowledge graph characterization and construction technology for 6G networks. This is about the mechanisms for characterization, construction, acquisition, governance, and evolution of the multi-dimensional subjective and objective knowledge for 6G networks, including structuring intelligent data models and their interaction models, as well as automatic mining and refining of the potential models. This is the theoretical basis for implementing IEN.
- Stereo-perception technology for the holographic network. This includes the methods for the construction of stereo-perceptional information elements based on knowledge graph, as well as the mechanisms for comprehensive, accurate and timely multi-dimensional information perception, extraction and prediction. These technologies will form good information bases for network decision-making.
- Soft and flexible network resources scheduling mechanism. Based on knowledge graph and multi-dimensional stereo-perceptional information, the research focuses on elastically distributing various types of resources such as computing, routing, and caching, as well as the robust proactive provision and the cross-layer and cross-domain collaborative optimization.
- Network self-evolution mechanism. Research directions include flexible network architecture to support self-evolution, the composition of the network self-evolution kernel, design of the dynamic models characterizing complex network evolution, methods for the intention-driven extraction, recombination, and deduction of network experience.

##### 4.3.6 *Toward ICDT convergence in 6G*

It is anticipated that the ICDT will be more and more converged in the 6G era. This ICDT convergence will accelerate innovation and integration in various aspects of the network design, e.g., the network architecture, the core network, RAN, physical layer, and higher layer protocol stack. Smart, open, green, and soft will be the key characteristics of the 6G networks. As green [386–388], soft [389, 390], and open design of wireless communication networks have been investigated intensively in 5G, in this subsection, we will focus on the introduction of AI to the design of 6G.

The booming of big data technologies and AI in the wireless communication industry has given people abundant imaginations on how smart the future wireless network could become. With powerful data acquisition platforms of operators and over-the-top companies, almost all wireless data can be collected from users in core network, RAN, and the service providers. As have been demonstrated in various applications, with efficient storage management of the collected data, big data analytics [347, 391–397] is capable of processing these data and help obtain the key features of each type of data, and label them with sufficiently fine granularity. In contrast to traditional data processing at the BSs, big data platforms are capable of both predictive and prescriptive analytics with powerful ML techniques, such as support vector machine and DL. For example, reliable prediction on the user position, mobility behavior, and service type can be made. Also, the RAN characteristics can be obtained, such as the channel environment, user density and distribution, adjacent cell interference, spatial and temporal traffic variations. Such predictions enable flexible RAN configurations in stabilizing the wireless connections, making better utilization of the radio resources, proactive network control, or even designing AI enable wireless communication network with flexibly configured network functions, signaling, and processing.

In recent years, there have been intensive studies on applying big data analytics and AI to wireless communications. For example, instead of viewing MBD as an unwanted burden, the authors in [391] proposed methods to capitalize on the vast data traffic for building a big data aware wireless network with better wireless service quality and new mobile applications. The authors of [392] addressed the issues of resource management, cache server deployment, QoE modeling, and monitoring in heterogeneous networks, and proposed solutions in a wireless big data driven framework. It was discussed in [347] how wireless big data can impact wireless communication network design, from the perspectives of network architecture, protocol stack, signaling procedure, and physical layer operations. The authors in [394] further proposed a novel data-driven intelligent RAN architecture that is hierarchical and distributed, which operates in real-time and identifies the required data and respective workflows that facilitate intelligent network optimizations. This design methodology is well reflected in the RAN architecture in the open radio access network (O-RAN) alliance.

Introducing the AI capability into the network design in the industry was started from the core network. A study item on network data analytics (NWDA) was approved in 3GPP [398], which is a network entity defined to provide network analytics to the core network PCF. NWDA is envisioned to identify service characteristics to form a big data model that is then used to classify incoming traffic to allow for customized and improved service delivery. Furthermore, a study item on big data driven network architecture for 5G was already approved in 3GPP service and system aspects working group 2 (SA2). This study item aims to investigate solutions for a big data driven network architecture with information management across all technical domains for context mining. This is truly necessary because the wireless applications may not want to reveal their secrets to the operators, which complicates the operation and management of the wireless networks. However, this study focuses on the core network, thus inherently cannot handle some RAN enhancement efficiently owing to the interaction overhead and process delay. In addition to the overhead and process delay considerations, it worth noting that most of the RAN enhancement greatly depends on the RAN characteristics like the channel environment, user density and distribution, adjacent cell interference, radio resource availability. Thus, for RAN enhancement, it is more appropriate to also include the wireless big data analytics at the local RAN side. The core network may potentially provide some useful context information and base modeling. To support the wireless big data analytics for RAN, it is quite necessary to introduce a logical big data function in the RAN side and consider its interface with different layers of RAN and core network for data/model collection and policy/parameter distribution. This is actively being investigated in the O-RAN alliance.

The physical layer standardization design has always been closely related to the implementations of physical layer transceivers, which has taken the form of a sequential module-by-module realization since the initial commercialized communications, e.g., coding, modulation, waveform, and multiple antennas. Such realization incurs heavy and complicated internal interfaces and control signaling. The emerging AI-enabled physical layer approaches could fundamentally break through the limitations of conventional module-by-module transceiver realizations. As proposed in [399, 400], the physical layer transceivers could be implemented through the advanced ANNs. However, owing to the challenges in data collecting and data size, the current physical layer end-to-end learning results are trained based on simulated data generated by statistical channel models. In fact, it is very challenging for these statistical channel models to exactly characterize all channels in the real radio environments. Therefore, the performance of end-to-end learning cannot be guaranteed when training on simulated data, which promotes the necessity of learning on data from real radio environment.

Being favorable to operators for potentially significant cost reduction, the AI-enabled auto-upgrade of air interface (both physical layer and higher layers) [401] on the white-box infrastructure hardware will inevitably affect the R&D roadmap of infrastructure vendors and there seems much debate ahead in the future standardization. No matter how fast or slow is the standardization process, the AI-enabled design methodology for wireless communication networks will be more and more popular.

### 4.4 Endogenous network security

Because 6G networks are expected to integrate all of the operational networks, network security becomes an important issue. Hence in this subsection, we summarize the recent advances in physical layer security (PLS) and quantum security. Then, the network security issues and possible countermeasures in 6G in the context of different types of networks are discussed.

#### 4.4.1 *Current status and main issues*

In this subsection, we commence with a rudimentary introduction to classical cryptography, PLS, and quantum-domain security systems. Anecdotally, the communicating partners are often referred to as Alice and Bob, representing Tx and Rx or source and destination.

**Classical cryptography.** In a classical cryptosystem, the legitimate Tx generates the so-called plaintext and transmits it to the Rx. To avoid divulging the information, the plaintext is encrypted to generate the so-called ciphertext by using a secret key and a specific encryption algorithm, which can now be transmitted over an insecure public channel. Unless the eavesdropper knows the key, it is unable to decipher the message. Because the Rx knows the secret key, it can regenerate original plaintext using the secret key and his decryption algorithm. Therefore, to prevent the potential eavesdroppers from intercepting the key, it must be transmitted from the Tx to the Rx over a secure channel.

However, the popular so-called "one-time pad" technique requires a secret key, which is at least as long as the plaintext, hence the overhead associated with generating sufficiently long keys is quite substantial. Apart from this huge overhead imposed by the requirement of long secret keys and the requirement of a secure key-distribution channel, another disadvantage of classical cryptography is that it is unable to detect, whether the information has been tempered with by an eavesdropper.

**Physical layer security.** PLS solutions exploit the random nature of wireless channels for generating secret keys used for encrypting confidential messages and a range of other sophisticated techniques for degrading the received signal qualities at the eavesdroppers and other malicious users. They are also capable of supporting secure transmission without a secret key with the aid of sophisticated signal design and signal processing techniques [402, 403].

Let us now briefly touch upon how to extract secret keys from the random time-variant physical-layer channel characteristics of radio propagation. The conceptually simplest scenario is, when the wireless channel may be deemed identical in the uplink and downlink directions between the Tx and Rx nodes. In this context, the FDD and TDD modes have different issues affecting the secret key generation, such as sampling delay in TDD systems and the uncorrelated fading caused by high frequency separation between the uplink and downlink of FDD systems. Similarly, the hardware differences between the uplink and downlink result in imperfect reciprocity. The inter-symbol and inter-cell interference are also different at the uplink and downlink receivers.

Hence it may be safer to avoid the assumption of perfect reciprocity. Naturally, then the channel has to be estimated with the aid of well-established techniques, typically relying on pilot signals. The eavesdropper is typically at a different positioned experiences independent fading. If instead of exploiting the reciprocity of the uplink and downlink channels both the uplink and downlink receivers individually estimate the channel between them, the secret keys cannot be easily stolen by the eavesdropper.

One of the simplest ideas is to rely on the received signal strength (RSS) for secret key generation, which results in a sufficiently high key generation rate for supporting high-rate data transmission, provided that the propagation scenario is sufficiently dynamic. Let us briefly touch upon the four basic processes of a simple TDD design example, where the RSS is typically evaluated both at the uplink and downlink receivers. These RSS values may be processed in three consecutive steps: (1) quantization, which is reminiscent of A/D conversion; (2) information reconciliation; (3) privacy amplification. To elaborate briefly, information reconciliation and privacy amplification are necessary, because the RSS samples of the uplink and downlink receivers tend to slightly deviate, which will result in different quantized RSS sequences, $K_A$ and $K_B$.

**Quantum key distribution and secure direct communications.** In contrast to classic cryptography, where two channels are required, three channels in a quantum key distribution (QKD) system are used, namely an insecure public channel, a quantum channel, and an authenticated public channel [404–406]. A secret key can be agreed by the communicating partners Tx and Rx over the insecure quantum channel by relying on an authenticated public channel. In somewhat simplistic manner one could argue that if an eavesdropper tries to observe the quantum-domain information, his/her tampering with the quantum information destroys the quantum state, which hence collapses back into the classical domain and the information becomes useless. Another benefit of QKD is that any malicious eavesdropping action can be detected. Once the Tx and Rx have agreed upon their choice of secret key, one-time pad based encryption may ensue, which is similar to that of the classic cryptosystems. Hence a QKD-based system also uses an insecure public channel for transmitting the ciphertext after the secret key has been generated by QKD.

There is also a high-security quantum communications protocol, which goes beyond the secret key distribution function of QKD. This is referred to as quantum secure direct communications (QSDC), which is capable of eavesdropping detection. In QSDC, the confidential information is directly transmitted by Tx to Rx over an insecure quantum channel, albeit it also requires an authenticated public channel, which is only required for eavesdropping detection by the legitimate user. This is achieved, e.g., by mapping the confidential information to the spin of an electron onto photons and the legitimate Rx can estimate the BER in order to detect any malicious tampering with the quantum information. Whilst classic cryptography, PLS and QKD relay on a secret key agreement protocol, followed by the actual communication session. By contrast, in QSDC, no secret key is required, no confidential information is divulged even in the presence of an eavesdropper in the quantum channel and it does not rely on explicit data encryption and decryption. Hence it has great potential for secure next-generation communications, once it becomes fully developed.

Under current technical conditions, many problems of network security need to be solved. The current network security issues, the network security concerns in 6G and the possible countermeasures to the security and privacy issues are summarized in Subsections 4.4.2 and 4.4.3.

The 5G mobile network with new networking concepts is inherently different from previous mobile generations to provide broadband and customized capability of various novel network services including mobile services, critical industry infrastructures, and vertical society services. These new network features and widespread applications of 5G reveal potential security and privacy challenges.

In conventional networks, the salient functions, such as routing and traffic engineering are typically integrated into specialized hardware relying on bespoke operating system. However, such a rigid architecture may be ill-suited to the dynamic resource demands of state-of-the-art data centers, cloud computing and of the typical heterogeneous network environment. Furthermore, it does not lend itself to flexible upgrade to new innovative functions. Softwarization with advanced technologies, including SDN, NFV, network slicing, and MEC, makes 5G a major shift from traditional hardware- and software-based network architectures. The SBA of 5G core network makes the problems of virtualization security prominent. Although SDN poses potential challenges to the network security and privacy protection, it also enables network-wide consistent security mechanisms [382].

In particular, ETSI has specified security and trust guideline for NFV security [407]. Verification identity, integrity checking, and access management among virtual functions are used to establish trust relationship between different entities. Moreover, latest AI and ML techniques can provide rapid and adaptive security and privacy protection for SDN/NFV related issues. As for the network slicing related security issues, isolation of slices and access control of critical and sensitive data in each slice should be guaranteed.

As for the privacy issues in 5G networks, 3GPP service and system aspects security group (SA3), IEEE-P1912, 5G infrastructure public private partnership (5G PPP) security work group, and NGMN 5G security group have provided requirements and specifications for the privacy preserving and protection in the 5G networks. 3GPP defines the requirement of privacy for the subscribers in release 15 [408]. IEEE outlines the privacy architecture for the end user devices [409]. ETSI describes trust models to increase the data privacy in untrusted environments [410].

To ensure the global commercialization of 5G networks and the evolution of telecommunication networks, standardization of 5G security architecture is the key. 3GPP has outlined a six-level security architecture in Release 15 [411]. The 5G security architecture can be divided vertically into Application Stratum, Home Stratum, Serving Stratum, and Transport Stratum. These four stratums are securely isolated. They are interconnected through standardized protocols and can support secure interconnection and interoperability among vendors. Overall, the global industry should work together to address security risks of architecture, technology, and commercialization with a standard 5G security architecture.

##### 4.4.2 *Network security concerns in 6G*

Although 6G networks face a proliferation of mobile devices and increasingly abundant applications, human-centric mobile communication to achieve customized services is still the core of 6G. With the exploitation of new enabling communication techniques, potential security and privacy issues in 6G networks mainly lies in following aspects.

**Security and privacy problems raised by AI.** AI raises concerns about security and privacy problems, including data security, AI model and algorithm security, vulnerabilities in AI software systems and frameworks, and malicious utilization of AI technologies.

For data security, service providers need to collect an enormous amount of data to train the AI model, however, the collected data may contain the user's sensitive information, such as user identity, trajectory, and location. The sensitive information may be leaked with high probability during data transmission and processing. For AI model and algorithm security, the attacker can get AI model through white box attacks. Even if the architecture and parameters of AI model are unknown, the attacker can still approach the model through checking input and output, further find the attack points and disadvantages of the AI model. In this situation, the dynamic protection of AI model such as monitoring the data flow and the output of the model is critical[5].

5) https://blog.csdn.net/feibabeibei_beibei/article/details/102766347.

The complexity of the implementation of AI systems leads to certain vulnerabilities in AI software systems and frameworks. For example, Google's DL system TensorFlow has some traditional, basic network security problems. After constructing a special model file and entering the DL framework, it can control the entire AI system. The harm caused by these vulnerabilities can lead to denial of service attacks on DL applications, control flow hijacking, classification escape, and potential data pollution attacks [412].

**Security in IoT networks.** The access and management of large-scale, low-power IoT devices can easily cause a signaling storm. Efficient authentication mechanisms are required to support massive IoT deployments. Because IoT devices interact frequently, in terms of interconnection, a unified security standard should be established to prevent attackers from using interconnection protocols for malicious operations.

Traditional encryption and decryption security mechanisms rely on key management systems and computing resources. The security capability of IoT devices is restricted by their limited computing capability, storage space, and battery energy. Therefore, it is necessary to design a lightweight security mechanism to support energy-efficient access of low-power IoT devices. On the other hand, massive device interconnection has expanded the attack surface of IoT networks. Therefore, 6G should strengthen the security protection of network infrastructure and the distributed defense mechanism towards massive IoT devices.

**Security in UAV networks.** Owing to the strict requirements of power and weight, UAVs are not able to support complex cryptographic algorithms like terrestrial base stations. As a result, UAVs are more prone to security vulnerabilities. Lightweight encryption protocols should be designed for UAVs. Considering the working mode of UAVs, the UAVs may fall into the hands of the attacker. Therefore, UAVs should support internal attack protection mechanisms, where physical incursion cannot permit access to the internal functions of the operation system to prevent physical tampering [413].

##### 4.4.3 *Possible countermeasures to the security and privacy issues in 6G networks*

To address the above security and privacy challenges in the 6G networks, advanced security and privacy technologies should be developed.

**Traditional cryptographic methods with much more lightweight algorithms.** Traditional computation complexity-based cryptographic mechanisms such as encryption, authentication, authorization, signature, and privacy-preserving, will still be the major way in safeguarding the network security and data privacy. However, the boosting wireless connections, the prosperity of various delay-sensitive traffics as well as limitations of end-devices in energy, processing capability, storage, and hardware in 6G require more efficient and low-cost solutions for the security and privacy. Lightweight and flexible solutions such as group-based authentication and anonymous service-oriented authentication strategies [414, 415] will be the main research and development direction of the cryptography-based security and privacy.

**Physical layer-based methods.** PLS has been initialized since 1949 [416] and developed since 1975 [417], and PLS is expected to provide another layer of defense besides upper layer cryptographic techniques in the 6G networks [9, 415, 418]. Take advantages of advanced technologies such as advanced channel coding, massive MIMO, and THz, PLS techniques can provide secure transmissions of the wireless signals. Moreover, physical layer secret key and physical layer authentication can provide lightweight secure protections for the air interface. To simultaneously satisfy the requirements (such as latency, overhead, reliability, and throughput) in the 6G networks, more efficient and robust PLS techniques should be developed [415].

**Quantum-based methods.** Quantum-based techniques, including quantum communication and quantum computing, can provide much higher processing capability, more lightweight, and absolute randomness compared with binary-based techniques. Quantum cryptography, including pre- and post-quantum cryptography, is seen as a strong solution to the data security and privacy in the 6G networks [418–420]. QKD system generates keys based on the uncertainty and irreproducibility of the quantum state, and implements key distribution randomly, which thus can provide unconditional guarantee of the security of keys [421]. The QKD system is very expected to be applied in the various 6G networks such as satellite networks [422], optical networks, and oceanic networks [423].

**Blockchain-based methods.** Traditional centralized authentication structure is faced great pressure and risk because of the increasing demand of access equipment and more frequent access authentication requests. Blockchain technology, which was proposed in 2008 [135] and has been widely studied in academia and industry, is seen as a promising solution to the data security and privacy in the 6G networks [424–427]. As a decentralized data structure, blockchain has stronger anti-corruption ability and recovery ability, so it is conducive to the safe operation of the authentication system [425]. As a result, the pressure of data transmission and processing is significantly and the authentication efficiency is improved [426]. With the application of cryptographic algorithms such as group signature and ring signature in the data structure, the data has high anonymity [428, 429].

**AI-based methods.** AI technology, especially ML and big data analytics technology, has been widely used to optimize the configurations of heterogeneous networks in specific scenarios [430]. However, the high complexity of AI technology limits its applications in wireless networks [195]. With the increase of the peak rate of 6G network, the processing capacity of 6G network elements will be greatly developed, which will promote the deep integration of AI and 6G network security design [431], especially for encoding, authentication and anomaly detection [418]. The ML-based auto-encoder can effectively detect the abnormal traffic data in 6G network through reconstruction error [432]. To achieve highly reliable and robust authentication in 6G networks, ML based physical layer authentication has been proposed to mitigate the effect of channel time-varying characteristics on physical layer attributes [433]. Big data analytics can be used to process the exponential growth of 6G network data, and provide the detection application for abnormal user detection [434].

### 5 Conclusion

In this comprehensive survey review, a vision, enabling technologies, four new paradigm shifts, and future research directions of 6G wireless communication networks have been proposed, after having pointed out the limitations of current 5G wireless communication networks. New performance metrics and application scenarios of 6G have been introduced, e.g., to provide global coverage, enhanced spectral/energy/cost efficiency, better intelligence level, security and resilience, etc. In addition, example industry verticals of 6G wireless communication networks have been given, with cloud VR, IoT industry automation, C-V2X, digital twin body area network, and energy efficient wireless network control and federated learning systems as illustrations. The enabling technologies to meet the 6G requirements have been provided. Novel air interface and transmission technologies have been investigated, such as new waveforms, multiple access, channel coding, CF massive MIMO, dynamic and intelligent spectrum sharing and accessing, blockchain-based wireless accessing and networking, photonics defined radio, and MCo for uRLLC. Also, novel network architectures have been studied, including SDN/NFV, network slicing, SBA, CSA, $DEN^2$, CF, and cloud/fog/edge computing. The four new paradigm shifts, i.e., global coverage (satellite, UAV, terrestrial, maritime communications), all spectra (sub-6 GHz, mmWave, THz, optical frequency bands), full applications (AI-enabled wireless communications), and strong network security, have been proposed to achieve a fully integrated 6G network. Future research directions have also been pointed out in each part. We believe that this survey review can provide academic researchers and industry experts with sufficient insightful thoughts on the developments of future 6G wireless communication networks.

**Acknowledgements** This work was supported by National Key R&D Program of China (Grant No. 2018YFB1801101), National Natural Science Foundation of China (Grant Nos. 61960206006, 61901109), Frontiers Science Center for Mobile Information Communication and Security, High Level Innovation and Entrepreneurial Research Team Program in Jiangsu, High Level Innovation and Entrepreneurial Talent Introduction Program in Jiangsu, National Postdoctoral Program for Innovative Talents (Grant No. BX20180062), Research Fund of National Mobile Communications Research Laboratory, Southeast University (Grant No. 2020B01), and Fundamental Research Funds for the Central Universities (Grant No. 2242020R30001).

**Open access** This article is licensed under a Creative Commons Attribution 4.0 International License, which permits use, sharing, adaptation, distribution and reproduction in any medium or format, as long as you give appropriate credit to the original author(s) and the source, provide a link to the Creative Commons licence, and indicate if changes were made. The images or other third party material in this article are included in the article's Creative Commons licence, unless indicated otherwise in a credit line to the material. If material is not included in the article's Creative Commons licence and your intended use is not permitted by statutory regulation or exceeds the permitted use, you will need to obtain permission directly from the copyright holder. To view a copy of this licence, visit http://creativecommons.org/licenses/by/4.0/.

### Appendix A

**Table A1** A list of abbreviations

| Abbreviation | Definition | Abbreviation | Definition |
|---|---|---|---|
| 2D | two-dimensional | MBD | mobile big data |
| 3D | three-dimensional | MCS | modulation coding scheme |
| 3GPP | 3rd generation partnership project | MEC | mobile edge computing |
| 4G | fourth generation | MIMO | multiple-input multiple-output |
| 5G | fifth generation | ML | machine learning |
| 5G-ACIA | 5G alliance for connected industries and automation | MLSE | maximum likelihood sequence estimation |
| 5G PPP | 5G infrastructure public private partnership | MMSE | minimum mean squared error |
| 6G | sixth generation | mMTC | massive machine type communications |
| ACK | acknowledgment | mmWave | millimeter wave |
| A-CPI | application-controller plane interface | MPC | multipath component |
| A/D | analog-to-digital | MR | maximum ratio |
| AI | artificial intelligence | MRC | maximum ratio combining |
| AMF | access and mobility management function | MRT | maximum ratio transmission |
| ANN | artificial neural network | MS | mobile station |
| AP | access point | MTC | machine type communication |
| AR | augmented reality | MUST | multi-user superposition transmission |
| ASK | amplitude shift keying | MZM | Mach-Zehnder modulator |
| AUSF | authentication server function | NAS | non-access stratum |
| BB | baseband | NEF | network exposure function |
| BCH | Bose-Chaudhuri-Hocquenghem | NFV | network functions virtualization |
| BCJR | Bahl, Cocke, Jelinek, and Raviv | NFVI | NFV infrastructure |
| BER | bit error rate | NFV M&O | NFV management and orchestration |
| BPF | bandpass filter | NGMN | next-generation mobile network |
| B-RAN | blockchain radio access network | NG-RAN | next-generation radio access network |
| BS | base stations | NLOS | non-line-of-sight |
| BSS | business support system | NOMA | non-orthogonal multiple access |
| CC | central controller | NP | non-deterministic polynomial |
| CF | cell-free | NR | new radio |
| CGM | continuous glucose monitoring | NRF | network function repository function |
| CIR | channel impulse response | NR-V | new radio vehicle |
| CNN | convolutional neural network | NWDA | network data analytics |
| CoMP | coordinated multi-point | OAM | orbital angular momentum |
| COTS | commercial off-the-shelf | OFDM | orthogonal frequency-division multiplexing |
| CPM | continuous phase modulation | OMA | orthogonal multiple access |
| CPU | central processing units | ONF | open networking foundation |
| CR | cognitive radio | O-RAN | open radio access network |
| CRC | cyclic redundancy check | OSS | operations support system |
| CSA | cognitive service architecture | OTFS | orthogonal time frequency space |
| CSI | channel state information | OWC | optical wireless communication |
| CT | computed tomography | P2P | peer-to-peer |
| C-V2X | cellular vehicle to everything | PAPR | peak to average power ratio |
| D/A | digital-to-analog | PCC | parallel concatenated codes |
| DAS | distributed antenna system | PCF | policy control function |
| D-CPI | data-controller plane interface | PDA | placement delivery array |
| $DEN^2$ | deep edge node and network | PDF | probability density function |
| DetNet | deterministic networking | PDP | power delay profile |
| DL | deep learning | PLS | physical layer security |
| DN | data network | PSD | power spectral density |
| DNN | deep neural network | QAM | quadrature amplitude modulation |
| DOF | degree of freedom | Q-D | quasi-deterministic |
| DRL | deep reinforcement learning | QKD | quantum key distribution |

(To be continued on the next page)

(Continued)

| Abbreviation | Definition | Abbreviation | Definition |
|---|---|---|---|
| EAP | extensible authentication protocol | QoE | quality of experience |
| ECG | electrocardiogram | QoS | quality of service |
| EEG | electroencephalogram | QSDC | quantum secure direct communication |
| EIRP | effective isotropic radiated power | RAN | radio access network |
| eMBB | enhanced mobile broadband | RAU | remote antenna unit |
| EMG | electromyogram | RF | radio frequency |
| EMS | element management system | RMS | root mean square |
| ETSI | European telecommunication standards institute | RSC | radio side controller |
| euRLLC | enhanced-uRLLC | RSS | received signal strength |
| EXIT | extrinsic information transfer | RTT | round trip time |
| FBMC | filter bank multi-carrier | Rx | receiver |
| FCC | federal communications commission | RZF | regularized zero-forcing |
| FDD | frequency division duplex | SBA | service-based architecture |
| FDMA | frequency division multiple access | SC | successive cancellation |
| FEC | forward error correction | SCC | serially concatenated code |
| FSO | free space optical | SCM | spatial channel model |
| FTTA | fiber-to-the-antenna | SCMA | sparse code multiple access |
| GBSM | geometry-based stochastic model | SDN | software defined network |
| GFDM | generalized frequency division multiplexing | SFFT | symplectic finite Fourier transform |
| GPS | global positioning system | SGS | satellite ground station |
| HA | horn antenna | SIMO | single-input multiple-output |
| HAP | high altitude platform | SINR | signal-to-interference-plus-noise ratio |
| HARQ | hybrid automatic repeat request | SISO | single-input single-output |
| HCC | hybrid concatenated codes | SLC | satellite local controller |
| HD | high definition | SMF | session management function |
| HST | high-speed train | SNR | signal-to-noise ratio |
| IC | integrated circuits | SR | symbiotic radio |
| ICDT | information, communication, and data technology | S-V | Saleh-Valenzuela |
| ICI | inter-carrier interference | TCP | transmission control protocol |
| ICT | information and communication technology | TDD | time division duplex |
| IEN | intelligence endogenous network | TLC | terrestrial local controller |
| IETF | Internet engineering task force | THz | terahertz |
| ILDP | interactive learning design paradigm | ToF | THz-over-fiber |
| IoE | Internet of everything | TSAP | terrestrial-satellite access point |
| IoT | Internet of things | Tx | transmitter |
| IP | Internet protocol | UAV | unmanned aerial vehicle |
| IRS | intelligent reflection surface | UDM | unfied data management |
| ISFFT | inverse symplectic finite Fourier transform | UDN | ultra-dense network |
| ISM | industrial, scientific, and medical | UE | user equipment |
| IT | information technology | uHDD | ultra-high data density |
| ITS | intelligent transportation system | uHSLLC | ultra-high-speed with low-latency communications |
| ITU | international telecommunication union | | |
| KPI | key performance indicator | uMUB | ubiquitous mobile ultra-broadband |
| LAP | low altitude platform | UPF | user plane function |
| LDPC | low-density parity-check code | uRLLC | ultra-reliable and low latency communications |
| LED | light emitting diode | UTC-PD | uni-traveling-carrier photodiode |
| LEO | low Earth orbit | UV | ultraviolet |
| LO | local oscillator | V2V | vehicle-to-vehicle |
| LOS | line of sight | V2X | vehicle to everything |
| LTE | long term evolution | VHF | very high frequency |
| LTE-LAA | LTE license assisted access | VLC | visible light communication |
| LTE-U | LTE unlicensed | VNF | virtualized network function |
| LTE-V | long time evolution vehicle | VR | virtual reality |
| MAC | media access control | XR | extended reality |
| MAEC | multi-access edge computing | ZF | zero forcing |
